# Supplementary material for: Sox7‐positive endothelial progenitors establish coronary arteries and govern ventricular compaction
Source: EMBO Rep. 2023 Aug 8;24(10):e55043. doi: 10.15252/embr.202255043 (PMC10561369; doi:10.15252/embr.202255043)
Supplement: Supplementary file 2 — Expanded View Figures PDF [file EMBR-24-e55043-s004.pdf]

Expanded View Figures

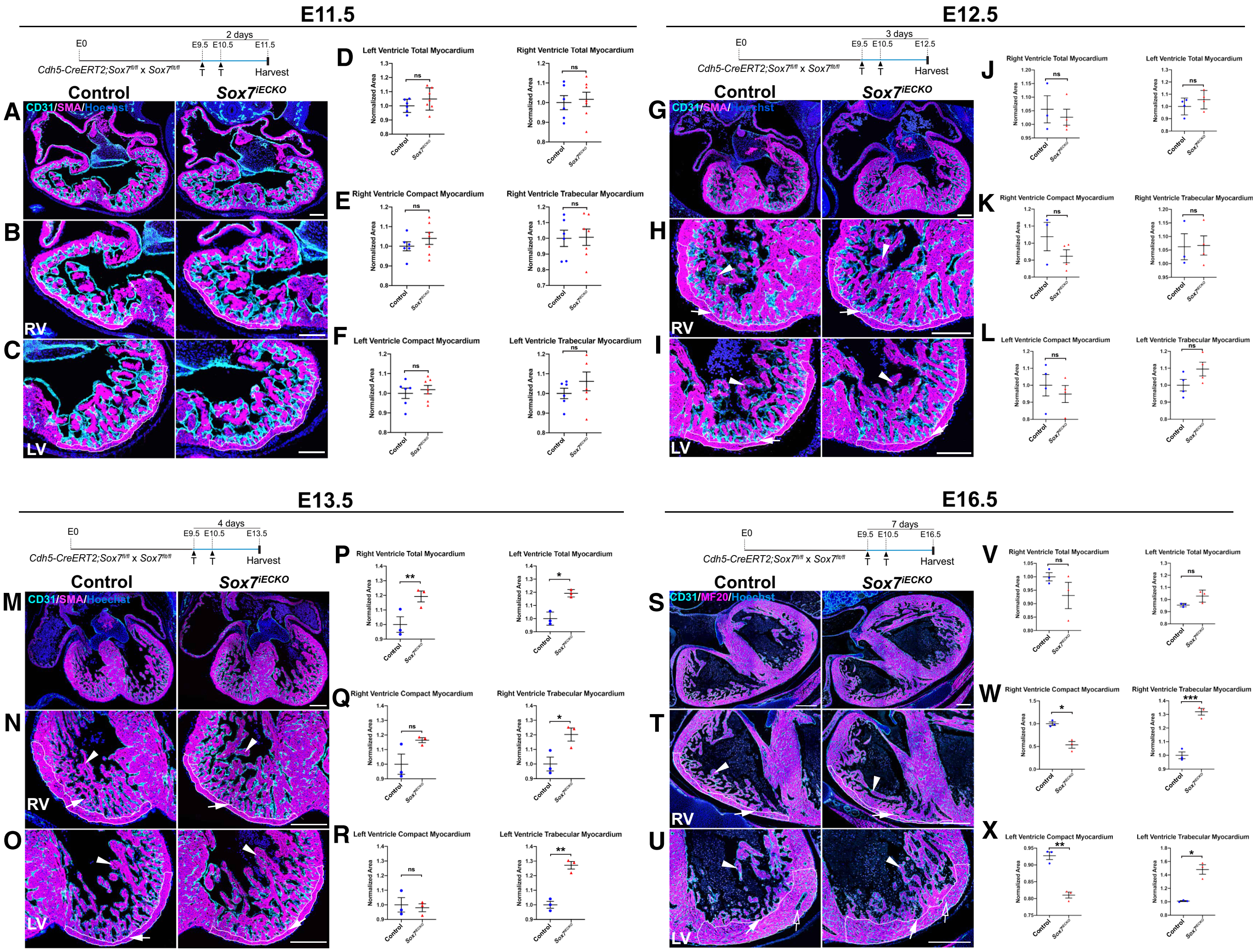

Figure EV1. Time-course morphological analysis of Sox7 endothelial loss-of-function embryos.

A–X (A–C, G–I, M–O, S–U) Morphological characterization of the cardiac defects present in *Sox7*^{iECKO} hearts compared to control hearts at E11.5 (A–C), E12.5 (G–I), E13.5 (M–O), and E16.5 (S–U) after Cre induction by tamoxifen injection at E9.5 and E10.5, by immunofluorescence with tissue-specific markers CD31 (endocardium/ endothelium; cyan), MF20 (myocardium; magenta), and nuclei (Hoechst; blue). (A, G, M, S) Low-magnification images showing the general heart morphology. (B, C, H, I, N, O, T, U) High-magnification images showing the morphology of the compact (arrows) and trabecular (arrowheads) myocardium, and coronary arteries (empty arrow) in the right (RV; B, H, N, T) and left (LV; C, I, O, U) ventricles. White contours in high-magnification panels highlight the compact myocardium area. Morphological defects are evident from E13.5 (M–O) and maintained at E16.5 (S–U). (D–F, J–L, P–R, V–X) Quantification of total (D, J, P, V), compact and trabecular myocardium (E, F, K, L, Q, R, W, X) area in right (E, K, Q, W) and left (F, L, R, X) ventricular chambers of *Sox7*^{iECKO} hearts compared to control hearts. Quantification results confirmed no significant difference in tissue area at E11.5 (D–F) and E12.5 (J–L). It also identified a significant increase in total and trabecular myocardium area at E13.5 (P–R) and the maintenance of the E14.5 cardiac defects at E16.5 (V–X). Biological replicates as per the number of data points in the graphs. Mean ± SEM; *t*-test. $P < 0.05$ (*); $P < 0.005$ (**); $P < 0.0005$ (***); ns = not significant. Scale bars = 100 μm (A–C), 150 μm (G–I), 200 μm (M–O), and 300 μm (S–U).

© 2023 The Authors

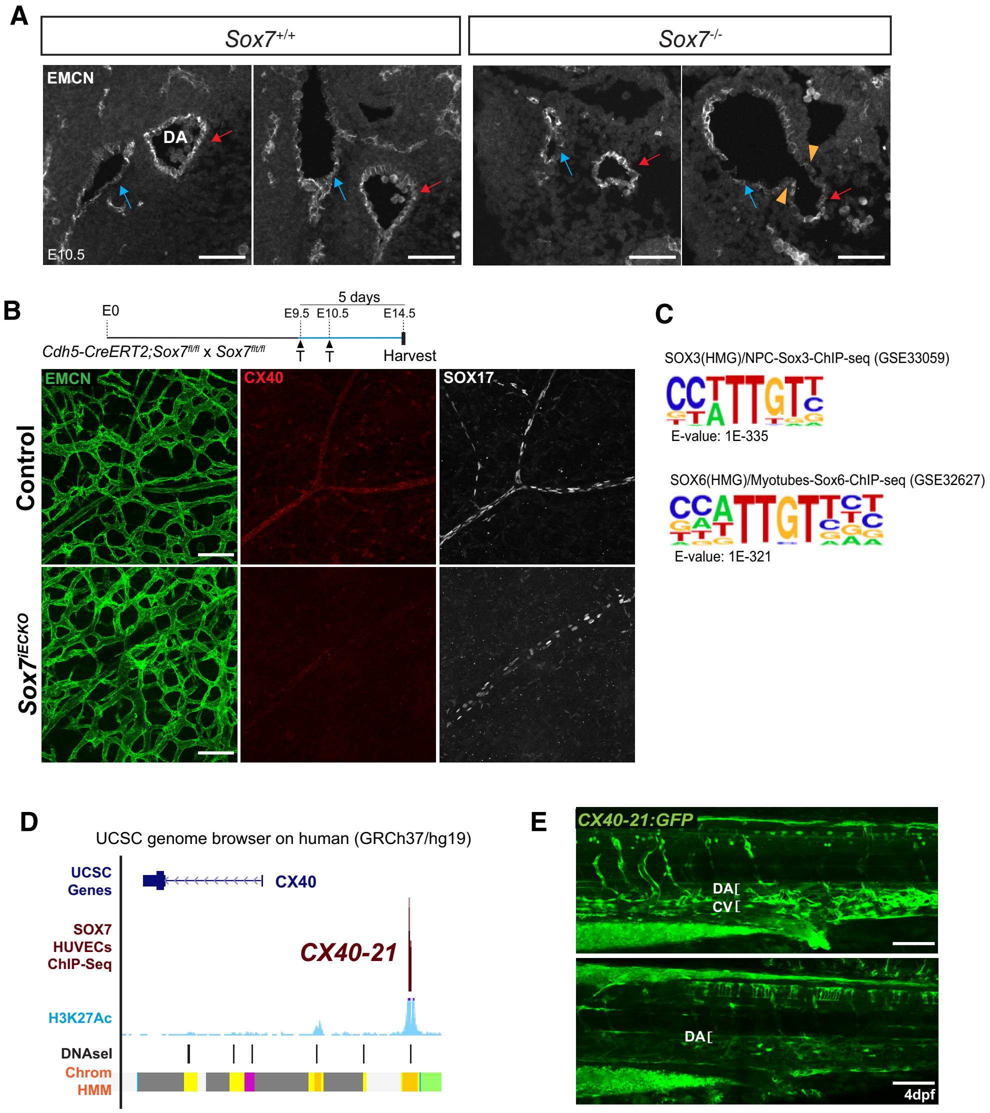

Figure EV2. SOX7 transcriptionally regulates the arterial specification marker, *Cx40*.

A Serial sections of a wild type and *Sox7*$^{-/-}$ hearts at E10.5, stained with endomucin (EMCN, white) to detect the endothelial lining. The dorsal aorta (DA) is labeled by red arrows, and the cardinal vein (CV) by blue arrows. Fusion between the DA and CV is indicated by orange arrowheads.

B Whole-mount immunostaining of *Sox7*^{iECKO} mutant and sibling control skin at E14.5, after Cre induction by tamoxifen injection at E9.5 and E10.5. The blood plexus is marked by endomucin (green), arteries by Cx40 (red), and SOX17 staining is shown in white.

C Schematic showing that SOX transcription motifs are the top binding motifs in SOX7 HUVECs ChIP-Seq data.

D Schematic representation of the human *CX40* locus showing a 500 bp putative regulatory element situated 21 kb upstream from the transcription start site (TSS) (denoted as *CX40-21* region) from UCSC Genome browser. The H3K27Ac is denoted in light blue, DNAseI hypersensitive hotspots are indicated by black/gray boxes, where the darkness is proportional to the maximum signal strength observed in any cell line. The chromatin state in HUVECs is shown in orange (indicates strong enhancer), green (weak transcribed), yellow (weak/poised enhancer), purple (inactive/poised promoter), and gray (polycomb repressed regions).

E The *CX40-21:GFP* transgene directs GFP fluorescence expression to vascular endothelium in transgenic zebrafish larvae at 4 dpf.

Data information: DA, dorsal aorta; CV, cardinal vein. Scale bars = 100 μm.

© 2023 The Authors

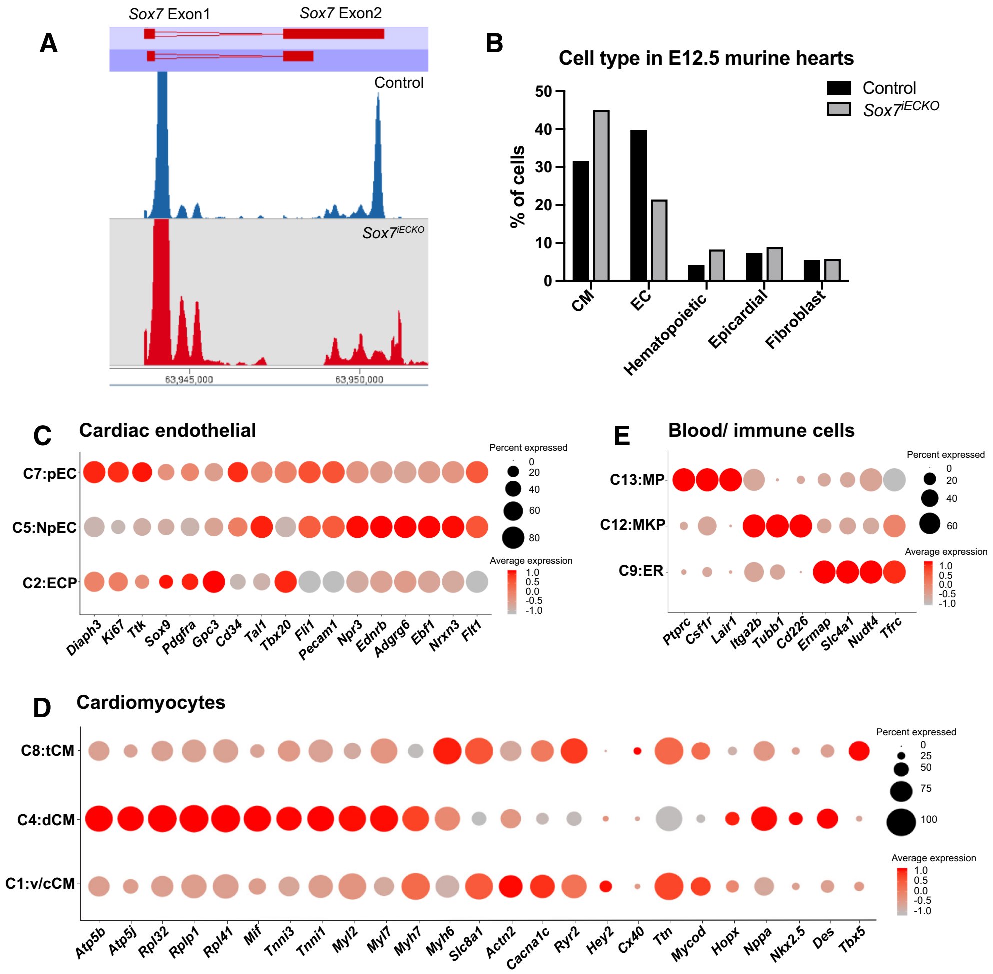

Figure EV3. Cell composition and marker gene expression profiles of cardiac populations in *Sox7*^{iECKO} hearts.

A There is a lack of transcripts harboring the exon 2 (floxed exon) of the *Sox7* locus in the *Sox7*^{iECKO} hearts (red track).

B Graphs showing the different cell types comprising E12.5 control and *Sox7*^{iECKO} hearts.

C–E Dot plots showing expression of genes that define each subcluster within major cell type population: cardiac endothelial cells (C), cardiomyocytes (D), and blood/immune cells (E).

© 2023 The Authors

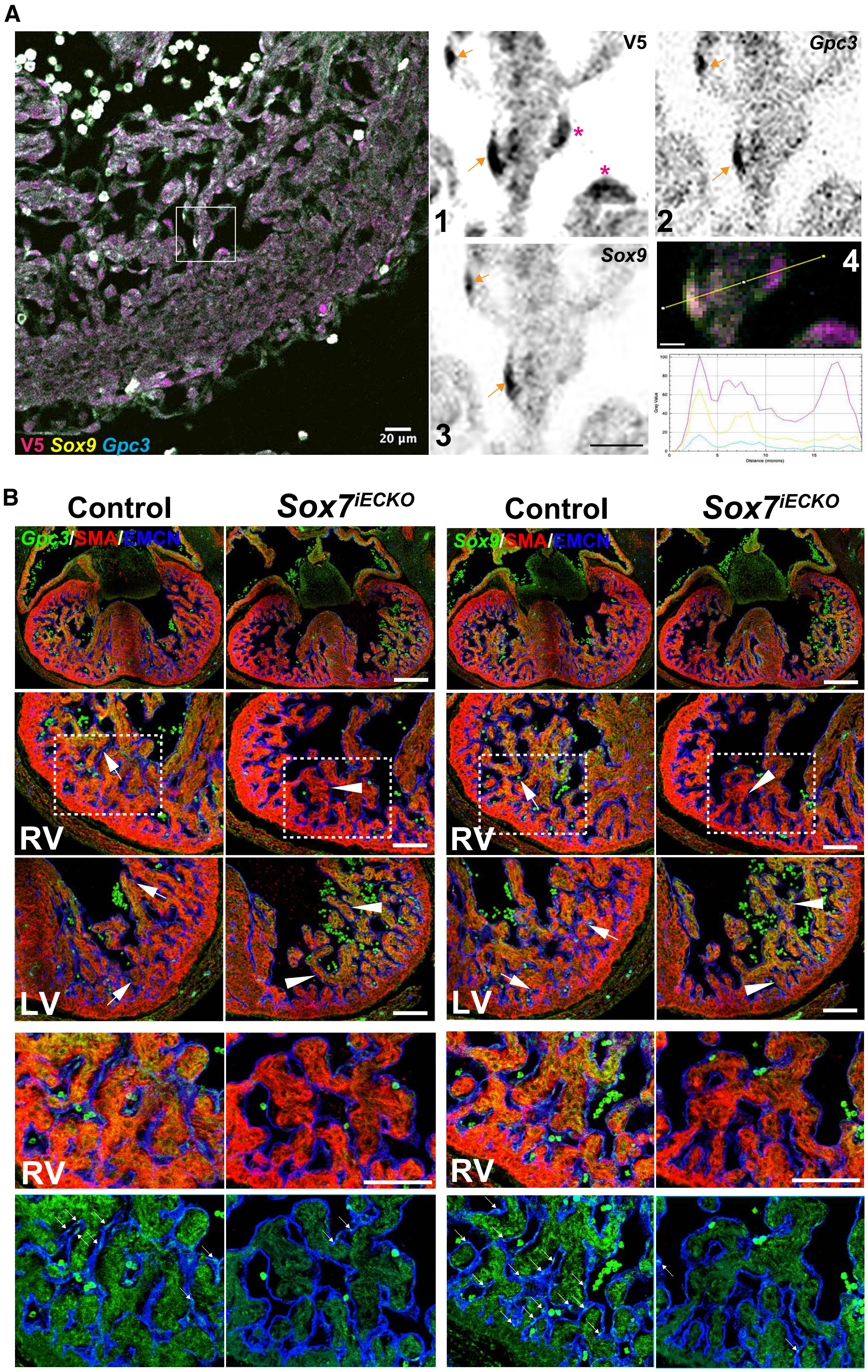
A
V5 Sox9 Gpc3
20 µm
V5
Gpc3
Sox9
1
2
3
4
B
Control
Sox7iECKO
Control
Sox7iECKO
Gpc3/SMA/EMCN
Sox9/SMA/EMCN
RV
LV
RV
LV
RV
RV

Figure EV4.

Figure EV4. Triple-positive Gpc3, Sox9, and Sox7 endocardial cells in 12.5 developing hearts are depleted in the Sox7^{iECKO}.

A smFISH for *Gpc3* and *Sox9* combined with V5 immunofluorescence in *Sox7-V5* transgenic reporter embryos shows the presence of a subset of trabecular endocardial cells that are triple positive for SOX7, *Sox9*, and *Gpc3* (orange arrows). Pink asterisks, SOX7 single-positive cells. High magnification from inset with 1, 2, and 3 showing individual channels for each marker; 4 shows the plot profile of fluorophore distribution for triple- or single-positive cells. Scale bar in 3 and 10 μm.

B Expression pattern of C2 population markers. Analysis by smFISH of the C2 endocardial markers *Gpc3* (left panels), *Sox9* (right panels) in combination with immunofluorescence of endocardial (endomucin, blue), and myocardial (SMA, red) markers on tissue sections of control and *Sox7*^{iECKO} mutant hearts. The first row panels are low-magnification images showing the entire heart tissue. The second row panels show images of the right ventricles. The third row panels show the left ventricle. The two bottom row panels show a high magnification from inset in RV panels and reveal the loss of *Gpc3* and *Sox9* expression in the endocardium of the *Sox7*^{iECKO}. Markers (green), myocardium (red), and endocardium (blue). Scale bars = 200 μm (top panels), 100 μm (middle and bottom panels), arrows indicate *Gpc3*- and *Sox9*-positive endocardium in the control embryos; arrowheads indicate lack of expression of *Gpc3* and *Sox9* in the endocardium of *Sox7*^{iECKO} embryos.

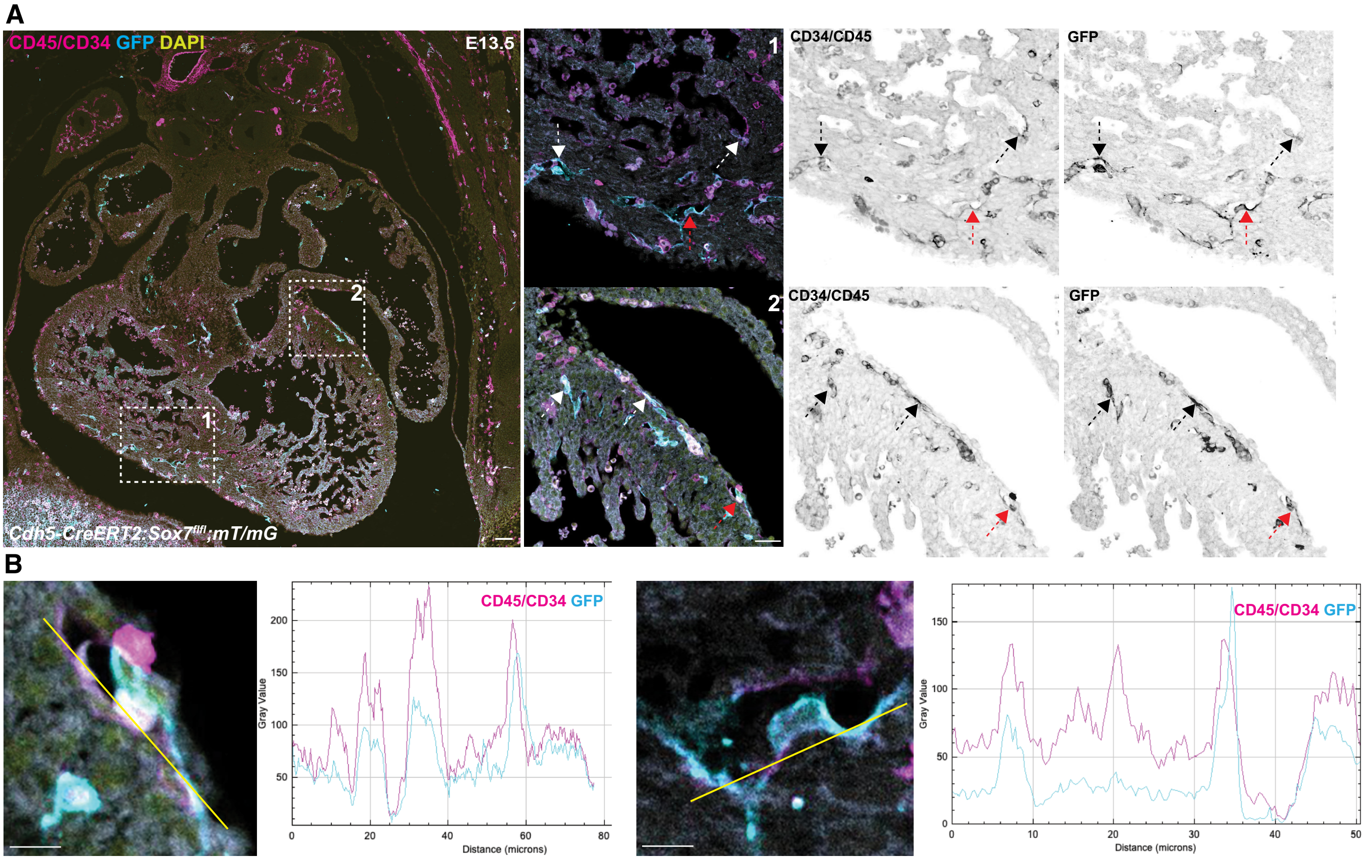

Figure EV5. Sox7-null endothelial cells transdifferentiate into hematopoietic cell lineages in vivo.

A Transverse section of an E13.5 heart from a triple transgenic *SOX7*^{iECKO}; *mT/mG* embryo stained for GFP, CD45/CD34 (same fluorophore), and DAPI. This staining shows the presence of GFP-positive endothelial cells in the coronary vasculature that express either CD34 or CD45 hematopoietic markers (red and black or white arrows). Scale bar = 20 μm.

B Plot profile analysis of a cell from the inner lining of a vessel showing the colocalization of the fluorescent signal. These regions are blown-up areas indicated by the red arrows. Scale bar = 10 μm.

 © 2023 The Authors
