# Supplementary material for: Sox7‐positive endothelial progenitors establish coronary arteries and govern ventricular compaction
Source: EMBO Rep. 2023 Aug 8;24(10):e55043. doi: 10.15252/embr.202255043 (PMC10561369; doi:10.15252/embr.202255043)
Supplement: Supplementary file 1 — Appendix [file EMBR-24-e55043-s001.pdf]

Appendix table of content

Figure legends S1 to S9……………………………………………………………….2-4
Figures S1 to S9…………………………………………………………………….....5-13

Appendix figures legends

Appendix Figure S1. Time-course cell count and proliferation analysis of *Sox7* endothelial loss of function embryos.

(**A-C**) Proliferation analysis by Ki67 immunofluorescence on tissue sections from *Sox7*^{iECKO} hearts (left panels) compared to control (right panels) at E12.5 (A-A''), E13.5 (B-B'') and E14.5 (C-C''). Images corresponding to low magnification showing the entire heart (A-C) and higher magnification showing the right (A'-C') and left (A''-C'') ventricles. Ki67 staining (red), nuclei (blue) and myocardial transparent mask (green). Scale bars = 300 µm (A,C), 200µm (B,C',C''), 150µm (A',A'') and 100µm (B',B'').

(**D-M**) Quantification of the number of cells forming the ventricular chamber (D, I), chamber myocardium (E, J), chamber endocardium (F, K), and the regional subdivision of the chamber endocardium into apical (G, L) and basal (H, M) endocardium at E12.5, E13.5 and E14.5 in *Sox7*^{iECKO} hearts compared to control hearts. No significant changes were observed in these parameters and stages besides a significant decrease in endocardial cell numbers mainly in the basal endocardium (H, K, M).

(**N-W**) Quantification of the number of Ki67^{+} proliferating cells in the ventricular chamber (N, S), chamber myocardium (O, T), chamber endocardium (P, U), and the regional subdivision of the chamber endocardium into apical (Q, V) and basal (R, W) endocardium at E12.5, E13.5 and E14.5 in *Sox7*^{iECKO} hearts compared to control hearts. Results show reduced cell proliferation in the left chamber (N) and in the myocardium of both chambers (O, T) at E14.5, and a significant increase in cell proliferation in the apical endocardium at E13.5 (Q).

Data information: Biological replicates as per the number of data points in the graphs. Mean ± SEM; *t*-test. P <0.05 (*); P<0.005 (**); P<0.0005 (***); ns = not significant.

Appendix Figure S2. Whole-mount immunostaining of control and *Sox7*^{iECKO} hearts at E14.5 after Cre induction by tamoxifen injection at E9.5, E10.5.

Coronary vessels are stained with VEGFR2 (green), coronary arteries (arrows) and trabecular myocardium (TM) are marked with CX40 (red). Asterisks indicate where the coronary arteries are lost in *Sox7*^{iECKO} heart. Scale bars = 100 µm.

Appendix Figure S3. Morphometric quantification of epicardial and intramyocardial coronary vessel phenotype.

(A-D) (**A**, **C**) Immunofluorescence images similar to the ones showed in Fig. 2, highlighting the epicardial and coronary vessel phenotypes comparing Control (left panels) and *Sox7*^{iECKO} (right panels) hearts at E14.5 (A) and E16.5 (C). Myocardium (MF20, magenta), endocardium (CD31, cyan), nuclei (Hoechst, blue). Arrows point at the intra-myocardial coronary vessels. Scale bars = 200µm (A) and 150µm (C). (**B**, **D**) Graphs showing the morphological quantifications of intramyocardial coronary vessel area (left), subepicardial area (middle) and subepicardial vessel area (right) at E14.5 (B) and E16.5 (D). Biological replicates as per the number of data points in the graphs. Mean ± SEM; *t*-test. P <0.05 (*); P<0.005 (**); P<0.0005 (***);P<0.0001 **(****)** ns = not significant.

Appendix Figure S4. Genome-wide transcriptomic analysis of *Sox7* loss of function conditions.

(A) Heatmap showing the gene expression profile from control and *Sox7*$^{\Delta ex2/\Delta ex2}$ whole-embryo RNA-Seq at E8.5. 15 genes were downregulated and 50 were upregulated in the mutant samples.

(B) Gene ontology analysis by DAVID bioinformatics database, showing the top biological processes associated to the differentially expressed genes in SOX7 E8.5 RNA-Seq data. The top 2 biological processes include blood vessel development (*Egfl7, Cdx4, Cx37, Cx40, Lama4*) and angiogenesis (*Egfl7, Flt4, Rapgef3, Tbx4, Dll4, Cx40, Mmrn2*).

(C) Heatmap showing the gene expression profile from RNA-Seq performed on sorted CD31^{+} endothelial cells from E10.5 control and *Sox7*^{iECKO} whole embryos, injected with tamoxifen at E9.5 to activate endothelial specific *Sox7* deletion.

Appendix Figure S 5. Expression pattern of C4 population markers.

(A-F) Expression pattern analysis by smFISH of the C4 myocardial markers Hopx (A-C) and Nppa (D-F) in combination with immunofluorescence of endocardial (endomucin) and myocardial (SMA) markers on tissues sections of Control (A-C) and *Sox7*^{iECKO} mutant (D-F) hearts. Low magnification images showing the entire heart tissue (A-F). Marker (green), myocardium (red), endocardium (blue). Scale bars = 100 μm. The green panels show the signal for *Hopx* and *Nppa* only. Arrows point at the expression of the marker whereas arrowheads point at the lack of expression of the marker in control embryos at the trabecular base.

(G) Cell population of the compact myocardium (C1) showing the expression levels of *Mycn*, *Kcnd2* and *Hey2* from snRNA-Seq analysis. Comparison between control and *Sox7*^{iECKO} shows a decrease in the number of cells expressing these 3 markers in the mutant embryos.

Appendix Figure S6. Pseudotime trajectory analysis of cells in control and *Sox7*^{iECKO} hearts.

(A) Pseudotime analysis graphs of cells in all 13 identified clusters in control (left) and *Sox7*^{iECKO} (right) hearts.

(B) Pseudotime analysis graphs of cells in all 12 identified populations in control (left) and *Sox7*^{iECKO} (right) hearts, after removing the C2: Endocardial progenitors (ECP) population.

Appendix Figure S7. Lineage tracing analysis indicates that Sox7-null endothelial cells do not give rise to cardiomyocytes.

Immunofluorescence staining in transverse heart sections of *Sox7*^{iECKO}; *mT/mG* mutant and *Cre+; mT/mG* control embryos at E14.5, after injection with tamoxifen at E9.5 and E10.5. Blood vessels are stained with endomucin, EMCN (magenta), cells after Cre excision are shown by EGFP expression (green), and cardiomyocytes are marked by tropomyosin, TPM (blue). No EGFP/TPM double positive cells were found in the *Sox7*^{iECKO}. Scale bars = 100 μm.

Appendix Figure S8. Triple negative $CD71^-CD45^-CD31^-$ control cell population remains unchanged in Sox7 loss of function.

(A) Flow analysis of the $CD71^-CD45^-CD31^-$ control cell population, revealing that it remains unchanged in E13.5 *Sox7*^{iECKO} hearts relative to controls.

(B) Graphs showing the proportion of $CD71^-CD45^-CD31^-$ control population normalized to the $CD71^-CD45^{-/low}$ parent events.

Of note the panel shown here is duplicated from Figure 6B, to emphasise on a different cell population.

Data information: Biological replicates, n = 6 control hearts; n = 8 *Sox7*^{iECKO} hearts. Mean ± SEM; *t*-test, ns = not significant.

Appendix Figure S9 Multiplexed imaging method.

Top panel: Schematic depicting the serial sections and the different combinations of markers used to analyse the proliferative states of different cell types (eg either endothelial or cardiomyocytes).

Bottom panels: **(A)** Fluorescence image of left ventricle showing myocardium (SMA; magenta) and endocardium (CD31; cyan). **(B)** Tissue masks of myocardial and endocardial regions generated via intensity thresholding. **(C)** Myocardial mask with manually selected compact myocardial layer. **(D)** Layered myocardial mask depicting compact myocardium in addition to basal and apical trabecular layers, as determined from the compact layer selection. **(E)** Nuclear staining (Hoechst 33342). **(F)** Nuclear segmentation label generated by StarDist plugin in Fiji. **(G)** Ki67 immunofluorescence of cellular proliferation. **(H)** Classification of nuclei as either $Ki67^+$ (active; red) or $Ki67^-$ (blue) via average intensity thresholding. **(I)** Tissue classification of inactive/active myocardial nuclei. **(J)** Tissue classification of inactive/active endocardial nuclei.

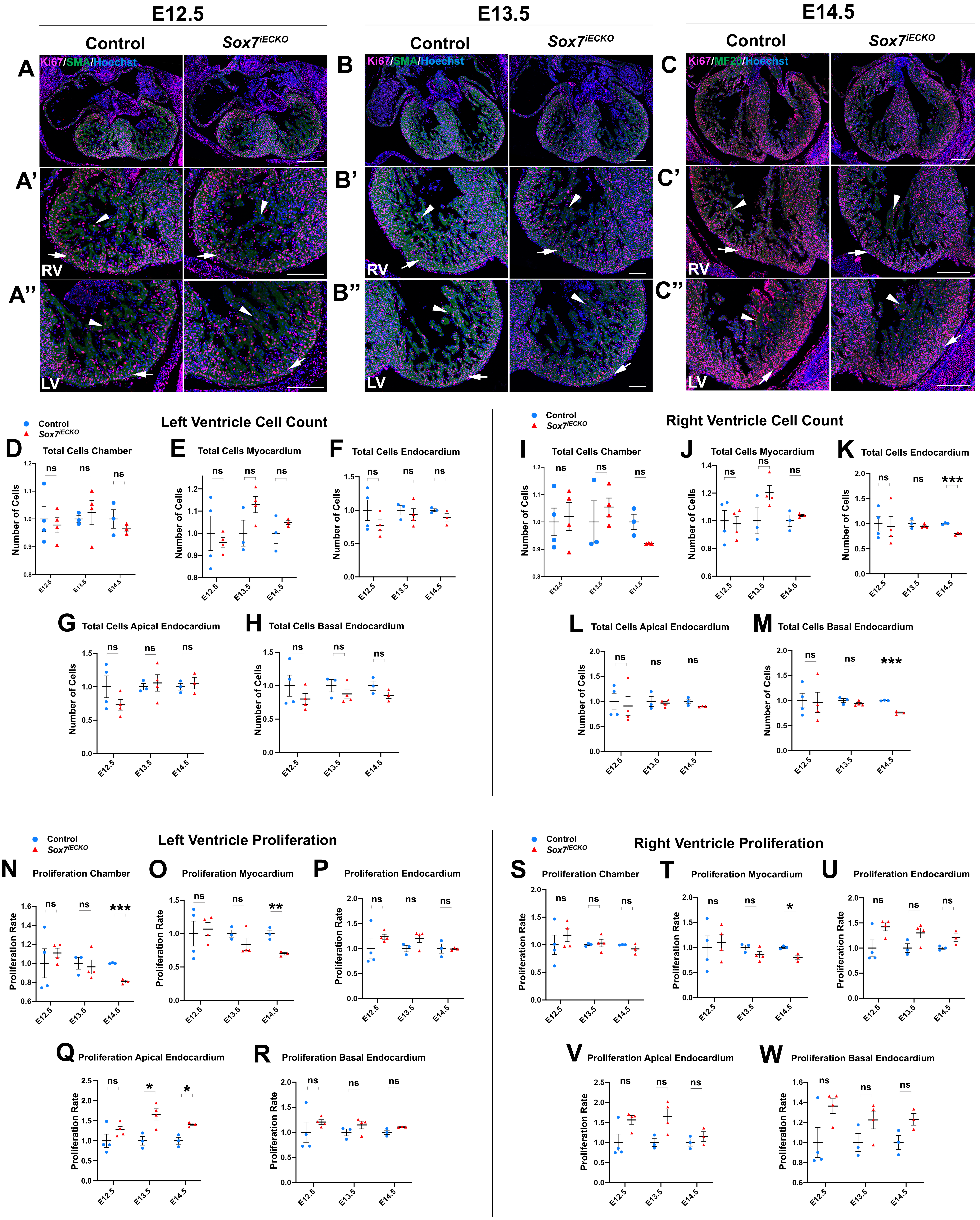

Appendix Fig. 1

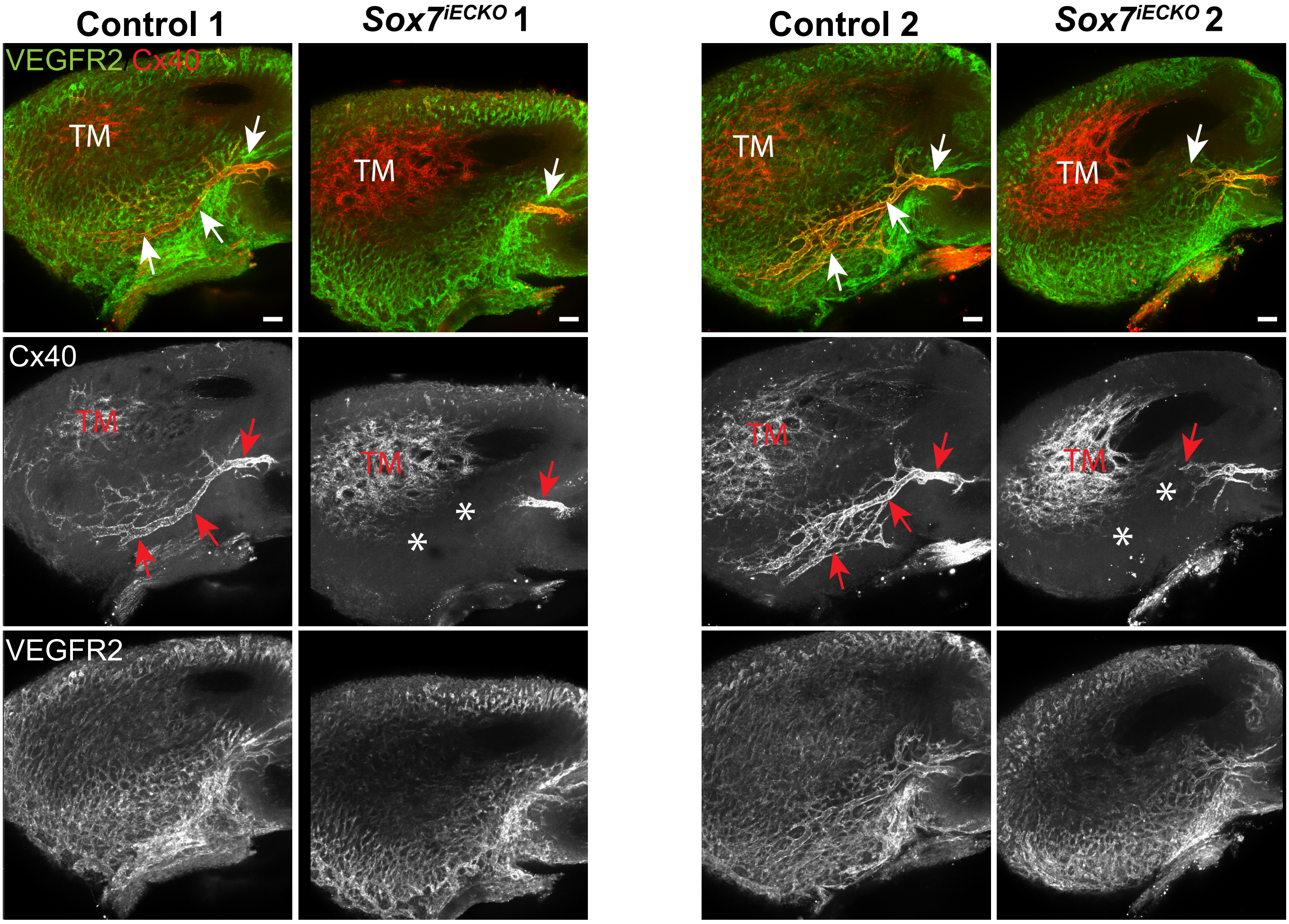

Appendix Fig.2

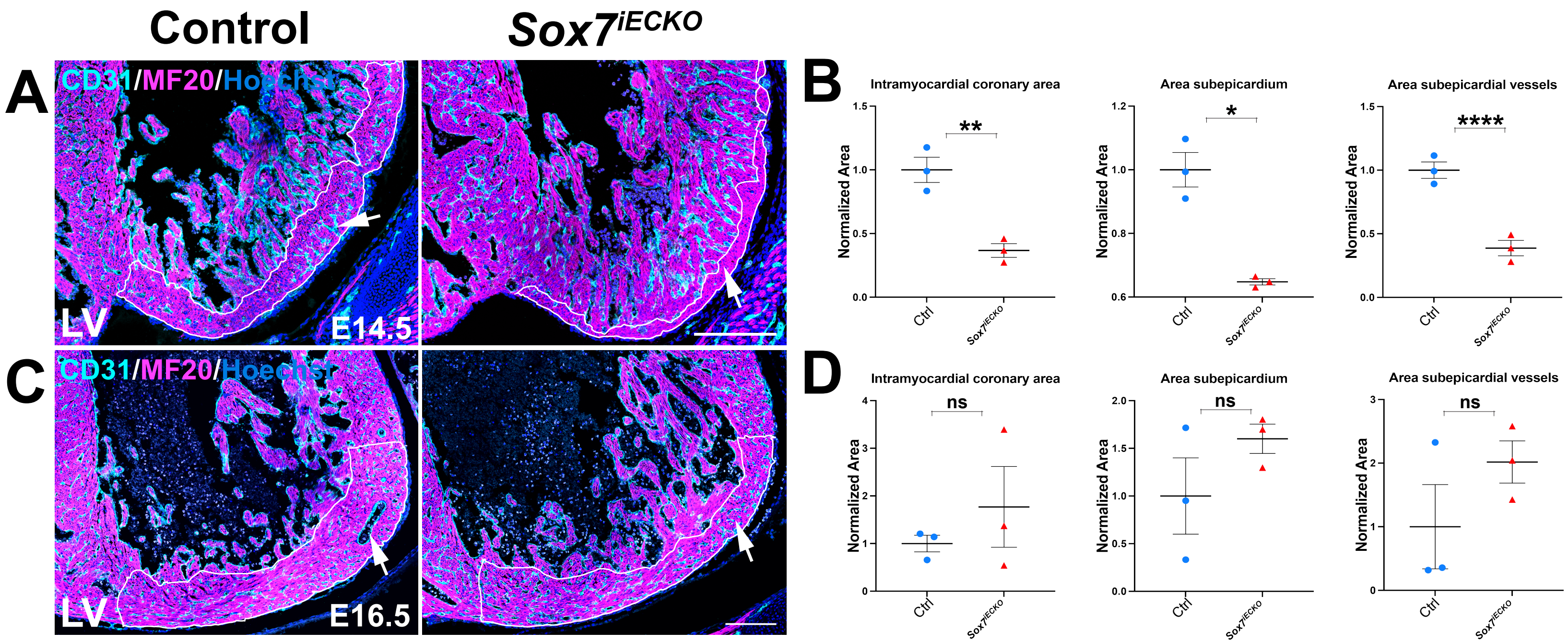

Appendix Fig.3

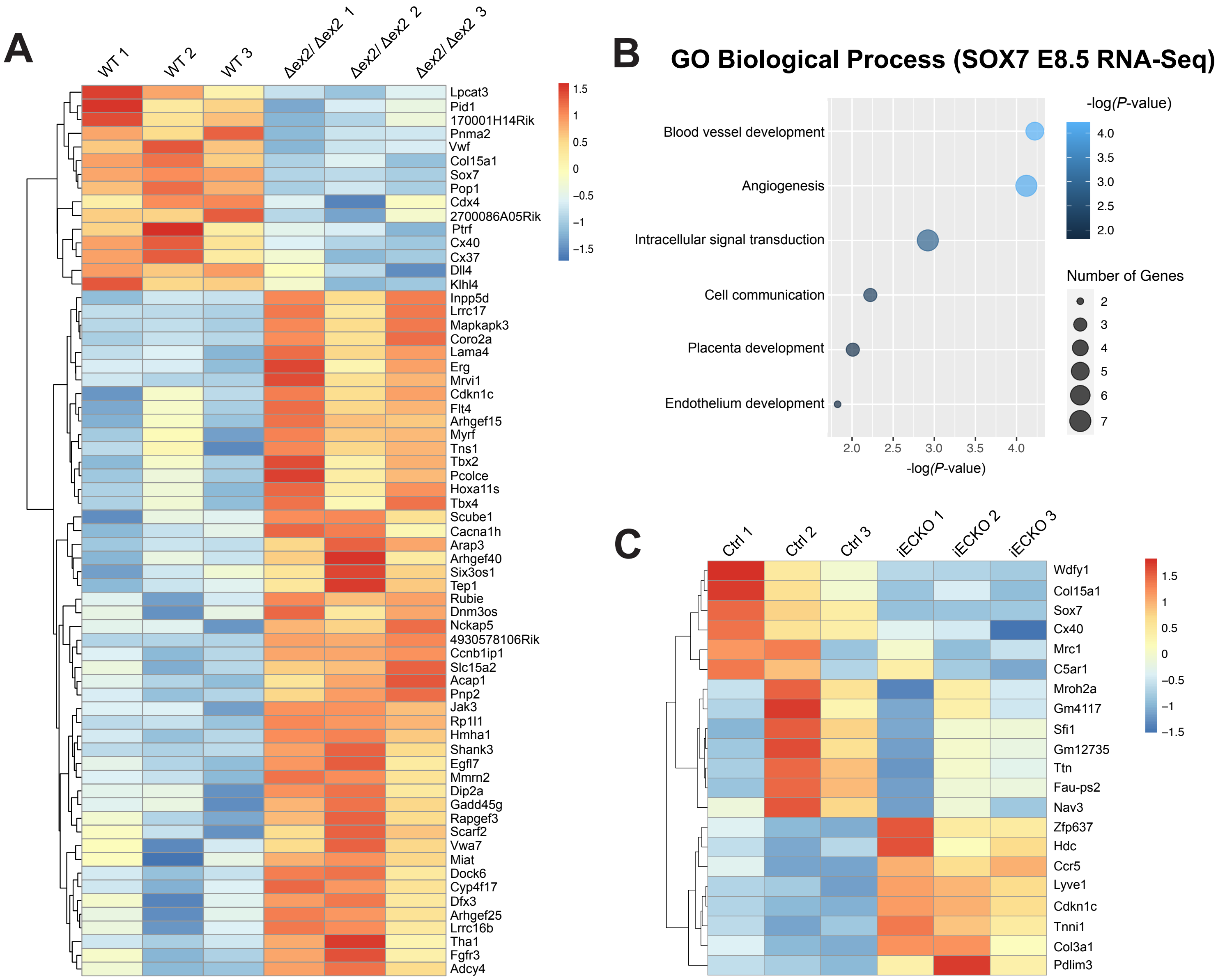

Appendix Fig.4

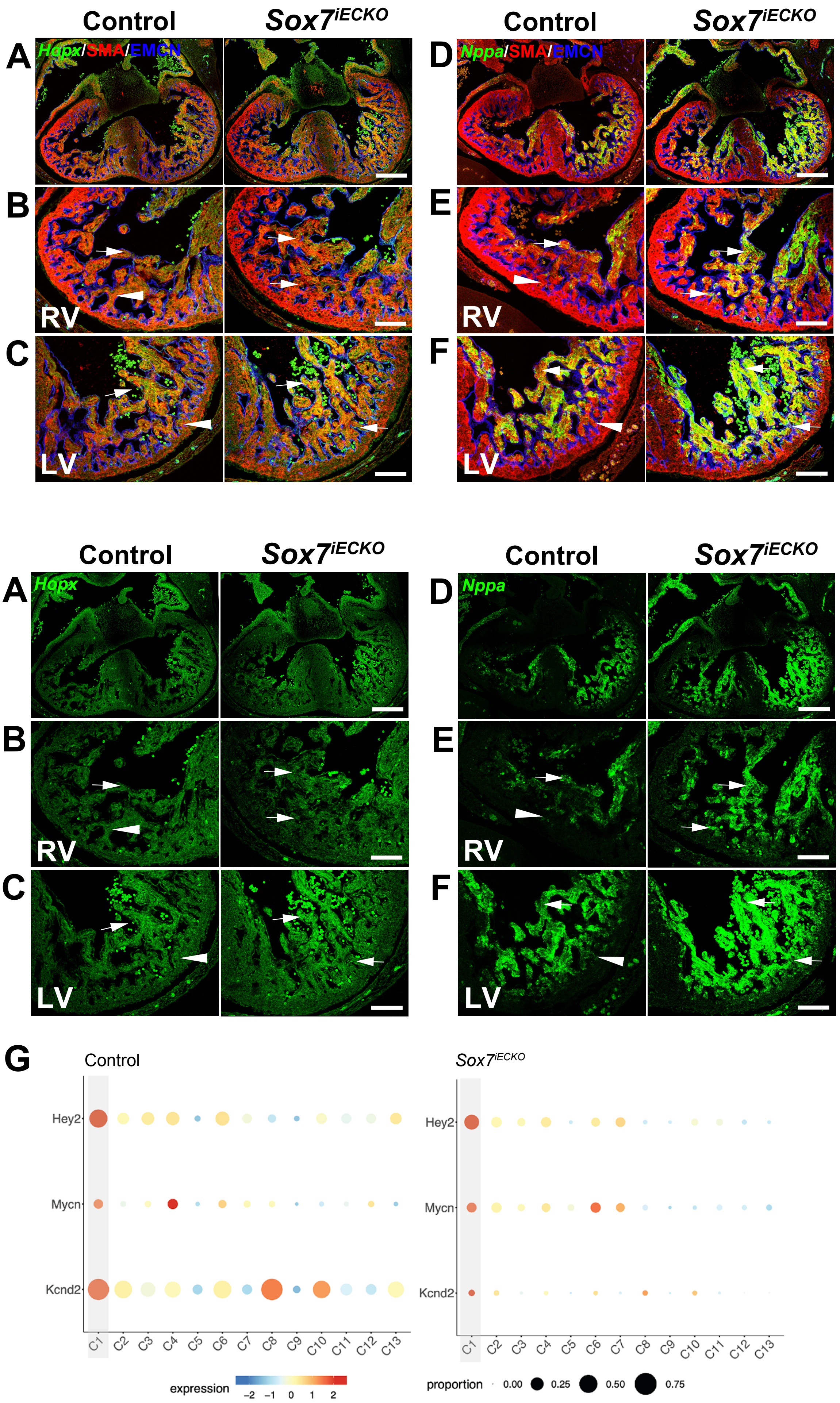

Appendix Fig. 5

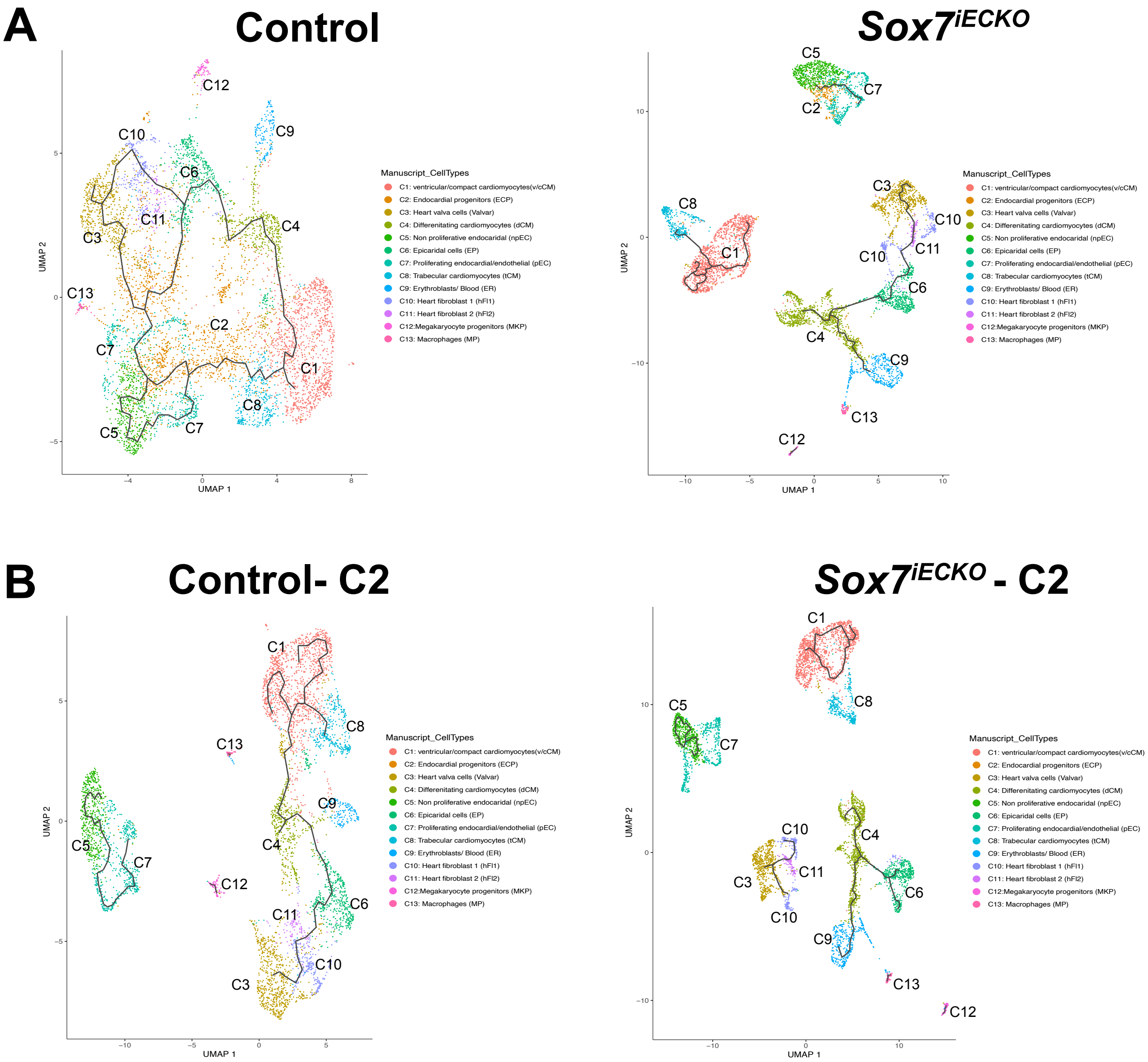

Appendix Fig.6

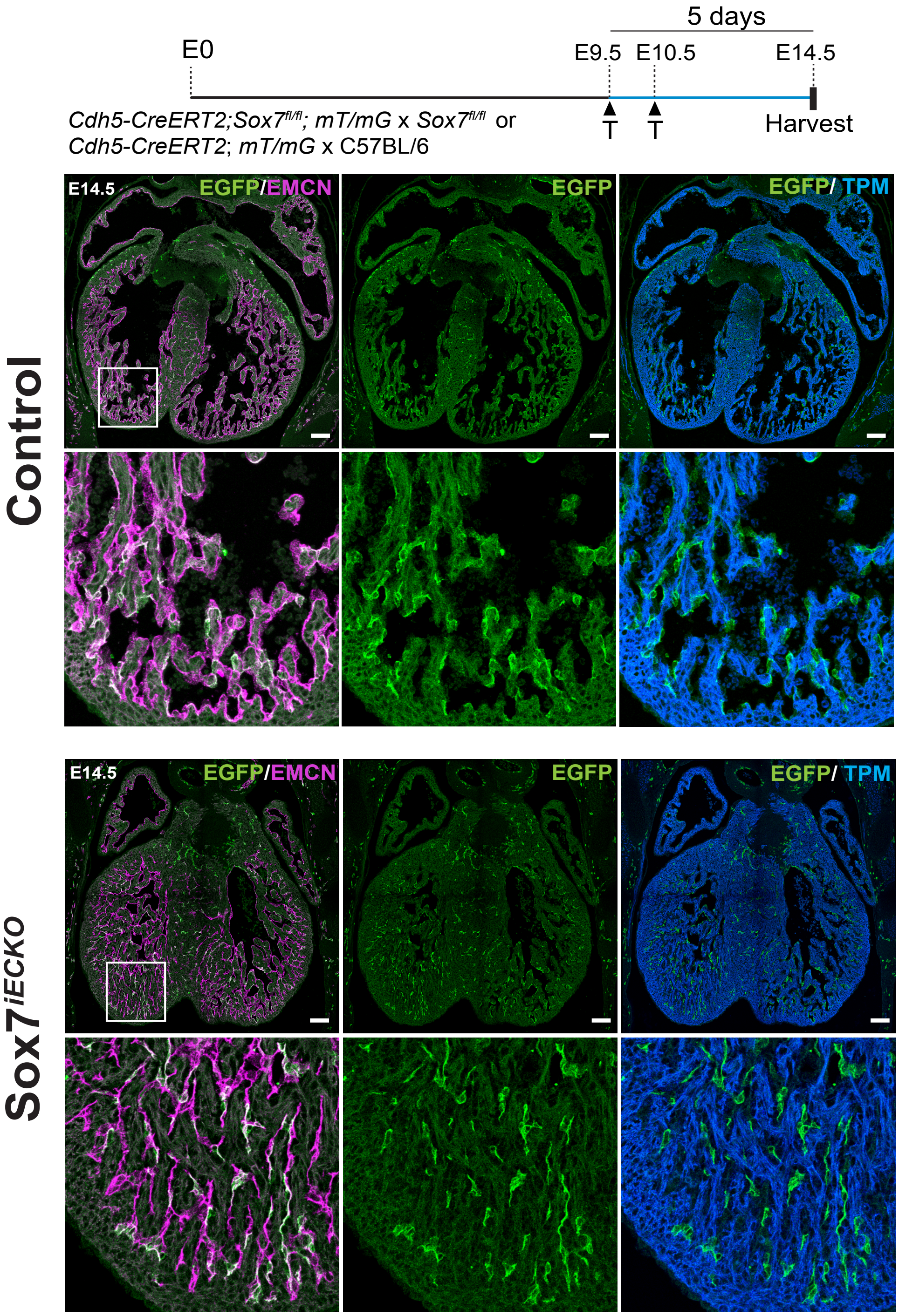

Appendix Fig.7

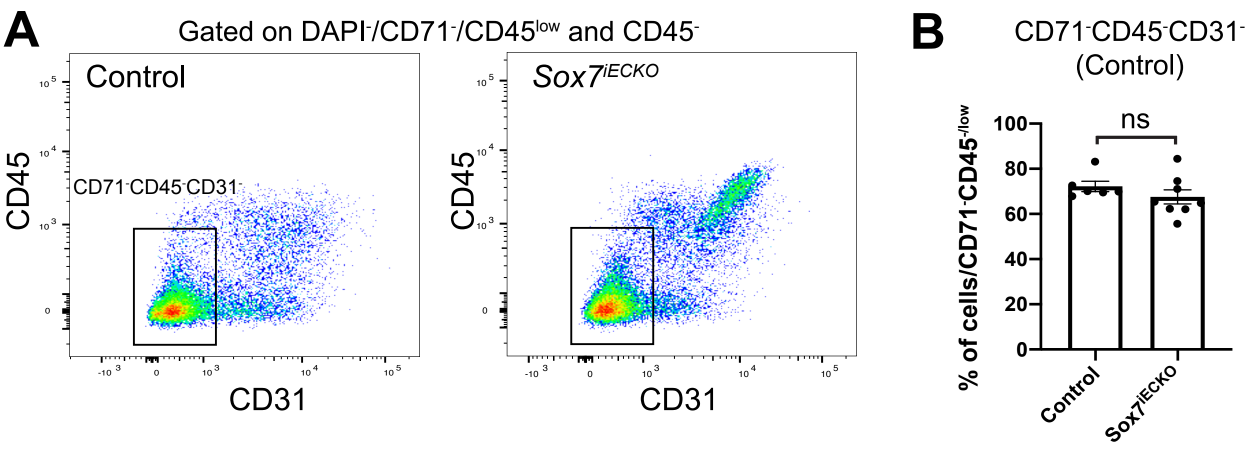

Appendix Fig.8

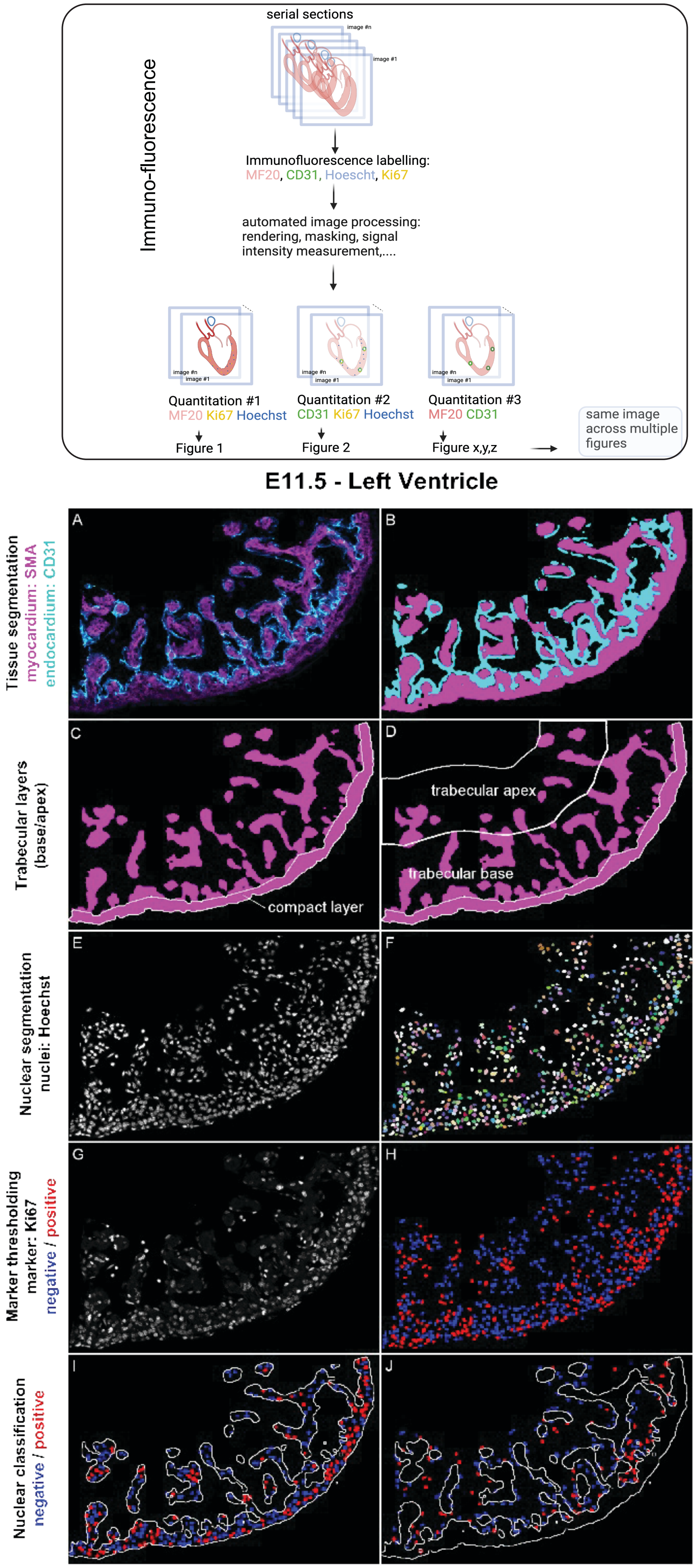

Appendix Fig.9
