# Supplementary material for: Sox7‐positive endothelial progenitors establish coronary arteries and govern ventricular compaction
Source: EMBO Rep. 2023 Aug 8;24(10):e55043. doi: 10.15252/embr.202255043 (PMC10561369; doi:10.15252/embr.202255043)
Supplement: Supplementary file 3 — PDF+ [file EMBR-24-e55043-s002.pdf]

Article

TRANSPARENT PROCESS · OPEN ACCESS

EMBO reports

Sox7-positive endothelial progenitors establish coronary arteries and govern ventricular compaction

Ivy KN Chiang[1], David Humphrey[2], Richard J Mills[3], Peter Kaltzis[4], Shikha Pachauri[1], Matthew Graus[1], Diptarka Saha[4], Zhijian Wu[4], Paul Young[2], Choon Boon Sim[5], Tara Davidson[1], Andres Hernandez-Garcia[6], Chad A Shaw[6], Alexander Renwick[6], Daryl A Scott[6], Enzo R Porrello[5,7,8], Emily S Wong[2], James E Hudson[3], Kristy Red-Horse[9], Gonzalo del Monte-Nieto[4,*] & Mathias Francois[1,**]

Abstract

The cardiac endothelium influences ventricular chamber development by coordinating trabeculation and compaction. However, the endothelial-specific molecular mechanisms mediating this coordination are not fully understood. Here, we identify the Sox7 transcription factor as a critical cue instructing cardiac endothelium identity during ventricular chamber development. Endothelial-specific loss of *Sox7* function in mice results in cardiac ventricular defects similar to non-compaction cardiomyopathy, with a change in the proportions of trabecular and compact cardiomyocytes in the mutant hearts. This phenotype is paralleled by abnormal coronary artery formation. Loss of Sox7 function disrupts the transcriptional regulation of the Notch pathway and connexins 37 and 40, which govern coronary arterial specification. Upon Sox7 endothelial-specific deletion, single-nuclei transcriptomics analysis identifies the depletion of a subset of Sox9/Gpc3-positive endocardial progenitor cells and an increase in erythro-myeloid cell lineages. Fate mapping analysis reveals that a subset of Sox7-null endothelial cells transdifferentiate into hematopoietic but not cardiomyocyte lineages. Our findings determine that Sox7 maintains cardiac endothelial cell identity, which is crucial to the cellular cross-talk that drives ventricular compaction and coronary artery development.

Keywords arterial cell fate; coronary vessels; heart development; non-compaction cardiomyopathy; SOX transcription factor

Subject Categories Cardiovascular System; Development; Vascular Biology & Angiogenesis

DOI 10.15252/embr.202255043 | Received 14 March 2022 | Revised 12 July 2023 | Accepted 24 July 2023 | Published online 8 August 2023

EMBO Reports (2023) 24: e55043

Introduction

The mammalian heart is composed of a range of different cell types including cardiomyocytes, fibroblasts, endothelial cells, and perivascular cells. Although cardiomyocytes occupy 70–85% of the heart volume, recent studies showed that cardiac endothelial cells are the most abundant cells in both murine and human hearts (~60%) (Pinto *et al*, 2016). Cardiac endothelial cells contribute to the endocardium (the inner cell layer of the heart), and the endothelium which forms the inlet and outlet vessels of the coronary plexus that comprises lymphatics, veins, arteries, and capillaries (Colliva *et al*, 2020). Given the close proximity of the endothelial lining and cardiomyocytes, factors secreted from cardiac endothelial cells have the potential to modulate cardiomyocyte contractile behavior, shape, and function. In support of this cross-signaling activity, recent studies have identified the endocardium as the critical tissue controlling the morphogenetic processes taking place during cardiac trabeculation (Del Monte-Nieto *et al*, 2018; Qu *et al*, 2019). In particular, Del Monte-Nieto *et al* (2018) identified endocardial Notch and Nrg1 pathways as key regulators, respectively, of endocardial

1 Centenary Institute, Royal Prince Alfred Hospital, The University of Sydney, Sydney, NSW, Australia
2 The Victor Chang Cardiac Research Institute, Darlinghurst, NSW, Australia
3 QIMR Berghofer Medical Research Institute, Brisbane, QLD, Australia
4 The Australian Regenerative Medicine Institute, Monash University, Clayton, VIC, Australia
5 The Murdoch Children's Research Institute, Royal Children's Hospital, Melbourne, VIC, Australia
6 Department of Molecular and Human Genetics, Baylor College of Medicine, Houston, TX, USA
7 Melbourne Centre for Cardiovascular Genomics and Regenerative Medicine, The Royal Children's Hospital, Melbourne, VIC, Australia
8 Department of Anatomy and Physiology, School of Biomedical Sciences, The University of Melbourne, Melbourne, VIC, Australia
9 Department of Biology, Stanford University, Stanford, CA, USA
*Corresponding author. Tel: +61 399053738; E-mail: gonzalo.delmontenieto@monash.edu
**Corresponding author. Tel: +61 28627 9497; E-mail: m.francois@centenary.org.au

© 2023 The Authors. Published under the terms of the CC BY 4.0 license

extracellular matrix (ECM) degradation and myocardial ECM synthesis processes that are critical for trabecular myocardium growth and organization. These data suggest that the highly dynamic process of trabecular myocardium morphogenesis relies on a critical and tightly controlled interplay between endothelial cells and cardiomyocytes (Del Monte-Nieto *et al*, 2018).

Given the essential role of cardiac endothelial cells in controlling the interplay of differential cell type activity during heart formation, a central question remains as to whether endothelial cell identity and differentiation state are key prerequisites for cardiac morphogenesis. While endocardial cells are differentiated by E8.5, it is now known that the cardiac endothelial phenotype is highly plastic. During heart formation, both sinus venosus endothelium and ventricular endocardium are the source of cells for the endothelium forming the coronary vessels (Red-Horse *et al*, 2010; Wu *et al*, 2012). In the endocardial cushions, differentiated endocardial cells transdifferentiate into mesenchymal cells, ultimately giving rise to cardiac valves and septa (de Lange *et al*, 2004; Lincoln *et al*, 2004). In addition, a subset of endocardial cells in the outflow tract and atria will contribute to myeloid and erythroid cells during embryogenesis (Nakano *et al*, 2013). This suggests that maintenance of endocardial cell identity is finely regulated upon cell specification, ensuring only a subpopulation will acquire the potential to form other cardiac cell types without compromising the integrity of heart tissue homeostasis.

Although a handful of transcription factors and signaling pathways have been shown to induce early endocardial cell fate specification (Ferdous *et al*, 2009; Kataoka *et al*, 2011), little is known about those regulators necessary to maintain endocardial commitment and direct subsequent, specialized cell differentiation. The SOXF group of transcription factors (*Sox7*, *Sox17*, and *Sox18*) are known key players of endothelial cell differentiation and specification (François *et al*, 2008; Corada *et al*, 2013; Chiang *et al*, 2017). In the heart, SOXF group members are widely expressed in cardiac tissues in progenitor populations of distinct cell lineages (e.g., cardiac and endothelial/endocardial) (Sakamoto *et al*, 2007; González-Hernández *et al*, 2020). Studies in Xenopus have implicated *sox7* and *sox18* in cardiogenesis (Zhang *et al*, 2005; Afouda *et al*, 2018). In mice, *Sox17* has been shown to play a critical role in endocardial differentiation during early heart development and coronary artery formation (Saba *et al*, 2019; González-Hernández *et al*, 2020). This body of work suggests that the SOXF transcription factors share an important role in controlling the differentiation of blood vascular endothelial cells and cells of the cardiac endothelium.

Constitutive inactivation of *Sox7* in the mouse results in pericardial edema and embryonic lethality, suggesting a potential role for SOX7 protein in early heart development (Wat *et al*, 2012). Some studies in embryoid bodies have implicated SOX7 as a regulatory switch between cardiac and endothelial lineages during cardiac mesoderm differentiation, through the positive regulation of Wnt and Bmp signaling (Nelson *et al*, 2009; Doyle *et al*, 2019). Further recent studies have established a role for *Sox7* in endocardial cushion formation and the modulation of atrioventricular septum formation via the modulation of the Wnt4/Bmp2 signaling pathways (Hong *et al*, 2021). In humans, *SOX7* variants have been also reported in patients displaying conotruncal defects, with some *in vitro* evidence suggesting that direct transcriptional regulation of *VE Cadherin* by this TF is essential for the endothelial-to-mesenchymal transition that takes place in the outflow tract formation during development (Jiang *et al*, 2021). Despite the clear association of *Sox7* gene function and cardiac development, it remains elusive how *Sox7*-dependent pathways maintain endothelial cell identity in cardiac vascular beds, and whether *Sox7*-positive endothelial cells instruct morphogenic events that underpin heart tissue organization.

Here, we assessed the role of SOX7 molecular role in committed cardiac endothelial cells during heart morphogenesis. Our data suggest that *Sox7* acts as a central regulator of cardiac endothelial cell differentiation and lineage maintenance and that this function is critical for governing endothelial-to-cardiomyocyte interactions, which in turn governs ventricular myocardium trabeculation and compaction. These findings identify *Sox7* as a novel endothelial-specific master regulator of mammalian cardiogenesis.

Results

Sox7 is expressed by the endocardium and is indispensable for cardiac development

To investigate the role of *Sox7* in heart morphogenesis, we first examined the expression of the SOX7 protein during development. It has been reported that all members of the SOXF family are expressed in coronary vessels but are differentially expressed in the endocardium (González-Hernández *et al*, 2020). To detect endogenous SOX7 expression, we used the *Sox7-V5*-tagged transgenic reporter mice (Chiang *et al*, 2023). At E11.5, SOX7-positive signals (white) were found exclusively in endomucin-positive endocardial cells (green), but could not be detected in the tropomyosin-positive myocardium (red) (Fig 1A). SOX7 was also observed at the outer lining of the endocardial cushion (ECC) (Fig 1A). We also investigated expression of SOX17 by immunofluorescence analysis on tissue sections, using a SOX17 antibody (Fig 1B). Interestingly, it has been previously reported that *Sox17* is critical for endocardial differentiation in endocardium precursor cells (Saba *et al*, 2019). SOX17 protein was virtually undetectable in the endocardial lining by E11.5 (Fig 1B). Our data are consistent with work from Sharma *et al* (2017), showing that *Sox17* is a coronary vessel marker not expressed in the bulk of the endocardium under physiological conditions, but is induced in a subset of activated endocardium to form coronary vessels when the sinus venosus-derived coronary growth is stunted. In contrast, SOX17 protein is highly expressed in the dorsal aorta (Fig 1B) (Corada *et al*, 2013).

Constitutive inactivation of *Sox7* in mouse embryos resulted in developmental delay, pericardial edema, and lethality as early as E10.5, consistent with a previous report (Fig 1C) (Wat *et al*, 2012). The onset of pericardial edema occurs around E9.5, prior to the general gross phenotype (Fig 1D). However, the early lethality found in constitutive *Sox7* mutant embryos prevented any further investigation on the role of *Sox7* at later stages of heart development.

Loss of *Sox7* gene function in endothelial cells during early organogenesis causes non-compaction defects in the ventricular chambers

Given the early lethality of *Sox7* constitutive knockout embryos, we used an endothelial-specific, *Cdh5-CreERT2*-inducible system to

 © 2023 The Authors

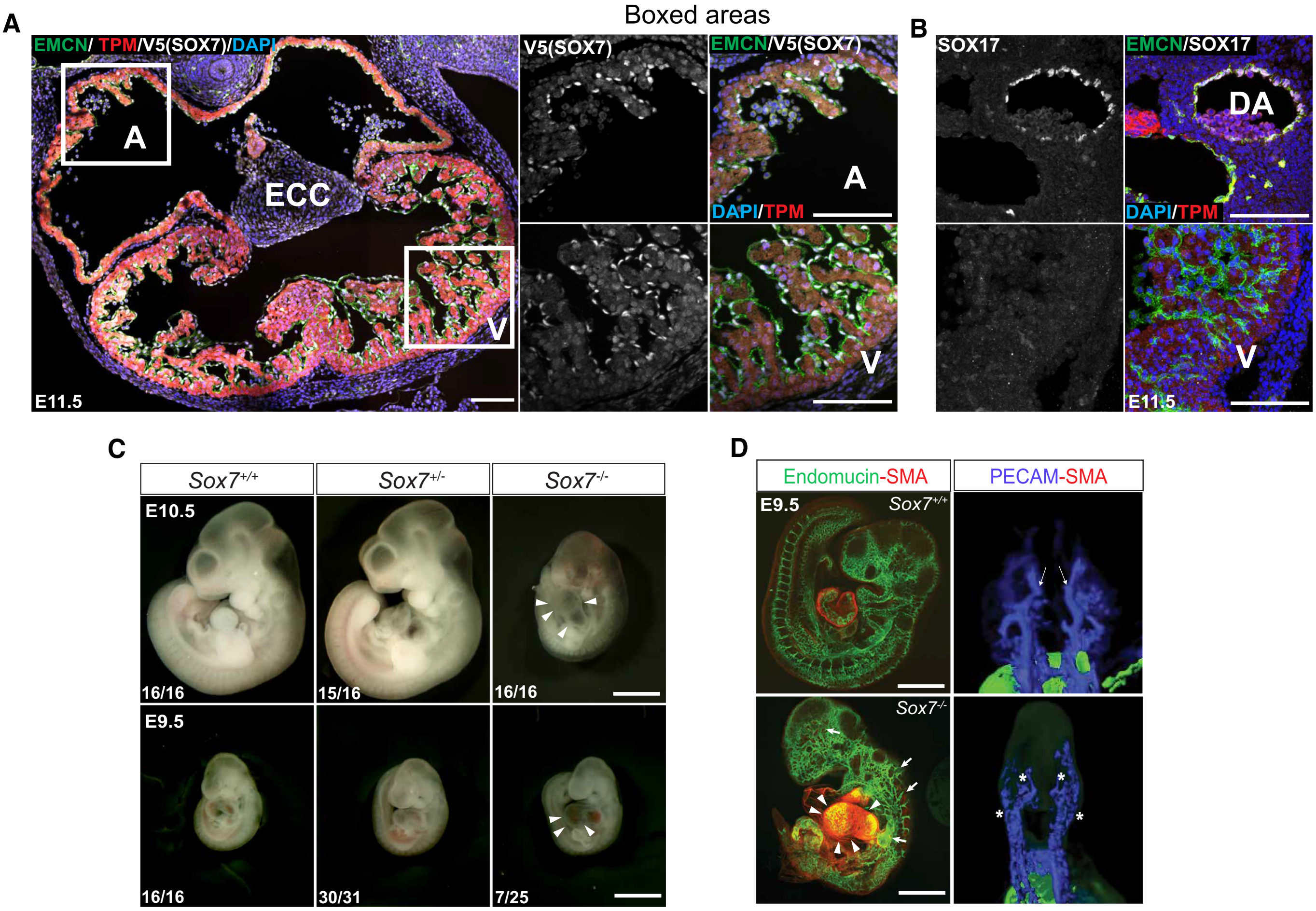

Figure 1. *Sox7* is expressed in cardiac endothelial cells and is necessary for heart development.

A SOX7 expression in the E11.5 heart based on the *Sox7-V5*-tagged transgenic reporter mice. SOX7 (white) is expressed in the endothelial lining (delineated by endomucin (EMCN), green) of the atrium (A), ventricle (V), and endocardial cushions (ECC) but not in the myocardium (marked by tropomyosin (TPM), red).

B Unlike SOX7, SOX17 (white) is not expressed in the endocardium at E11.5 (lower panels), although is expressed in the dorsal aorta (DA, upper panels).

C, D *Sox7*-null embryos show cardiac and vascular defects at E9.5 and developmental defects at E10.5 (white arrowheads). The number of embryos showing the illustrated phenotype among the total examined is indicated. Whole-mount fluorescence and confocal analysis of $Sox7^{-/-}$ mutant embryos at E9.5, with the pan-endothelial marker, endomucin (green), and the pericyte-marker, smooth muscle actin (red) reveals pericardial edema (white arrowheads) and blood vasculature defects (white arrows). Optical projection tomography (OPT) analysis using the pan-endothelial marker PECAM (blue) shows a lack of large arteries in the head vasculature (asterisks) in $Sox7^{-/-}$ mutants.

Data information: Scale bars = 100 μm (A, B), 1 mm (C), and 500 μm (D).

generate *Cdh5-CreERT2:Sox7*$^{fl/fl}$ mice (*Sox7*^{iECKO}). To minimize confounding defects from impaired vascular development shown in Fig 1D (Kim *et al*, 2016; Lilly *et al*, 2017), tamoxifen-induced excision of *Sox7* exon 2 was performed at E9.5 and E10.5, corresponding to early to mid-organogenesis. In particular, this time point corresponds to the extension phase of trabecular myocardium and therefore, *Sox7*^{iECKO} embryos will have normal establishment of trabecular units, allowing investigation of the role of endocardial *Sox7* in the later stages of trabecular myocardium development (Del Monte-Nieto *et al*, 2018). In addition, this time point is prior to the formation of coronary vessels; therefore, allowing the assessment of the role of endothelial *Sox7* during coronary vessel formation (Virágh & Challice, 1981).

To characterize the cardiac phenotype of *Sox7*^{iECKO} mutants, we performed a time-course series of morphological analyses and morphometric quantifications in order to address not only general heart morphology but also differences in the relative area occupied by trabecular, compact, and total myocardium between *Sox7*^{iECKO}mutant embryos and control littermates (Figs 2 and EV1). No morphological defects or area differences in trabecular, compact, and total myocardium in either ventricle were found at E11.5 and E12.5 in the *Sox7*^{iECKO} mutants compared to control embryos (Fig EV1A–L). However, a statistically non-significant trend toward an increase in trabecular myocardium area was found in the left ventricle of *Sox7*^{iECKO} mutant hearts compared to the control littermates at E12.5 (Fig EV1L). This increase in trabecular myocardium area was statistically significant by E13.5 in both left and right ventricles, suggesting that loss of *Sox7* in the endocardium promotes a hyper-trabeculation phenotype (Fig EV1M–R). Similar analysis at E14.5 and E16.5 confirmed the hyper-trabeculation phenotype in

© 2023 The Authors

Sox7^{iECKO} mutants and identified a significant reduction in the compact myocardium area in both ventricles (Figs 2A–F and EV1S–X). These results suggest that loss of endothelial *Sox7* promotes trabecular myocardium defects from E12.5, resulting in a non-compaction phenotype later in development.

Hyper-trabeculation defects in *Sox7*^{iECKO} mutants are associated with abnormal allocation of chamber cardiomyocytes to the trabecular layer

The hyper-trabeculation phenotype identified in *Sox7*^{iECKO} mutants could be caused by multiple factors including increased size of trabecular cardiomyocytes (hypertrophy) or increased number of cells forming the trabecular myocardium. The latter could be due either to an increase in the number of cells entering the trabecular layer from the highly proliferative compact layer (de Boer *et al*, 2012), or an increase in cardiomyocyte proliferation in the trabecular layer. To determine the cause for the increased area of the trabecular layer found in *Sox7*^{iECKO} mutants, we performed quantification of cell number and proliferation rate in the ventricular chamber myocardium, in both the trabecular and compact myocardium (Fig 2G–J, Appendix Fig S1A–E, I, J, N, O, S and T). Tissue-specific staining for endocardium (CD31) and myocardium (MF-20 or SMA) were used to generate myocardial and endocardial masks to classify the identity of cell nuclei forming the chambers. Further semiautomatic segmentation allowed the classification of the nuclei into trabecular and compact myocardial nuclei. This allowed the quantification of the number of cells forming each myocardial subregion. Once all nuclei were classified and counted, we performed analysis of Ki67 staining inside each nucleus in order to determine the proliferation rate in each myocardial subregion.

Quantification of cell number in both ventricular chambers revealed no significant difference in the total number of cells (endocardium and myocardium) forming either ventricular chamber in mutant versus control hearts. Likewise, separate quantification of the ventricular myocardium revealed no significant difference at any of the stages analyzed, although there is a trend toward an increase in the number of cardiomyocytes forming both chambers at E13.5 and E14.5 (Appendix Fig S1D, E, I and J). However, similar quantification following separation of cells forming either the compact or the trabecular myocardium identified a significant reduction in the number of cardiomyocytes forming the compact myocardium of both ventricles in *Sox7*^{iECKO} mutants at E14.5 (Fig 2G and H, left panels). Concomitantly, there was a significant increase in the number of cardiomyocytes forming the trabecular myocardium of *Sox7*^{iECKO} mutants in the left ventricle at E13.5 with an increase (non-significant) in the right ventricle that becomes significant in both ventricles at E14.5 (Fig 2G and H, right panels). These results suggest that the reduction in compact myocardium area and increase in the trabecular myocardium area observed morphologically are due to changes in the number of cells forming the compact and trabecular myocardium of *Sox7*^{iECKO} mutants, and not to cell hypertrophy.

To identify the mechanism underlying the change in cell number observed, we next analyzed cell proliferation in the *Sox7*^{iECKO} mutants. General proliferation rates in the ventricular chamber (endocardium and myocardium) and in the chamber myocardium specifically were not significantly different at E12.5 and E13.5 (Appendix Fig S1N, O, S and T). However, there was a significant reduction in cell proliferation rates in the cardiac chamber in the left ventricle and the chamber myocardium of both ventricles at E14.5 (Appendix Fig S1N, O, S and T). Similar analysis on separated compact and trabecular myocardial cells identified normal proliferation rates in both tissues at E12.5 and E13.5, but a significantly reduced rate of cardiomyocyte proliferation both in the compact and trabecular myocardium of both ventricles of *Sox7*^{iECKO} mutants at E14.5 (Fig 2I and J). These results suggest that the increase in cardiomyocyte number observed in the trabecular myocardium is not a result of a local hyper-proliferative response. Since we observed decreased proliferation in both compartments (trabecular and compact), this cannot explain the differential increase in cell number in the trabecular layer and decrease in the compact zone. This elevated number of cells in the trabecular layer is most likely due to an increase in the allocation of cardiomyocytes from the compact layer into the trabecular layer, hence resulting in the reduction in compact myocardium cells (Fig 2G–J).

Endothelial-specific loss of SOX7 function perturbs coronary artery formation

To further investigate the generalized reduction in cell proliferation we observed in both the trabecular and compact layers at E14.5, we turned toward a possible vascular phenotype we noted while characterizing the cardiac tissue architecture. We observed a dramatic reduction in the number of coronary vessels in both the subepicardial space (coronary veins) and within the compact myocardium (coronary arteries), suggesting that the loss of endothelial *Sox7* function may also cause defects in coronary vessel formation (Figs 2C and EV1U; empty arrows). The significant reduction in cell proliferation in the trabecular and compact layers at E14.5 may be related to the lack of proper nourishment and oxygenation of the fast-growing myocardium due to defects in the formation of the coronary plexus found in *Sox7*^{iECKO} mutants. The coronary vasculature starts to form very early in development, but it only connects to the blood flow at around E13.5 when the coronary plexus connects to the base of the aorta (Virágh & Challice, 1981). Therefore, defects in the proper formation of the coronary vascular plexus may explain the cardiac defects found in *Sox7*^{iECKO} mutants after E13.5. This observation prompted us to investigate the mechanism underlying the vascular defects in the *Sox7*^{iECKO} mutants, and we next set out to analyze the coronary plexus.

To further investigate the coronary vessel defects identified during the morphological characterization of *Sox7*^{iECKO} mutant hearts (Figs 2C and EV1U, empty arrows), we studied the patterning of the coronary artery plexus. We performed whole heart immunofluorescence staining using antibodies against the arterial marker, CX40, and a general endothelial marker, VEGFR2, to visualize the coronary arteries and coronary vessel plexus, respectively (Fig 3A). In *Sox7*^{iECKO} mutant hearts, we consistently failed to detect distal coronary arteries (marked by arterial marker CX40 in red), while formation of the coronary stems appeared intact (Fig 3A, Appendix Fig S2, arrows). In contrast, the surrounding coronary vessels, labeled with VEGFR2 and Histone H3, appeared grossly normal (Fig 3A and B, Appendix Fig S2). The seemingly normal vessel network suggests that sprouting angiogenesis is not compromised in the cardiac tissue of *Sox7*^{iECKO} embryos. We confirmed this by quantifying the

 © 2023 The Authors

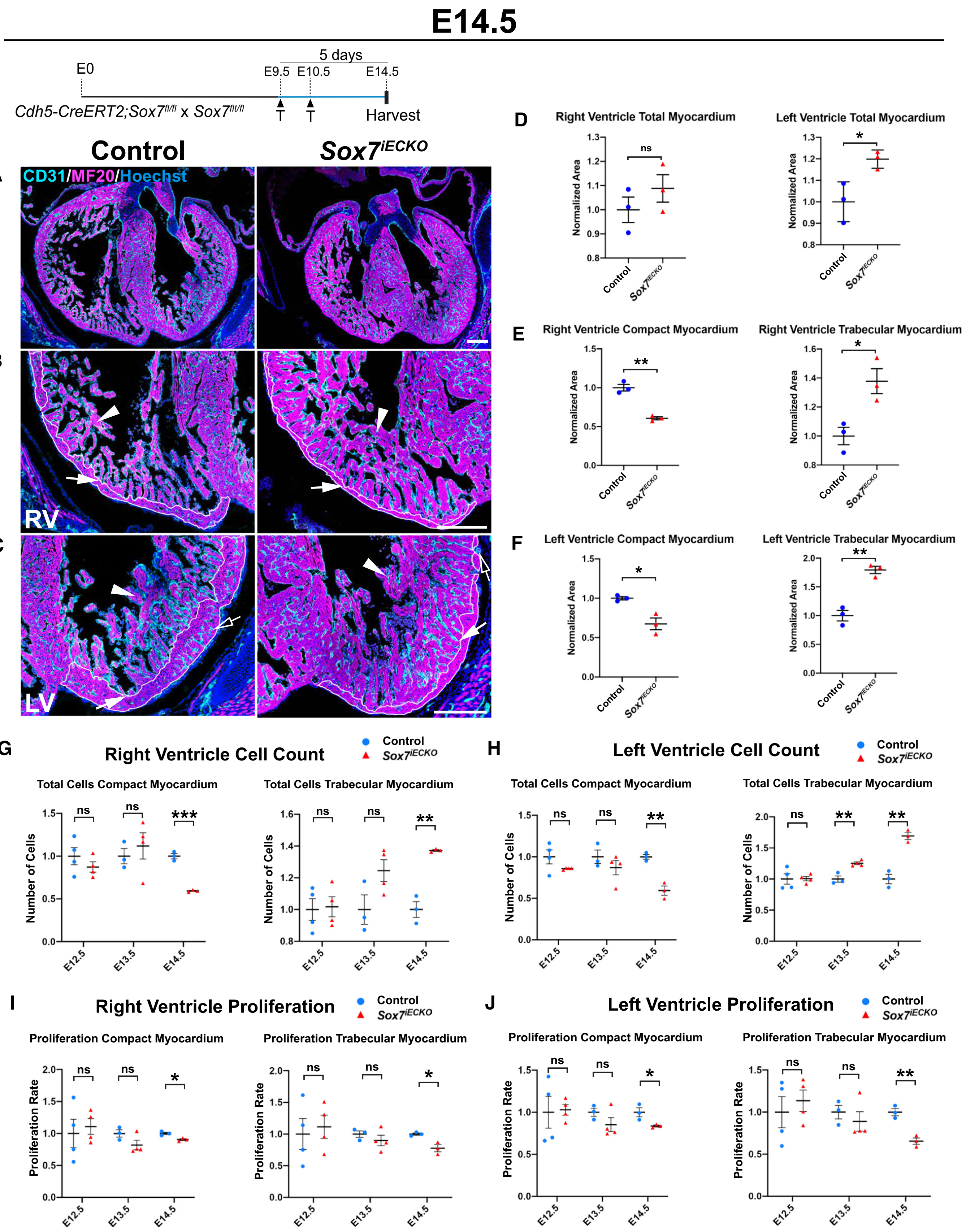
E14.5
5 days
E0
E9.5 E10.5 E14.5
Cdh5-CreERT2;Sox7fl/fl x Sox7fl/fl
T T
Harvest
Control
Sox7iECKO
A
CD31/MF20/Hoechst
B
RV
C
LV
D
Right Ventricle Total Myocardium
Left Ventricle Total Myocardium
Normalized Area
E
Right Ventricle Compact Myocardium
Right Ventricle Trabecular Myocardium
F
Left Ventricle Compact Myocardium
Left Ventricle Trabecular Myocardium
G
Right Ventricle Cell Count
Total Cells Compact Myocardium
Total Cells Trabecular Myocardium
Number of Cells
H
Left Ventricle Cell Count
I
Right Ventricle Proliferation
Proliferation Compact Myocardium
Proliferation Trabecular Myocardium
Proliferation Rate
J
Left Ventricle Proliferation
E12.5
E13.5
E14.5

]Figure 2.

Figure 2. Endothelial-specific loss of *Sox7* function leads to non-compaction cardiac defects.

A–C Morphological characterization of the cardiac defects in *Sox7*^{iECKO} hearts compared to littermate controls at E14.5 after Cre induction by tamoxifen injection at E9.5 and E10.5, by immunofluorescence analysis with tissue-specific markers CD31 (endocardium/endothelium; cyan), MF20 (myocardium; magenta), and nuclei (Hoechst; blue). (A) Low-magnification images showing the general heart morphology. (B, C) High-magnification images showing the morphology of the compact (arrows) and trabecular (arrowheads) myocardium, and coronary arteries (empty arrow) in the right (RV; B) and left (LV; C) ventricles. Note the clear increase in trabecular myocardium (arrowheads), reduction of compact myocardium (arrows, white contour), and the absence of intramyocardial coronary arteries (empty arrow) in *Sox7*^{iECKO} hearts compared to control hearts.

D–F Quantification of the area occupied by total, compact, and trabecular myocardium in each ventricular chamber, showing a statistically significant increase in total (D) and trabecular (E, F, right graphs) myocardium area, and a statistically significant decrease in compact myocardium area (E, F, left graphs) in *Sox7*^{iECKO} hearts compared to control hearts.

G, H Quantification of the number of cardiomyocytes forming the compact (left graphs) and trabecular (right graph) myocardium of the right (G) and left (H) ventricles in *Sox7*^{iECKO} hearts compared to control hearts at E12.5, E13.5, and E14.5. Cell counts confirmed that the increase in trabecular myocardium and decrease in compact myocardium areas in *Sox7*^{iECKO} hearts is due to an increase and decrease in cardiomyocytes in the trabecular and compact myocardium, respectively.

I, J Quantification of the number of Ki67^{+}-proliferating cardiomyocytes compared to the total number of cardiomyocytes in the compact (left graphs) and trabecular (right graph) myocardium of the right (I) and left (J) ventricles in *Sox7*^{iECKO} hearts compared to control hearts at E12.5, E13.5, and E14.5. A significant reduction in cardiomyocyte proliferation in both the compact and trabecular myocardium was observed in *Sox7*^{iECKO} hearts compared to control hearts at E14.5.

Data information: Biological replicates as per the number of data points in the graphs. Mean ± SEM; *t*-test. $P < 0.05$ (*); $P < 0.005$ (**); $P < 0.0005$ (***); ns = not significant. Scale bars = 300 μm (A), 200 μm (B, C).

coronary vessel coverage, ERG^{+} endothelial cell number, and their proliferation rate in hearts from mutant and littermate control E13.5 embryos (Fig 3C–E). These results suggest an important role for *Sox7* in the coronary endothelium in the process of distal coronary artery assembly, but not coronary plexus formation. These results were further supported by morphometric analysis of the epicardial and intramyocardial coronary vessels (Appendix Fig S3). This analysis was done at E14.5 comparing *Sox7*^{iECKO} to control embryonic hearts and identified a significant reduction in intramyocardial vessels, subepicardial vessels, and subepicardial area. The results suggest that coronary and subepicardial defects are associated with the loss of *Sox7* function in the endothelium (Appendix Fig S3A and B). However, all these changes were not maintained at E16.5 (Appendix Fig S3C and D), suggesting that the reduction in these parameters at E14.5 may be due to a delay in epicardium, subepicardium, and coronary vessel formation most likely due to the absence of proper growth of the compact layer. Indeed, at E16.5, the compact myocardium of *Sox7*^{iECKO} hearts is perfused by coronary vessels and both the subepicardium and its vessels look normal (Appendix Fig S3C). Interestingly, no major coronary vessels were present inside the compact myocardium of *Sox7*^{iECKO} hearts compared to control (Appendix Fig S3C, arrow), suggesting that *Sox7* may play a role in the maturation and hierarchization of coronary vessels.

To determine whether the loss of distal coronary arteries observed in the *Sox7*^{iECKO} is a cardiac-specific defect or a more general vascular defect, we analyzed the process of blood vessel formation at earlier stages in constitutive *Sox7*$^{-/-}$ embryos. Detailed analysis of vibratome sections of *Sox7*$^{-/-}$ embryos as early as 17–18 somite stage (ss) identified arterio-venous shunts between the dorsal aorta (DA) and the cardinal vein (CV) (Figs 3F and EV2A). This type of vascular by-pass is a classic phenotype of compromised arterio-venous identity, suggesting that the loss of distal coronary arteries is likely due to the loss of arterial identity, akin to the phenotypic outcome in the *Sox17* loss of function scenario (Corada *et al*, 2013; González-Hernández *et al*, 2020).

SOX7 directly regulates early markers of future coronary arteries

To investigate putative downstream targets of SOX7 TF involved in arterial specification, we performed bulk RNA-Seq analysis on whole *Sox7* knockout embryos at E8.5 before any traces of gross severe vascular defects (Appendix Fig S4A and B). This approach revealed 15 down- and 50 up-regulated genes. Gene ontology analysis revealed that the differentially expressed genes have been primarily implicated in blood vessel development and angiogenesis. Of these genes, *Dll4*, *Cx37*, and *Cx40* have a documented role in arterial specification (Benedito *et al*, 2008; Gkatzis *et al*, 2016; Fang *et al*, 2017), with *Dll4* being a known target directly regulated by SOX7 (Sacilotto *et al*, 2013). Furthermore, *Cx40* has been shown to mark the "pre-artery" cells that build coronary arteries (Su *et al*, 2018). To further confirm SOX7-dependent cell-autonomous regulation of these genes in the endothelium, we performed RNA-Seq analysis of CD31^{+} cells sorted from *Sox7*^{iECKO} and sibling control embryos at E10.5 from dams pulsed with tamoxifen at E9.5. Of the handful of differentially expressed genes, we repeatedly observed the downregulation of *Cx40* (Appendix Fig S4C). We confirmed the reduction of *Cx40* transcript levels in sorted endothelial cells from *Sox7*^{iECKO} and sibling controls by qPCR (Fig 4A). In addition, we also validated the expression levels of a closely related gene *Cx37*, identified as dysregulated in the whole-embryo RNA-Seq analysis. To further assess the regulation of *Cx37* and *Cx40* by SOX7 in an arterial specified cell line, we performed gene knockdown of *SOX7* using a siRNA-based approach in the human arterial line HUAECs. This led to a reduction in *CX37*, *CX40*, and *DLL4* expression levels, while expression of endothelial markers such as *ERG* and *VEGFR2* remained unchanged (Fig 4B). This series of *in vitro* and *in vivo* experiments position both *Cx37* and *Cx40* as downstream effectors of the *Sox7* pathway in endothelial cells.

To further validate the positive regulation of *Cx37* and *Cx40* by SOX7 *in vivo*, we next assessed *Cx37* transcript levels in *Sox7*$^{-/-}$ embryos by whole mount *in situ* hybridization at E8.5 (Fig 4C). In the constitutive knockout, loss of *Sox7* function was validated at the phenotypic level by the presence of arterio-venous shunts (Figs 3F and EV2A). In these experiments, *Cx37* expression appeared reduced in the mutant dorsal aorta at E8.5. SOX7-dependent downregulation of CX40 at the protein level was revealed by immunofluorescence analysis on tissue sections or whole-skin staining from E11.5 and E14.5 (Figs 4D and EV2B). Furthermore, *Cx40* transcript levels were downregulated specifically in the ventricular coronary vessels, as shown by *in situ* hybridization on sections of *Sox7*^{iECKO} mutant hearts at E14.5 (Fig 4E).

 © 2023 The Authors

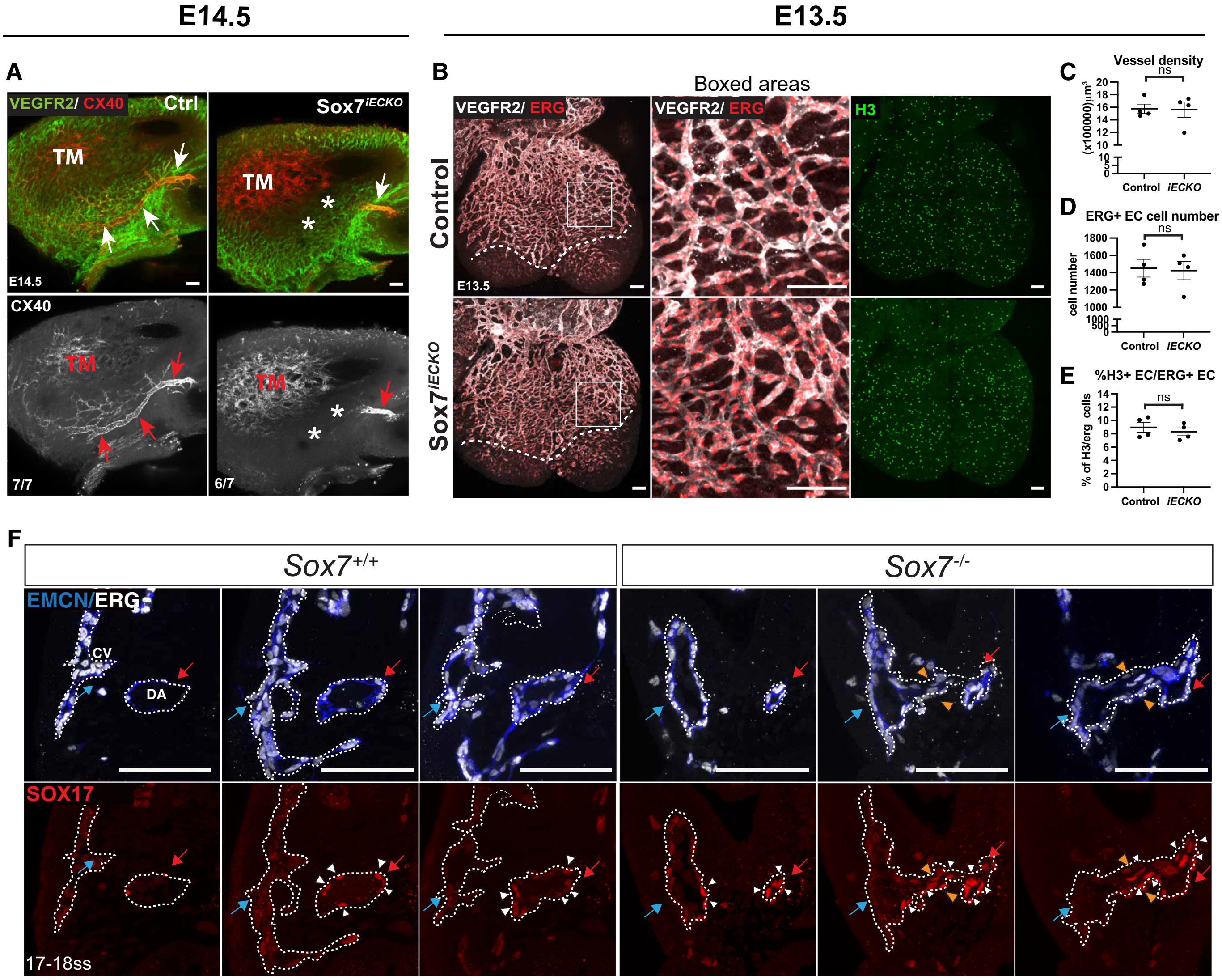

Figure 3. Specification of the coronary artery and dorsal aorta is dependent on endothelial *Sox7* function.

A Whole-mount immunostaining of control and $Sox7^{iECKO}$ hearts at E14.5 after Cre induction by tamoxifen injection at E9.5, E10.5. Coronary vessels are stained with VEGFR2 (green), coronary arteries (arrows) and trabecular myocardium (TM) are both stained with CX40 (red). Asterisks show where the major distal coronary arteries are lost in the $Sox7^{iECKO}$ heart. The number of embryos showing the illustrated phenotype among the total examined is indicated.

B Whole-mount immunostaining of control and $Sox7^{iECKO}$ hearts at E13.5 after Cre induction at E9.5 and E10.5. Coronary vessels are stained with VEGFR2 (white) and endothelial nuclei with ERG (red). The coronary plexus front is outlined by the dotted lines.

C–E Graphs showing quantification of coronary vessel density (C), ERG-positive endothelial cells (D), and % of $H3^+$ proliferative endothelial cells (E) in the coronary plexus. Scored sibling control, $n = 4$; $Sox7^{iECKO}$ mutant embryos, $n = 4$ embryos; Mean ± SEM; Mann–Whitney *U*-test. ns = not significant.

F Serial sections of a wild-type and a $Sox7^{-/-}$ embryo at 17–18 ss (E9.0), stained with endomucin (blue) to detect the endothelial lining, ERG (white) for endothelial nuclei, and SOX17 (red) for arterial nuclei. The dorsal aorta (DA) is labeled by the red arrows and white arrowheads, while cardinal vein (CV), blue arrows. Fusion between DA and CV is indicated by orange arrowheads.

Data information: Scale bars = 100 μm.

To examine if *Cx40* is a potential direct target gene of SOX7, we analyzed the SOX7-mCherrry ChIP-Seq dataset available from the human venous endothelial cell line (HUVECs) (https://www.ebi.ac.uk/arrayexpress/experiments/E-MTAB-4480/). This approach was designed to uncover putative SOX7-dependent regulatory elements associated with the *CX40* gene. The most common binding motif identified in the SOX7 ChIP-Seq dataset corresponds to the reported SOX motif 5′-A/TTTGTT-3′ (Fig EV2C). From this dataset, we identified a 500 bp putative CX40 regulatory element situated 21 kb upstream from the transcription start site (*CX40-21*). The *CX40-21* site appears to coincide with an open chromatin region (revealed by DNAseI footprint, black peaks) associated with the binding of active histone marker, H3K27Ac (light blue), and strong enhancers (ChroMM, orange bar) (EVD). To test the activity of this DNA element *in vivo*, we cloned a fragment of this region into the ZED enhancer reporter system (Bessa *et al*, 2009) and generated stable transgenic reporter zebrafish lines. The ZED vector has a minimal gata2 promoter driving GFP expression in the presence of the

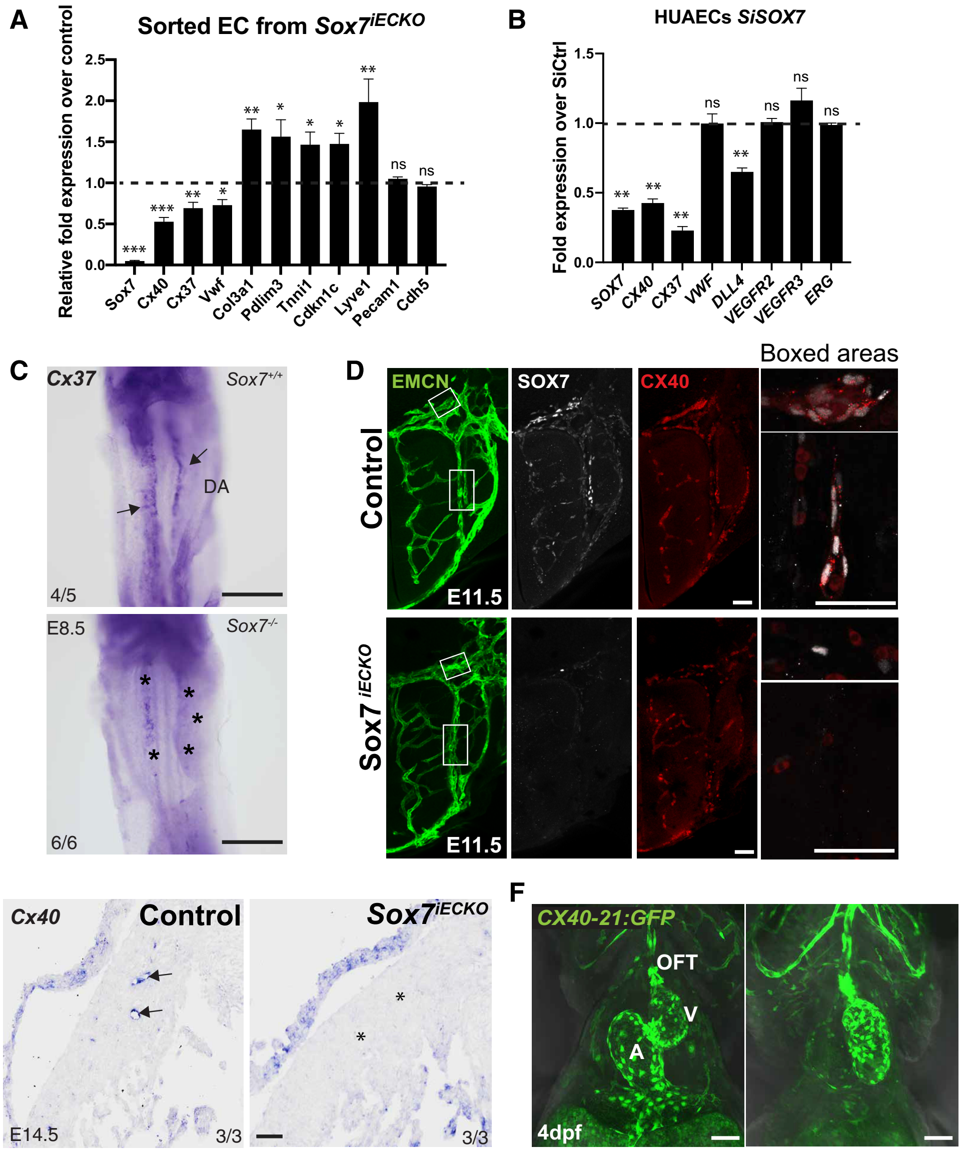

Figure 4. *Sox7* modulates arterial specification through transcriptional regulation of *Connexin 37* and *40*.

A Quantitative PCR analysis on FACs-sorted PECAM$^+$CD45$^-$ endothelial cells from *Sox7*^{iECKO} mutants and sibling controls at E10.5, injected with tamoxifen at E9.5. Expression is normalized to the endothelial marker *Pecam* and *Cdh5*. Scored sibling controls, n = 7 embryos; *Sox7*^{iECKO} mutants, n = 7 embryos. The dashed line refers to the expression level of gene expression in control samples and is arbitrarily set to 1.

B Quantitative PCR analysis on the human arterial endothelial cell line (HUAECS) transfected with *SiSOX7* or *SiCTRL* for 48 h. Expression is relative to *HPRT* and *GAPDH*. Biological replicates, n = 6. The dashed line refers to the expression level of gene expression in control samples and is arbitrarily set to 1.

C Representative images of *Cx37 in situ* hybridization on whole E8.5 (8–10 ss) embryos. The staining in *Sox7*$^{-/-}$ dorsal aortae is markedly reduced (asterisks) when compared with the wild-type controls (black arrows).

D Section immunostaining of control and *Sox7*^{iECKO} early neuronal vascular plexus at E11.5. Blood vessels are stained with endomucin (green); SOX7 (white); and CX40 (red). Scale bars = 40 μm.

E Representative images of *Cx40* section *in situ* hybridization on E14.5 hearts. The staining in *Sox7*^{iECKO} coronary vessel in the ventricle free wall is markedly reduced (asterisks) when compared with the controls (black arrows). The number of embryos showing the illustrated phenotype among the total examined is indicated. Scale bar = 75 μm.

F The *CX40-21:GFP* transgene directs GFP fluorescence expression to the heart endothelium and vascular endothelium, in transgenic zebrafish larvae at 4 dpf. OFT, outflow tract; A, atrium; V, ventricle.

Data information: Scale bars = 50 μm. (A, B) Mean ± SEM; *t*-test. $P < 0.05$ (*); $P < 0.005$ (**); $P < 0.0005$ (***).

CX40-21 region and enabled us to assess enhancer activity of this regulatory element during development. As shown in Figs 4F and EV2E, the 500 bp *CX40-21* DNA element has the ability to drive GFP expression into cardiac endothelium and blood vessels in zebrafish larvae (Figs 4F and EV2E). Altogether, these data indicate that SOX7 plays an indispensable role in arterial specification of the distal

    © 2023 The Authors

coronary artery through direct transcriptional regulation of *Cx40* and *Cx37*.

Endothelial-specific deletion of *Sox7* depletes a subpopulation of endocardial cells during heart development

While it is evident that SOX7 plays a pivotal role in arterial specification of the coronary artery, it is unlikely that the hypertrabeculation and non-compaction phenotype described above is a result of coronary artery malformation. The myocardium phenotype is initiated as early as E12.5 before coronary arteries are formed. To further dissect the molecular function of SOX7 in cardiac endothelial cells and its role in shaping the transcriptomic profiles of cardiac cell populations, we performed single-nuclei (sn) RNA-Seq on whole hearts from *Sox7*^{iECKO} and sibling control embryos at E12.5 3 days after Cre-mediated excision by tamoxifen treatment (Fig 5A). The gene-targeted disruption was confirmed by the presence of *Sox7* transcripts missing exon 2 (the exon flanked by the loxP sequences) in the *Sox7* locus of the *Sox7*^{iECKO} hearts (red track) (Fig EV3A).

snRNA-Seq analysis was performed using the Chromium system (10× Genomics), with individual nuclei collected from a pool of four hearts per genotype. A total of 6,414 control and 5,986 *Sox7*^{iECKO} nuclei were used for subsequent downstream analysis. Using the *t*-distributed stochastic neighbor embedding (*t*-SNE) approach, we identified a total of 13 distinct cell clusters, annotated based on the expression of top differentially expressed and known marker genes. These include three cardiomyocyte clusters (*Ttn*, *Myh6*, *Nppa*), three cardiac endothelial clusters (*Flt1*, *Pecam1*, *Fli1*), three hematopoietic cell clusters (*Hba-a2*, *Itga2b*, *Ly86*), two fibroblast clusters (*Ptn*, *Pdgfra*), one epicardial cluster (*Wt1*, *Tbx18*), and one cluster corresponding to cardiac valvular cells (*Vcan*, *Tbx20*) (Figs 5B–D and EV3B–E). Interestingly, all cell clusters were represented in the hearts of embryos of both genotypes, although their relative proportions varied between mutant and control samples.

It has been reported that the cardiac endothelial cell population is the most enriched population in the adult mouse heart (Pinto *et al*, 2016). Strikingly, general analysis of the relative contribution of the different cell populations in *Sox7*^{iECKO} mutants compared to control embryos identified a redistribution in the relative proportions of different cell types in *Sox7*^{iECKO} mutant hearts, with cardiomyocytes being the most abundant cell type outnumbering the diminishing endothelial cell population in this genotype (Figs 5E and F, and EV3B). We next interrogated the dataset to further investigate the molecular signatures contributing to this shift in the relative proportion of each cell population contributing to the *Sox7*^{iECKO} mutant heart.

The vast majority of endothelial cells have an endocardial cell identity as shown by C2, C5, and C7 clusters that express the *Npr3* marker (Fig 5D) (Zhang *et al*, 2016). This is consistent with the fact that the coronary vessels originating from the sinus venosus and endocardium have just started to infiltrate the cardiac tissue at E11.5 (Red-Horse *et al*, 2010). Of the three cardiac endothelial clusters, C5 is not proliferative as shown by the low levels of *Daiph3*, *Ki67*, and *Ttk* expression (Fig EV3C). A small subpopulation of endothelial cells expressed coronary vessel markers such as *Fabp4 and Apelin* (*Npr3* low) within the proliferative population C7 (Fig 5D, red arrowheads) (Red-Horse *et al*, 2010; He *et al*, 2014). Interestingly, we found a dramatic reduction in the C2 cardiac endothelial cluster in the *Sox7*^{iECKO} sample, while the other two clusters (C5, C7) remained unchanged (Fig 5B, E and F). Further analysis of the C2 endocardial population reveals that these cells display higher expression levels of genes such as *Sox9* and *Pdgfra* that define endovascular progenitor cells (Fig EV3C) (Patel *et al*, 2017). In addition, cells in the C2 cluster also express *Gpc3*, a prominent liver stem cell marker (Grozdanov *et al*, 2006; Su *et al*, 2017), suggesting that the C2 population might represent a multipotent cell state. Unlike C5 and C7, heatmap analysis showing the expression profile of the C2 cell population reveals a blend of signals common to other populations (Fig 5C), suggesting that C2 represents a more immature endocardial population compared to C5 and C7.

To validate the presence of the C2 population *in vivo*, we analyzed the co-expression of *Sox7*, *Sox9*, and *Gpc3* in the heart of E12.5 *Sox7-V5* transgenic reporter embryos. Gene co-expression analysis of the C2 cluster shows that around 6% of the endocardial progenitor cells are triple positive for these markers. smFISH for *Gpc3* and *Sox9* combined with V5 immunofluorescence confirms the presence of a subset of the trabecular endocardium that is triple positive for SOX7, *Sox9*, and *Gpc3* (Fig EV4A, orange arrows). Consistent with what we observed in the snRNA-Seq analysis when comparing control and *Sox7*^{iECKO}, we observed a general decrease in *Gpc3*- and *Sox9*-positive cells in the mutant condition. This is especially evident in the right ventricle endocardium, but not as pronounced in the left ventricle (Fig EV4B). This observation confirms that a subset of the C2 population is lacking expression of genes with stemness potential during *Sox7* loss of function.

To further address possible changes in the endocardial composition in *Sox7*^{iECKO} mutant hearts stemming from the diminished C2 endocardial cell population, we quantified cell number and proliferation rate in the endocardium as described above (Appendix Fig S1F–H, K–M, P–R and U–W). In these experiments, quantification was performed separately in both the basal and apical endocardium, which are known to display differential expression of genes and function (Del Monte-Nieto *et al*, 2018). Quantification of cell number identified no significant differences in the number of cells forming the endocardium or the basal and apical endocardium of *Sox7*^{iECKO} embryos at E12.5 and E13.5. However, there was a significant reduction in the number of endocardial cells in the right ventricle of *Sox7*^{iECKO} mutants at E14.5 with a clear decrease (non-significant) in the left ventricle (Appendix Fig S1F and K). Interestingly, this reduction in endocardial cells was significant exclusively in the basal endocardium of the right ventricle at E14.5, with no significant difference in the apical endocardium (Appendix Fig S1G, H, L and M). No statistically significant changes in proliferation were observed in mutant samples, with the exception of a significant increase in the number of proliferating cells in the apical endocardium at E13.5 and E14.5 (Appendix Fig S1P–R and U–W). However, there is a clear non-significant trend showing increased endocardial cell proliferation at all stages analyzed (Appendix Fig S1P–R and U–W).

Endothelial-specific deletion of *Sox7* promotes an increase in the specification of trabecular cardiomyocytes in the developing heart

The changes observed across the C2, C5, and C7 endothelial populations in *Sox7*^{iECKO} hearts are paralleled by an enrichment of

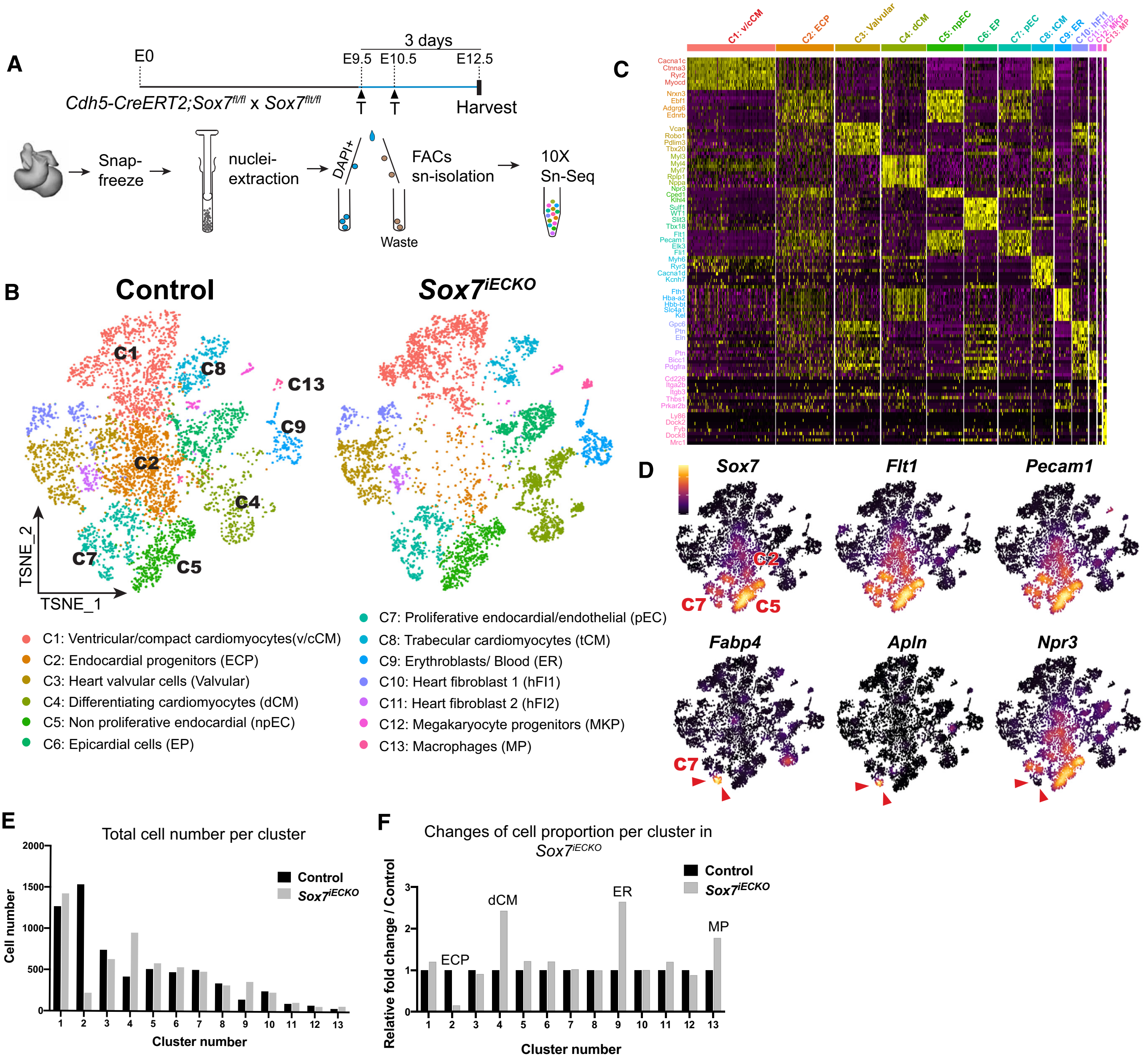

Figure 5. Single-nuclei RNA-Seq analysis of control and *Sox7*^{iECKO} hearts at E12.5 show changes in cardiac cell populations.

A E12.5 control and *Sox7*^{iECKO} hearts harvested from dams injected with tamoxifen at E9.5 and E10.5. Hearts (pooled from four hearts) were snap frozen; nuclei were isolated and sorted by FACs before loading into the 10× Chromium Genomics platform.

B, C *t*-SNE plots and unsupervised cell clustering analysis showing the top 10 differentially expressed marker genes from the 13 identified clusters. V-CM, ventricular/compact cardiomyocytes; ECP, endocardial progenitors; d.CM, differentiating cardiomyocyte; np-EC, non-proliferative endocardial; EP, epicardial cells; p-EC, proliferative endocardial/endothelial; T.CM, trabecular cardiomyocytes; FI, fibroblasts; MCP, megakaryocyte progenitors; MP, macrophages.

D Feature plots showing the different selected markers for cardiac endothelial cells (*Sox7*, *Flt1*, *Pecam1*; top), and markers for coronary vessels (*Fabp4*, *Apln*) and endocardial cells (*Npr3*) (bottom). Red arrowheads delineate the coronary vascular endothelial population.

E, F Graphs showing the cell number per cluster and changes in cell proportion, relative to the control, per cluster in E12.5 control and *Sox7*^{iECKO} hearts.

subpopulations representing cardiomyocyte (C4) and hematopoietic cell lineages (C9 and C13) (Figs 5B, E and F, and EV3B). This is consistent with the increase in cardiomyocyte populations characterized in the trabecular myocardium described above (Figs 2E–H and EV1Q, R, W and X). Unlike the other two cardiomyocyte clusters (C1 and C8), C4 shows higher expression of cardiac progenitor markers such as *Nkx2.5* and *Desmin* (Lien *et al*, 1999; Fuchs *et al*, 2016). In contrast, higher expression of genes such as *Slc8a1*, *Actn2*, *Cacna1c*, and *Ryr2* involved in electrophysiology and calcium handling suggests that C1 and C8 populations are in a more mature state (Fig EV3D) (Karbassi *et al*, 2020). In addition, C4 is enriched for expression of ribosomal proteins (*Rpl32*, *Rplp1*, *Rpl41*),

 © 2023 The Authors

suggesting that these cells actively engage with protein translation and are metabolically active (Fig EV3D). It has been shown that early differentiating cells display higher levels of ribosomal protein expression (Sampath *et al*, 2008; Ingolia *et al*, 2011; Buszczak *et al*, 2014) suggesting that C4 is undergoing active differentiation. snRNA-Seq analysis revealed in the C4 cluster a more pronounced expression level of *Nppa* and *Hopx*, two known trabecular markers. In order to further validate *in vivo* changes in the C4 cardiomyocytes subpopulation, we analyzed *Nppa* and *Hopx* gene expression pattern comparing control and *Sox7*^{iECKO} E12.5 embryos. As expected, *Hopx* and *Nppa* transcripts were found restricted to the trabecular myocardium in the ventricular chambers (Appendix Fig S5). Interestingly, whereas in control embryos, the expression of both markers is restricted toward the trabecular apex (arrows in Appendix Fig S5A–F, left panels), in *Sox7*^{iECKO} mutant hearts, we observed an expansion of these markers to the base of the trabeculae (arrows in Appendix Fig S5A–F, right panels). Lastly, in parallel to this ectopic expression of trabecular markers, we observed a significant reduction in the expression levels of three markers specific to the compact myocardium (*Kcnd2*, *Hey2*, and *Mycn*) in the C1 population (Appendix Fig S5G) when comparing control and *Sox7*^{iECKO}, consistent with the thinning of the compact myocardium (Figs 2 and EV1).

Altogether these results suggest that the loss of *Sox7* function in the endothelium compartment causes a dramatic expansion of the C4 trabecular cardiomyocytes at the expense of the C1 compact myocardium population. This result suggests that the C2 endothelial subpopulation is essential to preserve a proper ratio between the trabecular and compact myocardium cell populations.

Here, we established a correlation between the depletion of the C2 endocardial population and a parallel increase in a cardiomyocyte subpopulation undergoing differentiation in the developing ventricle of the *Sox7*^{iECKO} mutant. This suggests that the cardiomyocyte maturation process is under the influence of both the number of cardiac endocardial cells and their degree of differentiation. This observation is strengthened by analysis of the snRNA-Seq dataset using the monocle algorithm, which infers cell fate trajectory. To account for a bias caused by C2 depletion, we excluded the C2 cluster from the pseudo-time analysis. The loss of *Sox7* function in the endothelial compartment strongly influences cell state transition within the cardiomyocyte subpopulations (Appendix Fig S6A and B).

To discount the possibility that the loss of endothelial *Sox7* may lead to the aberrant transdifferentiation of the C2 endocardial cells into C4 cardiomyocytes, we performed lineage-tracing analysis of *Sox7*^{iECKO} cells labeled with the *mT/mG* reporter system (Appendix Fig S7). In this assay, the Cre activity arrests the membrane-bound Tomato expression and instead, triggers the expression of membrane-bound EGFP. Analysis of EGFP-positive cells in the *Sox7*^{iECKO} samples did not reveal the presence of any discrete pools of EGFP^{+} cardiomyocytes, suggesting that it is unlikely that the increase in the C4 cardiomyocyte population is caused by a fate shift in the endocardial progenitor population.

Endothelial-specific deletion of *Sox7* alters the balance of erythro-myeloid lineages in the developing heart

Since it is well established that *Sox7* function maintains endothelial cell identity at the expense of the hemogenic endothelium compartment in the dorsal aorta, it is possible that the lack of SOX7 function prompts C2 cells to differentiate into an erythro-myeloid lineage. Consistent with these findings, the C9 cell cluster shows expression of hemoglobin genes (*hba-a2*, *hbb-bt*) and erythroblast markers (*Ermap*, *Slc4a1*, *Nudt4*, *Tfrc*), and is proportionately over-represented in the *Sox7*^{iECKO} hearts (Figs 5B, C, E and F, and EV3E). Likewise, the C13 cell population that displays high levels of expression of genes associated with heart macrophages (*Ptprc*, *Csf1r*, and *Lair1*) is over-represented in *Sox7*^{iECKO} relative to control hearts (Figs 5C and F, and EV3E).

To validate the observed variation in the cellular composition of *Sox7*^{iECKO} hearts, we next performed fluorescence-activated cell sorting (FACS) analysis in E13.5 hearts using the pan-hematopoietic marker, CD45 (*Ptprc*), the early erythroblast marker, CD71 (*Tfrc*), and the pan-endothelial marker, CD31 (*Pecam1*) (Fig 6). To avoid contamination from circulating blood cells, all embryonic hearts were flushed with saline buffer prior to analysis (Fig 6A). Similar to the snRNA-Seq analysis where the C2 cluster is diminished in *Sox7*^{IECKO} hearts relative to controls, there was a consistent depletion of a fraction of the endothelial cell population (CD71^{-}CD45^{-}CD31^{+}) in the *Sox7*^{iECKO} hearts. This was paralleled by an increase in a hematopoietic population that expresses CD31 (CD71^{-}CD45^{low}CD31^{+}) (Fig 6B–E), whereas the CD71^{-}CD45^{low}CD31^{-} remained unchanged (Appendix Fig S8A and B). CD71 is a well-established marker of early erythroblast cells (Dong *et al*, 2011; Chao *et al*, 2015). Quantification of the CD71^{high} population also revealed a higher number of cells in this subpopulation in *Sox7*^{iECKO} hearts compared to control hearts (Fig 6F–I), thereby confirming at the experimental level, our initial observations. This suggests that loss of *Sox7* function promotes the depletion of the C2 cardiac endothelial cell population, which may in turn give rise to an increase in the erythro-myeloid lineages in the heart at around E12.5–13.5. These results are supported by the known role of *Sox7* as a gatekeeper of endothelial cell identity at the expense of the hemogenic endothelium (Gandillet *et al*, 2009; Costa *et al*, 2012; Lilly *et al*, 2016). Furthermore, the identification of the endocardium with hemogenic potential (Nakano *et al*, 2013; Zamir *et al*, 2017) suggests that SOX7 most likely plays a similar function to its role in the dorsal aorta in order to preserve a balanced distribution of endothelial and hemogenic compartments.

To investigate the possibility that the loss of endothelial *Sox7* may lead to the aberrant transdifferentiation of the C2 endocardial cells into hematopoietic cells, we performed lineage-tracing analysis of *Sox7*^{iECKO} cells labeled with the *mT/mG* reporter system (Fig EV5A and B). The principle of this assay is similar to the lineage-tracing approach performed above with the cardiomyocyte populations. Analysis of the *Sox7*^{iECKO}*; mT/mG* samples showed the presence of EGFP^{+}/CD34^{+}- or EGFP^{+}/CD45^{+}-positive cell pools in the coronary vasculature, suggesting that the increase in the C9/C13 hematopoietic populations is caused by a fate shift in the SOX7-positive endocardial progenitor population.

Paracrine signaling from endothelial cells modulates cardiomyocyte behavior *in vitro* in a *Sox7*-dependent manner

Given the marked changes in cardiomyocyte response when *Sox7* function is compromised in the endothelial cell compartment, we next set out to assess the functional consequences in

© 2023 The Authors

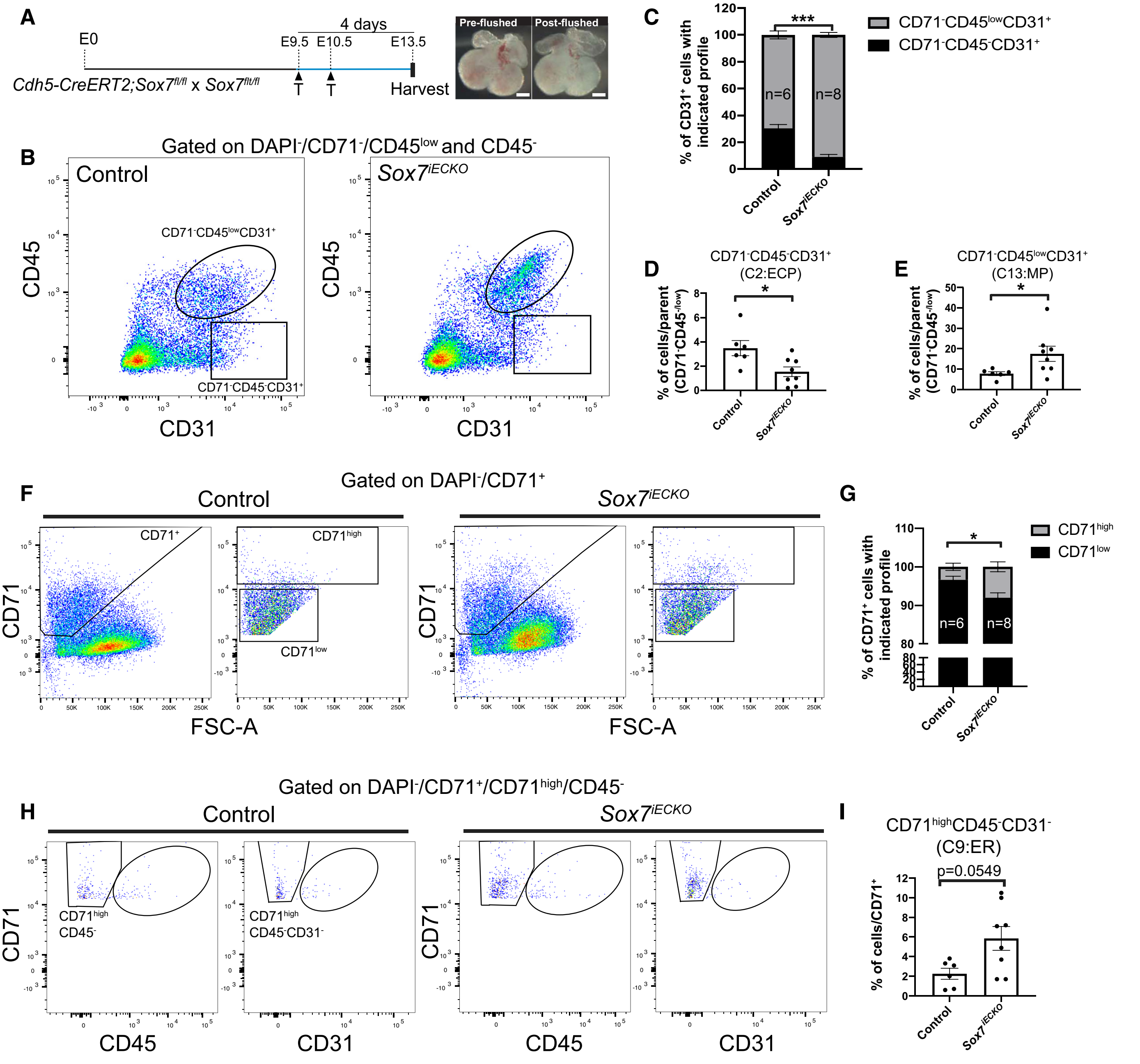

Figure 6. FACs analysis confirms a higher proportion of hematopoietic lineage cells in *Sox7*^{iECKO} hearts.

A Schematic representation of the tamoxifen injection course, where the pregnant dams were injected with tamoxifen at E9.5 and E10.5 and hearts were harvested at E13.5 (left). Hearts were flushed with saline buffer to remove circulating blood cells before dissociation into single cells for FACs' analysis (right).

B Flow analysis of hematopoietic and endothelial cells in control and *Sox7*^{iECKO} hearts. Results displayed a dramatic increase in the number of events associated with CD71^{-}CD45^{low}CD31^{+} hematopoietic cells (circled) but fewer CD71^{-}CD45^{-}CD31^{+} endothelial cells (rectangular box) in *Sox7*^{iECKO} hearts relative to the controls.

C Graphs showing the proportion of CD31^{+} cells that express CD45^{low} or CD45^{-} in E13.5 control and *Sox7*^{iECKO} hearts.

D, E Graphs showing the proportion of CD71^{-}CD45^{-}CD31^{+} endothelial cells (D) and CD71^{-}CD45^{low}CD31^{+} hematopoietic cells (E), both normalized to the CD71^{-}CD45$^{-/low}$ parent events.

F–I Flow analysis of early erythroblast (CD71^{high}, F; CD71^{high}CD45^{-}CD31^{-}, H) population, showing an increase in such events in the E13.5 control and *Sox7*^{iECKO} hearts. (G) Graphs showing the proportion of CD71^{high} cells in CD71-positive population of the E13.5 control and *Sox7*^{iECKO} hearts. (I) Graphs showing the proportion of CD71^{high}CD45^{-}CD31^{-} cells in CD71-positive population of the E13.5 control and *Sox7*^{iECKO} hearts.

Data information: (C–E, G, I) Biological replicates, n = 6 control hearts; n = 8 *Sox7*^{iECKO} hearts. Mean ± SEM; t-test. $P < 0.05$ (*); $P < 0.0005$ (***). Scale bars = 500 μm.

 © 2023 The Authors

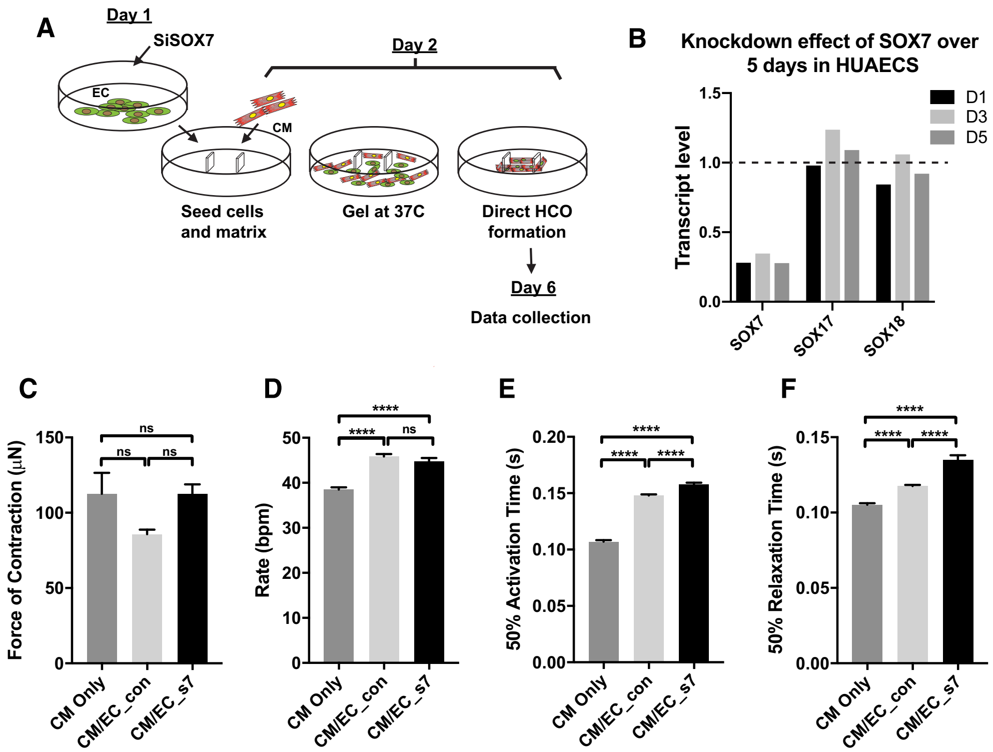

Figure 7. Cardiomyocyte function is affected by *Sox7* depletion in endothelial cells.

A Schematic showing the formation of endo/cardio-human cardiac organoids (hCO). *SOX7* was knocked down in human arterial endothelial cells (HUAECS) by siRNA transfection, then seeded with hiPSC-CMs and fibroblasts within extracellular matrix (ECM), and allowed to gel for 30 min at 37°C. Cells condense around the two elastomeric poles. Deflection of these poles can be used to approximate the contractile properties of each hCO.

B Quantitative PCR on HUAECs showing the degree of knockdown of SOX7 across 5 days following siRNA transfection. The dashed line refers to the expression level of gene expression in control samples and is arbitrarily set to 1.

C–F Contractile properties of endo/cardio-human cardiac organoid (hCO) showing the force of cardiomyocyte contraction (C), rate (D), 50% activation time (E), and 50% relaxation time (F). Overall contraction parameters ± SD from $n = 31$ endo/cardio-hCO cultured for 5 days in CTRL medium (10–11 hCO per condition). ****$P < 0.0001$ using one-way ANOVA with Tukey's post-test.

cardiomyocytes resulting from depleting *Sox7* in endothelial cells, by taking advantage of a human cardiac organoid (hCO) system (Mills *et al*, 2017). Briefly, *SOX7* was knocked down in HUAECs using siRNA-mediated gene silencing (Fig 7A). *SOX7* levels in HUAECs were consistently depleted to 60–70% of control levels upon siRNA treatment, with these levels maintained across 5 days of cell culture (Fig 7B). Knockdown was performed using a transient transfection at D0 with a single *SOX7* siRNA. These *SOX7*-depleted endothelial cells together with their respective controls were next seeded with human pluripotent stem cell-derived cardiomyocytes at Day 2 after transfection. The endothelial cell (EC)/human cardiac organoid (HCO) co-cultures were allowed to form for another 4 days before batch analysis of cardiac contractile parameters was performed for each condition (Fig 7A).

Interestingly, the co-culture of control-treated endothelial cells and cardiomyocytes significantly increased the contraction rate as well as the activation and relaxation time of cardiomyocytes (Fig 7C–F), supporting the idea that endothelial cells enhance the overall physiological performance of cardiomyocytes. In the presence of endothelial cells with siRNA-depleted *SOX7* levels, cardiomyocyte contractility showed a significant increase in both activation time and relaxation time when compared to control endothelial/cardiomyocyte co-culture conditions. This supra-physiological increase in relaxation time is comparable to effects caused by known pro-arrhythmic drugs such as propranolol or E4031 (Hoang *et al*, 2018). The dramatic increase in relaxation and activation time seen in *SOX7*-depleted conditions may relate to alterations in the extra cellular matrix composition and/or stiffness, or to altered levels of angiocrine signaling from endothelial cells. Our data suggest that *in vitro* depletion of *SOX7* function in endothelial cells is sufficient to influence the contractile properties of cardiomyocytes co-existing in culture.

Overall, several lines of experimental evidence support the idea that modulating endothelial cell identity by interfering with *Sox7* function has a dual outcome: (i) It leads to a lack of maintenance in endocardial cell identity which in turn disrupts the fine balance in the distribution of cardiac cell types, and (ii) these perturbations of cell identity promote morphological and functional cardiac defects caused by the alteration of heterotypic cell interaction between cardiac tissues.

Discussion

In this study, we have identified *Sox7* as a key regulator of cardiac endothelial cell identity and showed that SOX7-dependent cross-talk

between endothelial cells and cardiomyocytes controls the process of cardiac trabeculation and chamber myocardium compaction. At the cellular level, we showed that endothelial-specific loss of *Sox7* perturbs the finely tuned balance of different cardiac cell types during heart embryogenesis. Loss of endothelial *Sox7* promotes the reduction of an endothelial progenitor population and this results in the dysregulation of cardiomyocyte populations and erythro-myeloid lineages. We demonstrated that the dysregulation of *Sox7* in the endocardium promotes a hyper-trabeculation phenotype from E12.5–E13.5 that is not associated with increased proliferation of cells in the trabecular myocardium, but due to an increase in the allocation of cardiomyocytes to the trabecular layer from the compact myocardium.

Our results identified a reduction in the number of endocardial cells at the trabecular base, suggesting that the endocardial population depleted in the $Sox7^{iECKO}$ mutant embryos may derive from this location, and therefore, be involved in the control of cellular process occurring in the trabecular myocardium base. Cells of the trabecular myocardium are in an advanced differentiation state compared to the compact myocardium. Growth of the trabecular myocardium occurs primarily from the trabecular base, with oriented cell division allocating compact layer cardiomyocytes into the trabecular layer, where they differentiate into trabecular cardiomyocytes (de Boer *et al*, 2012; Li *et al*, 2016).

Our results suggest that endocardial *Sox7* may control the process by which cardiomyocytes are allocated to and differentiate in the trabecular layer. The increased undifferentiated cardiomyocyte population identified in the $Sox7^{iECKO}$ mutant samples in snRNA-Seq experiments may therefore represent a population of cardiomyocytes that have entered the trabecular layer but failed to totally differentiate into trabecular myocardium due to the lack of inductive signals from the missing basal endocardial population controlling the process. Therefore, this study positions *Sox7* as the key endothelial regulator of chamber myocardial morphogenesis and shows that signaling downstream of *Sox7* controls myocardial cell lineage determination and cellular behavior during heart development.

Our data further show *Sox7* is essential for coronary artery specification, and suggest that this is likely due to direct transcriptional regulation of Notch signaling and Connexin molecules. However, unlike the phenotype caused by the chromatin remodeler *Ino80* loss of function, the hyper-trabeculation and non-compaction defects in the $Sox7^{iECKO}$ mutant hearts are not related to coronary angiogenesis impairment. Instead, the coronary molecular and morphological phenotype identified in $Sox7^{iECKO}$ mutants suggest that the established molecular relationship among SOXF, Notch, and Connexin signaling controls the process of arterialization and hierarchization of coronary vessels during the late stages of heart formation.

The role of *sox7* in arterial specification has been reported in zebrafish vascular endothelium (Hermkens *et al*, 2015). Studies in mice confirmed its role and showed that *Sox7* controls arterial development through transcriptional regulation of the Notch ligand, *Dll4*, and its receptor, *Notch1* (Sacilotto *et al*, 2013; Chiang *et al*, 2017). In addition to its known role in the vascular endothelium, here we show that *Sox7* is also required for coronary artery formation. Our data also confirm *Dll4* as a downstream transcriptional target of SOX7. We also report additional direct target genes, including *Cx37* and *Cx40*, which encode gap junction proteins that play a key role in cell–cell communication.

Both *Cx37* and *Cx40* are expressed in the blood vascular endothelium, including in arterial endothelial cells and smooth muscle (Simon & McWhorter, 2002; Haefliger *et al*, 2004; Buschmann *et al*, 2010). *Cx37* has been shown to enable arterial specification through cell cycle arrest in a mouse retinal model (Fang *et al*, 2017). *Cx40* has been linked to flow-driven arteriogenesis and collateral arterial network development (Buschmann *et al*, 2010). Loss of *Cx40* was further shown to potentiate the appearance of arteriovenous shunts in *Alk1*-haploinsuffecent mice (Gkatzis *et al*, 2016), implicating *Cx40* as an important component in the maintenance of arterial cell identity. Recently, through a single-cell transcriptomic analysis during heart development, Su *et al* (2018) identified *Cx40* as a marker of arterial progenitor cells in the sinus venosus during coronary artery formation. This study showed that expression of *Cx40* is a critical intermediary step for arterial specification. In line with and expanding on these findings, our data suggest that *Sox7* modulates the emergence of pre-arterial cells via the direct regulation of a gene regulatory network that involves *Cx40*, *Cx37*, and Notch effectors (Sacilotto *et al*, 2013; Chiang *et al*, 2017).

Similar to the phenotype observed in the present study, failure to form the coronary arteries was reported in a mouse model of endothelial-specific loss of *Sox17* function (González-Hernández *et al*, 2020). Although the downstream molecular cascade regulated by SOX17 to instruct coronary artery formation remains to be elucidated, our study establishes that both *Sox7* and *Sox17* functions are indispensable for this process. While it is possible that SOX17 might regulate overlapping SOX7 target genes, such as those involved in Notch signaling (Corada *et al*, 2013), the lack of compensation by either molecule when the other is inactivated suggests they play non-redundant roles in artery formation. This is reinforced by our finding that SOX17 protein expression levels (Fig EV2B) remained unaffected in the absence of *Sox7*. Hence, the loss of coronary arteries due to altered *Sox7* function is not mediated through perturbed *Sox17* activity. Although both *Sox7* and *Sox17* are involved in coronary artery development, only *Sox7* is expressed in endocardium by E11.5 (Fig 1A and B; González-Hernández *et al*, 2020), suggesting that *Sox7* and *Sox17* have distinct molecular roles in the cardiac endothelium, especially at later developmental stages. It still remains to be determined if depleting a *Sox7*-positive endocardial cell pool directly contributes to the lack of coronary artery progenitors, or whether there is a failure of arterial differentiation independent from the endocardial contribution.

A role for SOXF transcription factors during endocardium differentiation has been reported for both SOX17 and SOX7 (Doyle *et al*, 2019; Saba *et al*, 2019; Hong *et al*, 2021). While *Sox17* is thought to regulate cardiomyocyte maturation in a non-cell autonomous manner, *Sox7* was proposed to directly promote the endothelial lineage cell fate at the expense of the cardiac lineage through positive regulation of Wnt and BMP signaling in embryoid bodies (Doyle *et al*, 2019). Echoing this *in vitro* finding, we report that deletion of *Sox7* in cells committed to an endothelial lineage causes an increase in a subpopulation of cardiomyocytes. Nonetheless, lineage-tracing analysis using the *Cdh5-CreERT2:*$Sox7^{fl/fl}$*;mT/mG* mouse did not reveal any GFP-positive cardiomyocytes, suggesting that the expansion of the cardiomyocyte population observed in $Sox7^{iECKO}$ mutants is not due to transdifferentiation from a committed endothelial lineage into the cardiomyocyte lineage (Appendix Fig S7). However, we cannot discount the possibility that SOX7 plays a critical role at an early time point prior to fate

 © 2023 The Authors

specification when progenitor cells retain the bipotentiality to give rise to either endothelial or cardiac lineages. Our data are consistent with the possibility that the increase in cardiomyocyte number in the absence of *Sox7* is due to non-cell-autonomous effects from the cardiac endothelium to the myocardium, akin to what has been described in mice that are deficient for *Sox17*, *Ino80*, and Notch mutants, *Fkbp1a* and *Jarid2* (Mysliwiec *et al*, 2011; Chen *et al*, 2013; Saba *et al*, 2019; Rhee *et al*, 2021).

In addition to a failure to specify coronary arteries, our work shows that loss of *Sox7* function depletes a subpopulation of endothelial cells in the developing heart. The enriched expression of endocardial marker *Nrp3* and the absence of coronary markers *Fabp4* and *Apelin* suggest that this population is mainly endocardial in nature. This observation coincides with an increase in the overall proportion of hematopoietic cells, specifically the $CD45^{low}CD31^{+}$ hematopoietic cells and $CD71^{high}CD45^{-}CD31^{-}$ erythroblasts (Fig 6), and a subset of cardiomyocytes. Cells expressing both CD45 and CD31 have been previously associated with the hematopoietic stem cell niche (Shaw *et al*, 2004). Interestingly, *Sox7* has previously been shown to play a critical role in directing endothelial specification and lineage maintenance in the hemogenic endothelium, with its downregulation required for the subsequent emergence of hematopoietic progenitors (Gandillet *et al*, 2009; Costa *et al*, 2012; Lim *et al*, 2016). Akin to both the dorsal aorta and the yolk sac vasculature, the heart endocardium has been shown to have the capacity to undergo endothelial-to-hematopoietic (EHT) transition from E9.5 in the mouse (Nakano *et al*, 2013). This transition is observed in a specific group of cells integrated into the outflow cushion and atria called the hemogenic endocardial cells. Later in development between E11 and E14, aggregates of endothelial and hematopoietic cells called the blood islands are formed, emerging from the endocardium (Red-Horse *et al*, 2010; Jankowska-Steifer *et al*, 2015). Consistent with the role of *Sox7* in the hemogenic niche in other organs, analysis of E13.5 embryonic hearts from a *Cdh5-CreERT2:Sox7*$^{fl/fl}$*; mT/mG* reporter line shows that GFP-positive endothelial cells (e.g., Sox7 null) harbor the expression of hematopoietic stem cell markers CD34 and pan-hematopoietic marker CD45 (Fig EV5). These results suggest an important role for SOX7 in the maintenance of the endocardial identity by preventing its transdifferentiation toward a hematopoietic fate.

In summary, we have identified *Sox7* as a key regulator of cardiac endothelium identity. The present study further supports the pivotal requirement for a specified, differentiated endothelium to instruct heart morphogenesis (Mysliwiec *et al*, 2011; Chen *et al*, 2013; Qu *et al*, 2019; Sandireddy *et al*, 2019; Rhee *et al*, 2021). The finely tuned regulation of the number and state of cardiac endothelial cells by *Sox7* and its downstream effectors is intricately linked to the distribution of other cardiac cell types, which in turn determines tissue architecture, assembly, and function during cardiac development. This finding sheds light on a potential novel etiological component of non-compaction cardiomyopathies.

Materials and Methods

Transgenic mice and fish

Mice used in this study were *Sox7:tm1* (Chiang *et al*, 2017), *Cdh5-CreERT2* (Wang *et al*, 2010), *Sox7*$^{fl/fl}$ (Lilly *et al*, 2017), *Sox7-V5* (Chiang *et al*, 2023), *Sox7*$^{\Delta ex2/\Delta ex2}$ (Wat *et al*, 2012), and *mT/mG* (Muzumdar *et al*, 2007). *Sox7* endothelial-specific conditional knockout (C57BL/6) was a cross between *Cdh5-CreERT2* and *Sox7*$^{fl/fl}$. To delete *Sox7* in endothelial cells, two consecutive intraperitoneal injections of tamoxifen (T5648, Sigma Aldrich), at 2 mg per pulse, were administered to dams at E9.5 and E10.5, and embryos were collected at the indicated time points. To detect the primary gene targets of SOX7, pregnant dams were pulsed with tamoxifen at E9.5, and embryos were harvested for FACs sorting 24 h after Cre induction. Cx40-21:GFP was generated as described in Chiang *et al* (2017) by cloning a 566 bp PCR fragment from human genomic DNA into the zebrafish enhancer detector (ZED) vector (Bessa *et al*, 2009). All animal work was approved by the relevant ethics committees of the University of Queensland and University of Sydney ethics ID: 084/19 Experimental Analysis of Lymphangiogenesis in Development and Regeneration.

Immunofluorescence staining and tissue sectioning

Mouse embryos were fixed in 4% paraformaldehyde for either 1 h or overnight at 4°C, depending on the developmental stage, and then washed three times in PBS. Tissue embedding and sectioning were performed according to standard protocols. Immunofluorescence staining of mouse embryos was performed as described in Chiang *et al* (2023). Immunofluorescence of tissue sections for the characterization is shown in Figs 2 and EV1, and Appendix Fig S1, and was performed as described in Del Monte-Nieto *et al* (2018).

Antibodies used were as follows: anti-EMCN (1:300, sc-53941, Santa Cruz), anti-SOX7 (1:300, AF2766, R&D System), anti-SOX17 (1:300, AF1924, R&D System), anti-ERG (1:300, AB92513, Abcam), anti-phospho-histone H3 (1:500; H9908, Sigma Aldrich), anti-V5 (1:200, AB3792, Merck), anti-TPM (1:500; T9283, Sigma), anti-VEGFR2 (1:200, AF644, R&D System), anti-CX40 (1:300, CX-40A, Alpha Bio-diagnostic), anti-SMA (1:200, 14395-1-AP, Proteintech), anti-CD31 (1:200, DIA-310, Dianova), anti-MF20-488 (1:500, 53-6503-80, Thermo Fisher Scientific), anti-CD34 (1:300, rat 13-0341-81 ebioscience), anti-CD45 (1:300, rat 53082 BD Pharmingen) and anti-Ki67 (1:200, ab15580, Abcam). Nuclear counter-stainings were performed using DAPI (1:1,000, D9542, Sigma Aldrich) or Hoechst 33342 (1:1,000, B2261, Sigma Aldrich).

Secondary antibodies were as follows: donkey anti-rat IgG Alexa 488 (A21208), goat anti-mouse IgG Alexa 594 (A11005), donkey anti-rabbit IgG Alexa 647 (A31573), donkey anti-goat IgG Alexa 647 (A21447), and donkey anti-rabbit Alexa 594 (A21207). Secondary antibodies were sourced from Invitrogen and used at 1:300 unless specified otherwise. For morphological characterizations, CD31 was amplified using anti-rat-HRP (1:100, A18745, Thermo Fisher Scientific) followed by tyramide-Cy5 amplification (NEL745001KT; Perkin Elmer); Ki67 was amplified using anti-rabbit-Cy5 (1:100, A31572, Thermo Fisher Scientific).

Cell isolation for snRNA-Seq

Cdh5-CreERT2, *Sox7*$^{fl/fl}$ males were crossed with *Sox7*$^{fl/fl}$ females, who were dosed with tamoxifen at E9.5 and E10.5. Hearts were harvested from E12.5 embryos, and immediately snap frozen in liquid nitrogen before storage at −80°C. Subsequently, four hearts from similar genotypes were pooled, and single nuclei extraction

© 2023 The Authors

and isolation were performed as described, with modifications (Sim *et al*, 2021). Briefly, isolation buffer was 0.3 M sucrose, 10 mM Tris–HCl, 5 mM magnesium acetate, 5 mM $CaCl_2$, 2 mM EDTA, 0.5 mM EGTA, and 1 mM DTT. Pooled hearts were suspended in isolation buffer and homogenized with a 15 ml tissue grinder (357544, Edwards Wheaton). Samples were then filtered through a 40 μM cell strainer, washed, and finally, resuspended in PBS saline buffer containing Hoechst stain (1:1,000, Invitrogen). Nuclei were then sorted using a BD Influx™ Cell Sorter with a 70 μM nozzle, before loading onto the 10× chromium single-cell chip (v3, 10× Genomics).

Imaging and data analysis

Images were captured with a Zeiss LSM710 META BIG, Zeiss LSM 710 FCS or Leica TCS SP8 HyD confocal microscopes with the 10×, 20× or 40× oil objectives. Images were analyzed using the Bitplane IMARIS suite and Image J. Imaging was performed in the Australian Cancer Research Foundation (ACRF)'s Dynamic Imaging Facility at the Institute for Molecular Bioscience (University of Queensland) and the Sydney Cytometry facilities at Centenary Institute (University of Sydney). All graphs and statistical tests were performed with Graphpad Prism 9 and illustrated with Adobe Illustrator CS6. Morphological, cell count, and proliferation experiments were imaged using a Leica SP8 inverted microscope at Monash Microimaging platform.

Image quantification and data analysis

Image analysis to perform tissue area, quantification of cell number, and actively proliferating cells was performed in Fiji. Image channels were isolated and overlaid with a mask for area quantification and nuclear classification performed via intensity thresholding. We generated endocardial, myocardial, and epicardial masks. Myocardial masks were further segmented into trabecular and compact myocardial masks by manual selection of the compact layer boundary following endocardial touchdowns as described in Del Monte-Nieto *et al* (2018). The luminal boundary of the trabecular myocardium and endocardium was found by iteratively expanding this compact layer selection in a pixel-wise manner until it encompassed the entirety of the tissue; the distance at which all tissue was enclosed was labeled as the luminal apical boundary, with the basal/apical boundary defined as halfway between the compact myocardium and the apex of the trabeculae. This allowed further sub-segmentation of the endocardial and trabecular myocardial masks into basal and apical layers. Nuclear segmentation was performed with the StarDist plugin in Fiji (Schmidt *et al*, 2018). Individualized nuclei were subsequently classified using the previously generated tissue masks. This identified nuclei from cells forming the compact myocardium, basal and apical trabecular myocardium, epicardium, and basal and apical endocardium. The average intensity of Ki67 staining within each nucleus was then measured, and nuclei were further classified as either $Ki67^{+}$ or $Ki67^{-}$ based on a calibrated threshold; positive nuclei were labeled red and negative blue. The data collected were scaled to the characteristic length of the specific tissue area analyzed and normalized to the control value. Once scaled and normalized, data were plotted using GraphPad Prism. Data were analyzed for normal distribution using Shapiro–Wilk test. Once confirmed, statistical analysis was performed using parametric Student's *t*-test with significance achieved when *P*-value was < 0.05. Data were represented in the graph as mean ± SEM. For a summary of the quantification process, see Appendix Fig S9.

Cloning

To generate the CX40-21 construct, a 566 bp fragment of the human CX40 region (GRCh37/hg19_dna range: Chr1: 147,266,531–147,267,096) was amplified from human umbilical venous endothelial cell (HUVEC) (CC-2519A) genomic DNA and subcloned into the linearized zebrafish enhancer detector (ZED) vector, with the following primers (attB1 and attB2 cloning sites are underlined):

hCX40-21_F: <u>GGGGACAAGTTTGTACAAAAAAGCAGGCT</u>GAGCCTAGGCACAGGGAAT
hCX40-21_R: <u>GGGGACCACTTTGTACAAGAAAGCTGGGTA</u>CACCAGATTGGTATTGGGTTTCT

Construct was generated using Gateway Cloning Technology (Thermo Fisher Scientific).

Single-cell dissociation and fluorescent-activated cell sorting (FACS)

To validate the $Sox7^{iECKO}$ E10.5 RNA-Seq, staged matched (31–33 ss) sibling controls and $Sox7^{iECKO}$ embryos were harvested from pregnant dams treated as specified with tamoxifen at E9.5. Whole embryos were subjected to single-cell isolation using 0.071 mg/ml liberase in PBS buffer. $CD31^{+}$ endothelial cells were isolated using Alexa 488–conjugated rat anti-CD31 (1:800, 563607, BD Pharmigen), and live/dead cells were detected by 7AAD (1:1,000, Invitrogen). Cell sorting was performed on a BD FACSAria Cell Sorter.

To validate the number of hematopoietic and cardiac endothelial cells observed in snRNA-Seq (Figs 5 and EV3), control and $Sox7^{iECKO}$ mutants hearts at E12.5 were dissected in ice-cold PBS, and circulating blood cells were flushed using a microinjection needle filled with saline PBS. Hearts were dissociated in 0.071 mg/ml liberase in PBS buffer, supplemented with 0.25 mg/ml collagenase II, 0.25 mg/ml collagenase IV, 1 mg/ml deoxyribonuclease I, 0.9 mM $CaCl_2$, and 10 mM Hepes, followed by incubation at 37°C for 5 min and pipetting with P200 pipette tips every 2 min to assist tissue dissociation. Tissue digestion was stopped by addition of 2 mM $CaCl_2$ and 10% fetal calf serum. Digestion buffer was next removed before passing the dissociated samples through a 70 μm cell strainer, washed once then resuspended in Fc Block (1:200, BD Bioscience) followed by 10 min blocking on ice. Dissociated cells were stained with appropriate antibodies for 0.5 h on ice. Antibodies used were Alexa 488-conjugated rat anti-CD31 (1:200, 563607, BD Pharmigen), Brilliant Violet 510-conjugated rat anti-CD71 (1:200, R17217, BioLegend), and PE/Cyanine 7-conjugated rat anti-CD45 (1:400, 30-F11, BioLegend). Live/dead cells were detected by DAPI (1:1,000, D9542, Sigma Aldrich). For compensation, UltraComp eBeads (Invitrogen) were used as per manufacturer's instruction. Samples were washed twice, and then two hearts from similar genotypes were pooled (following genotyping by PCR). Pooled samples were finally resuspended in 200 μl 1% FCS/PBS/2 mM EDTA solution containing DAPI. Cell sorting was performed on a BD FACSAria™ Cell Sorter with a 100 μM nozzle. FACs sorting was performed in Sydney Cytometry Facilities at Centenary Institute (University of Sydney).

© 2023 The Authors

Bioinformatic analysis of single-nucleus RNA-Seq

Sequence reads were aligned with STAR Solo (v2.7.3) (preprint: Kaminow *et al*, 2021) to the mouse genome (mm10) using the gene model provided with 10× Genomics Cell Ranger 1.2.0 to generate UMI counts for 33,939 genes. The parameters used for STAR Solo alignment were as follows:

--soloAdapterMismatchesNmax 1 --soloUMIlen 12 --genomeSA sparseD 3 --soloUMIfiltering MultiGeneUMI --soloCBmatchWLtype 1MM_multi_pseudocounts --soloCBwhitelist 3 M-february-2018.txt --soloBarcodeReadLength 28

Single-cell gene counts were analyzed using the R package Seurat (v3) (Stuart *et al*, 2019). The control or *Sox7*^{iECKO} snRNA-Seq datasets were independently normalized and identified variable features prior to defining anchor features (function FindIntegrationAnchors with nfeatures = 2,000). Both datasets were then integrated before being scaled and principal component analysis (functions ScaleData, RunPCA). Twenty principal components were used for graph-based clustering at a resolution of 0.3 (functions FindNeighbours and FindClusters) and UMAP and *t*-SNE dimensionality reduction were then computed (functions RunTSNE, RunUMAP). Cell clusters were classified by identifying enriched gene sets (function FindMarkers).

Monocle3 (Trapnell *et al*, 2014) package (v3.0.0) in R (version 4.1.1) was used to analyze pseudo-time of development on integrated data in Appendix Fig S6. For the primary analysis, all cell clusters from either control or *Sox7*^{iECKO} mutant hearts were subsetted into separate Seurat objects and then analyzed with Monocle3 using the default parameters. For C2 negative analysis, the subsetted control or *Sox7*^{iECKO} Seurat objects were then further subsetted to remove all but five C2-defined cells before being subjected to Monocle3 analysis. When displaying Monocle3 data, we replaced the Monocle3-defined clusters with our predefined cluster identifications.

RNA-Seq and analysis

For RNA-Seq on FACS-sorted mouse endothelial cells at E10.5, triplicate samples were processed for whole transcriptome sequencing using the Smart-Seq 2 method as described in Picelli *et al* (2014). Samples were sequenced using the Illumina™ HiSeq 2500 platform. Reads were then mapped to GRCm38/mm10 mouse reference genome using STAR aligner (Dobin *et al*, 2013). Only unique aligned reads were considered. Next, transcripts were assigned to mouse genes using htseq_count in the HTSeq python package (Anders *et al*, 2015) and differential expression between sibling control and *Sox7*^{iECKO} mutants was calculated using DEseq2 (Love *et al*, 2014) and EdgeR (Robinson *et al*, 2010). Only genes with adjusted *P*-value < 0.05 in both DEseq2 and EdgeR were considered significant.

Heatmaps for the RNA-Seq results in Appendix Fig S4A and C were generated using pheatmap R package (Kolde *et al*, 2012), while the bubble chart depicting gene ontological terms in Appendix Fig S4B was plotted with ggplot2 R package (Wickham, 2009).

RNA amplification, cDNA synthesis, and real-time PCR

cDNA was synthesized using High-Capacity cDNA Reverse Transcription Kit (Life technologies) from 1,000 ng purified RNA. qRT–PCR was performed and analyzed as described in Chiang *et al* (2017). For FACs-sorted mouse endothelial cells, RNA was extracted and amplified, and cDNA was synthesized as described in Chiang *et al* (2017) and Picelli *et al* (2014). To quantify the transcript level of target genes, the following primers were used:

Mouse

mSox7_F: 5′-GCGGAGCTCAGCAAGATG-3′
mSox7_R: 5′-GGGTCTCTTCTGGGACAGTG-3′
mDll4_F: 5′-AGGTGCCACTTCGGTTACAC-3′
mDll4_R: 5′-GGGAGAGCAAATGGCTGATA-3′
mPecam1_F: 5′-CGGTGTTCAGCGAGATCC-3′
mPecam1_R: 5′-ACTCGACAGGATGGAAATCAC-3′
mCdh5_F: 5′-GTTCAAGTTTGCCCTGAAGAA-3′
mCdh5_R: 5′-GTGATGTTGGCGGTGTTGT-3′
mCx40_F: 5′-ACTGTTATGTTTCGAGGCCCA-3′
mCx40_R: 5′-CCCAGGTGGTAGAGTTCAGC-3′
mCx37_F: 5′-GGCAAGCAGGCGAGAGA-3′
mCx37_R: 5′-TGCGGAAGATGAAGAGCACC-3′
mVwf_F: 5′-TGGTCCTGAAGCACACATACC-3′
mVwf_R: 5′-GACGGGGTCTTCCTCCAC-3′
mCol3a1_F: 5′-TGACTGTCCCACGTAAGCAC-3′
mCol3a1_R: 5′-GAGGGCCATAGCTGAACTGA-3′
mPdlim3_F: 5′-TTGACAGGGCAGAAACTCGC-3′
mPdlim3_R: 5′-CGCGGTACCAATGGGTTTGA-3′
mTnni1_F: 5′-CACGAGGACTAAACTAGGCACT-3′
mTnni1_R: 5′-AGCATGAGTTTACGGGAGGC-3′
mCdkn1c_F: 5′-CAGCCAGCCTTCGACCAT-3′
mCdkn1c_R: 5′-ACTGGGAAGGTATCGCTGGA-3′
mLyve1_F: 5′-GGTGTCCTGATTTGGAATGC-3′
mLyve1_R: 5′-AGGAGTTAACCCAGGTGTCG-3′

Human

hSOX7_F: 5′-AGCTGTCGGATGGACAATCG-3′
hSOX7_R: 5′-CCACGACTTTCCCAGCATCT-3′
hSOX17_F: 5′-AAGGGCGAGTCCCGTATCC-3′
hSOX17_R: 5′-CACGACTTGCCCAGCATCTTG-3′
hSOX18_F: 5′-CAAGATGCTGGGCAAAGCGTG-3′
hSOX18_R: 5′-GCGGGGGCGCTAATCC-3′
hVEGFR3_F: 5′-ATAGACAAGAAAGCGGCTTCA-3′
hVEGFR3_R: 5′-CCTCCCTTGGGAGTCAGG-3′
hVEGFR2_F: 5′-GAACATTTGGGAAATCTCTTGC-3′
hVEGFR2_R: 5′-CGGAAGAACAATGTAGTCTTTGC-3′
hCX40_F: 5′-TCGTTATGGCCAGAAGCCTG-3′
hCX40_R: 5′-TGGCCTTACTAAGACGTCGC-3′
hCX37_F: 5′-GAATGTGGCTTTGCCTGAGC-3′
hCX37_R: 5′-TGAGGTTGGGGTAAGTTGGC-3′
hVWF_F: 5′-CACAGGGGGACCAAAGAGTC-3′
hVWF_R: 5′-ACAAAGCCATAGGCCTCACC-3′
hDLL4_F: 5′-CCATGCAAGAATGGGGCAAC-3′
hDLL4_R: 5′-GCCATCCTCCTGGTCCTTAC-3′
hERG_F: 5′-TCGATGGGAAGGAACTGTGC-3′
hERG_R: 5′-GGAAGAGGAGTCTCTCTGAGGT-3′
hGAPDH_F: 5′-CCCCGGTTTCTATAAATTGAGC-3′
hGAPDH_R: 5′-CACCTTCCCCATGGTGTCT-3′
hHPRT_F: 5′-AATGACCAGTCAACAGGGGACA-3′
hHPRT_R: 5′-TACTGCCTGACCAAGGAAAGCA-3′

© 2023 The Authors

Cell culture and siRNA-mediated silencing experiment

For gene expression study in Fig 4B, cell culture and gene silencing experiment was conducted in human umbilical arterial endothelial cells (HUAECs) (C-12202), using Smartpool: ON-TARGETplus Human *SOX7* (83595) siRNA 50 nM (containing a mixture of four siRNA mixes against *SOX7*) (Dharmacon) and scrambled siRNA (Dharmacon) (Chiang *et al*, 2023). Cells were harvested 48 h post-transfection for RNA extraction with the Direct-Zol RNA kit (Zymo Research).

For co-culturing between *SOX7* knockdown endothelial cells with human pluripotent stem-cell-derived cardiac organoid, gene silencing was conducted as described above, except that cells were harvested at day 3 and day 5 post-gene silencing to assess the transcript levels of SOXF transcription factors.

Heart dyno assay and hCO fabrication

Cardiac cells were differentiated, and hCO and Heart-Dyno culture inserts were fabricated as described in Mills *et al* (2017).

All cell lines used in this study have tested negative for mycoplasma contamination.

In situ hybridization assay

Whole-mount and section *in situ* hybridization was performed as described in Fowles *et al* (2003) and Metzis *et al* (2013). *Cx37* (clone ID: 4971608) probe was from Dharmacon. *In situ* hybridization on tissue sections was performed as described in Kanzler *et al* (1998). *Cx40* probe was generated by Dr. Gonzalo del Monte-Nieto while working at Prof. Richard Harvey Laboratory. Please contact Dr. Gonzalo del Monte-Nieto for more details.

HCR and immunofluorescence.

Slides were taken from −80°C and incubated for 20 min in the oven at 65°C. Dewaxing was done by placing the slides in xylene for 3 × 10 min, dehydrating them in ethanol series (100% 2 × 10 min, 90% 2 × 5 min, 70% 5 min, 50% 5 min, and 30% 5 min), and finally, washing them in 1 × PBS for 2 × 5 min. Slides were incubated in 200 µl of probe hybridization buffer and prehybridized in the humidified chamber at 37°C. 2.4 pmol of probe solution was added using the following probes: *Gpc3* (NM_016697.3, Molecular Instrument), *Sox9* (NM_011448.4, Molecular Instrument), *Hopx* (NM_175606.3, Molecular Instrument), and *Nppa* (NM_008725.3, Molecular Instrument). After overnight incubation at 37°C, excess probes were removed by adding 100% probe wash buffer (75% PWB/25% SSCT, 50% PWB/50% SSCT, 25% PWB/75% SSCT, and 100% SSCT) for 15 min each. 200 µl of amplification buffer was added and sections were pre-amplified for 30 min at RT. 12 pmol of hairpin (h1 and h2) was prepared separately by heating at 95°C for 90 s and then cooling at RT for 30 min. 200 µl of hairpin solution was added on the samples and incubated o/n at RT. Excess hairpins were removed by washing with 5× SSCT for 40 min. Immunofluorescence protocol was followed from this point. Sections were incubated in primary antibody: endomucin (sc-65495, Santa Cruz Biotechnology) o/n and secondary antibody: goat anti-rat-A680 (A21096, Thermo Fisher Scientific) for 2 h at RT. The slides were washed with 0.3% Triton-X100 in PBS for 2 × 10 min after each step. Sections were then counterstained with SMA-FITC (F3777-2ML, Sigma-Aldrich) and Hoechst (B2261, Sigma-Aldrich).

Graphics

The synopsis graphics were created with BioRender.com

Data availability

Bulk RNA-seq and snRNA-seq datasets have been deposited to the GEO repository under unique identifiers: (i) snRNAseq and bulk RNAseq in conditional Sox7 knock-out: Gene Expression Omnibus GSE231636 (https://www.ncbi.nlm.nih.gov/geo/query/acc.cgi?acc=GSE231636). (ii) Bulk RNAseq in constitutive Sox7ko: Gene Espression Omnibus GSE236846 (https://www.ncbi.nlm.nih.gov/geo/query/acc.cgi?acc=GSE236846). Raw data images have been deposited to DataDryad repository. To review data dryad accession: (DOI): https://doi.org/10.5061/dryad.qv9s4mwm3. Access prior to public release: https://datadryad.org/stash/share/vQG6vb3l5sJZH3YJWGZQ9YIKQ8fGYDa7uF0XeAovl40.

Expanded View for this article is available online.

Acknowledgements
The authors would like to thank Dr Emma Gordon for the *mT/mG* mice. We warmly thank Dr Carol Wicking for her scientific comments and advice on editing the manuscript. This research was supported by National Health and Medical Research Council (NHMRC) grant and fellowship (APP2019904, APP116400, and APP1111169), NSW Health Cardio-Vascular Research Network and Australian Research Council (ARC) grant (DP200100250) to MF, Heart Foundation of Australia Future Leader Fellowship (102036) and ARC grants (DP190101475 and DP210102134) to GdM-N, and National Institutes of Health grant (HD098458) to DAS. Open access publishing facilitated by The University of Sydney, as part of the Wiley - The University of Sydney agreement via the Council of Australian University.

Author contributions
Ivy KN Chiang: Conceptualization; resources; data curation; formal analysis; supervision; validation; investigation; visualization; methodology; writing – original draft; project administration; writing – review and editing. **David Humphrey:** Data curation; formal analysis; supervision; investigation; visualization; methodology; project administration; writing – review and editing. **Richard J Mills:** Formal analysis; investigation; writing – review and editing. **Peter Kaltzis:** Data curation; formal analysis; validation; investigation; methodology. **Shikha Pachauri:** Formal analysis; investigation. **Matthew Graus:** Investigation. **Diptarka Saha:** Formal analysis; investigation; methodology. **Zhijian Wu:** Formal analysis; validation; investigation. **Paul Young:** Data curation; formal analysis; visualization; writing – review and editing. **Choon Boon Sim:** Formal analysis; investigation; methodology. **Tara Davidson:** Resources; formal analysis; investigation. **Andres Hernandez-Garcia:** Investigation; methodology. **Chad A Shaw:** Data curation. **Alexander Renwick:** Data curation.
Daryl A Scott: Data curation; formal analysis; funding acquisition; investigation; writing – review and editing. **Enzo R Porrello:** Data curation; formal analysis; writing – review and editing. **Emily S Wong:** Data curation; formal analysis; visualization; methodology; writing – review and editing.

© 2023 The Authors

James E Hudson: Formal analysis; methodology; writing – review and editing. **Kristy Red-Horse:** Formal analysis; investigation; writing – review and editing. **Gonzalo del Monte-Nieto:** Data curation; formal analysis; supervision; funding acquisition; validation; visualization; methodology; writing – original draft; project administration; writing – review and editing. **Mathias Francois:** Conceptualization; resources; data curation; formal analysis; supervision; funding acquisition; visualization; methodology; writing – original draft; project administration; writing – review and editing.

Disclosure and competing interests statement

The authors declare that they have no conflict of interest.

References

Afouda BA, Lynch AT, de Paiva AE, Hoppler S (2018) Genome-wide transcriptomics analysis identifies sox7 and sox18 as specifically regulated by gata4 in cardiomyogenesis. *Dev Biol* 434: 108–120

Anders S, Pyl PT, Huber W (2015) HTSeq—a Python framework to work with high-throughput sequencing data. *Bioinformatics* 31: 166–169

Benedito R, Trindade A, Hirashima M, Henrique D, da Costa L, Rossant J, Gill P, Duarte A (2008) Loss of Notch signalling induced by Dll4 causes arterial calibre reduction by increasing endothelial cell response to angiogenic stimuli. *BMC Dev Biol* 8: 1–15

Bessa J, Tena JJ, de la Calle-Mustienes E, Fernández-Miñán A, Naranjo S, Fernández A, Montoliu L, Akalin A, Lenhard B, Casares F *et al* (2009) Zebrafish enhancer detection (ZED) vector: a new tool to facilitate transgenesis and the functional analysis of cis-regulatory regions in zebrafish. *Dev Dyn* 238: 2409–2417

Buschmann I, Pries A, Styp-Rekowska B, Hillmeister P, Loufrani L, Henrion D, Shi Y, Duelsner A, Hoefer I, Gatzke N *et al* (2010) Pulsatile shear and Gja5 modulate arterial identity and remodeling events during flow-driven arteriogenesis. *Development* 137: 2187–2196

Buszczak M, Signer RA, Morrison SJ (2014) Cellular differences in protein synthesis regulate tissue homeostasis. *Cell* 159: 242–251

Chao R, Gong X, Wang L, Wang P, Wang Y (2015) CD71^{high} population represents primitive erythroblasts derived from mouse embryonic stem cells. *Stem Cell Res* 14: 30–38

Chen H, Zhang W, Sun X, Yoshimoto M, Chen Z, Zhu W, Liu J, Shen Y, Yong W, Li D *et al* (2013) Fkbp1a controls ventricular myocardium trabeculation and compaction by regulating endocardial Notch1 activity. *Development* 140: 1946–1957

Chiang IK-N, Fritzsche M, Pichol-Thievend C, Neal A, Holmes K, Lagendijk A, Overman J, Angelo D, Omini A, Hermkens D *et al* (2017) SoxF factors induce Notch1 expression via direct transcriptional regulation during early arterial development. *Development* 144: 2629–2639

Chiang IKN, Graus MS, Kirschnick N, Davidson T, Luu W, Harwood R, Jiang K, Li B, Wong YY, Moustaqil M *et al* (2023) The blood vasculature instructs lymphatic patterning in a SOX7-dependent manner. *EMBO J* 42: e109032

Colliva A, Braga L, Giacca M, Zacchigna S (2020) Endothelial cell–cardiomyocyte crosstalk in heart development and disease. *J Physiol* 598: 2923–2939

Corada M, Orsenigo F, Morini MF, Pitulescu ME, Bhat G, Nyqvist D, Breviario F, Conti V, Briot A, Iruela-Arispe ML *et al* (2013) Sox17 is indispensable for acquisition and maintenance of arterial identity. *Nat Commun* 4: 2609

Costa G, Mazan A, Gandillet A, Pearson S, Lacaud G, Kouskoff V (2012) SOX7 regulates the expression of VE-cadherin in the haemogenic endothelium at the onset of haematopoietic development. *Development* 139: 1587–1598

de Boer BA, van den Berg G, de Boer PA, Moorman AF, Ruijter JM (2012) Growth of the developing mouse heart: an interactive qualitative and quantitative 3D atlas. *Dev Biol* 368: 203–213

de Lange FJ, Moorman AFM, Anderson RH, Männer J, Soufan AT, de Gier-de Vries C, Schneider MD, Webb S, van den Hoff MJ, Christoffels VM (2004) Lineage and morphogenetic analysis of the cardiac valves. *Circ Res* 95: 645–654

Del Monte-Nieto G, Ramialison M, Adam AAS, Wu B, Aharonov A, D'Uva G, Bourke LM, Pitulescu ME, Chen H, de la Pompa JL *et al* (2018) Control of cardiac jelly dynamics by NOTCH1 and NRG1 defines the building plan for trabeculation. *Nature* 557: 439–445

Dobin A, Davis CA, Schlesinger F, Drenkow J, Zaleski C, Jha S, Batut P, Chaisson M, Gingeras TR (2013) STAR: ultrafast universal RNA-seq aligner. *Bioinformatics* 29: 15–21

Dong HY, Wilkes S, Yang H (2011) CD71 is selectively and ubiquitously expressed at high levels in erythroid precursors of all maturation stages: a comparative immunochemical study with glycophorin A and hemoglobin A. *Am J Surg Pathol* 35: 723–732

Doyle MJ, Magli A, Estharabadi N, Amundsen D, Mills LJ, Martin CM (2019) Sox7 regulates lineage decisions in cardiovascular progenitor cells. *Stem Cells Dev* 28: 1089–1103

Fang JS, Coon BG, Gillis N, Chen Z, Qiu J, Chittenden TW, Burt JM, Schwartz MA, Hirschi KK (2017) Shear-induced Notch-Cx37-p27 axis arrests endothelial cell cycle to enable arterial specification. *Nat Commun* 8: 2149

Ferdous A, Caprioli A, Iacovino M, Martin CM, Morris J, Richardson JA, Latif S, Hammer RE, Harvey RP, Olson EN *et al* (2009) Nkx2–5 transactivates the Ets-related protein 71 gene and specifies an endothelial/endocardial fate in the developing embryo. *Proc Natl Acad Sci USA* 106: 814–819

Fowles LF, Bennetts JS, Berkman JL, Williams E, Koopman P, Teasdale RD, Wicking C (2003) Genomic screen for genes involved in mammalian craniofacial development. *Genesis* 35: 73–87

François M, Caprini A, Hosking B, Orsenigo F, Wilhelm D, Browne C, Paavonen K, Karnezis T, Shayan R, Downes M *et al* (2008) Sox18 induces development of the lymphatic vasculature in mice. *Nature* 456: 643–647

Fuchs C, Gawlas S, Heher P, Nikouli S, Paar H, Ivankovic M, Schultheis M, Klammer J, Gottschamel T, Capetanaki Y *et al* (2016) Desmin enters the nucleus of cardiac stem cells and modulates Nkx2.5 expression by participating in transcription factor complexes that interact with the nkx2.5 gene. *Biol Open* 5: 140–153

Gandillet A, Serrano AG, Pearson S, Lie-A-Ling M, Lacaud G, Kouskoff V (2009) Sox7-sustained expression alters the balance between proliferation and differentiation of hematopoietic progenitors at the onset of blood specification. *Blood* 114: 4813–4822

Gkatzis K, Thalgott J, Dos-Santos-Luis D, Martin S, Lamandé N, Carette MF, Disch F, Snijder RJ, Westermann CJ, Mager JJ *et al* (2016) Interaction between ALK1 signaling and Connexin40 in the development of arteriovenous malformations. *Arterioscler Thromb Vasc Biol* 36: 707–717

González-Hernández S, Gómez MJ, Sánchez-Cabo F, Méndez-Ferrer S, Muñoz-Cánoves P, Isern J (2020) Sox17 controls emergence and remodeling of nestin-expressing coronary vessels. *Circ Res* 127: e252–e270

Grozdanov PN, Yovchev MI, Dabeva MD (2006) The oncofetal protein glypican-3 is a novel marker of hepatic progenitor/oval cells. *Lab Invest* 86: 1272–1284

Haefliger JA, Nicod P, Meda P (2004) Contribution of connexins to the function of the vascular wall. *Cardiovasc Res* 62: 345–356

He L, Tian X, Zhang H, Wythe J, Zhou B (2014) Fabp4-CreER lineage tracing revealstwo distinctive coronary vascular populations. *J Cell Mol Med* 18: 2152–2156

© 2023 The Authors

Hermkens DMA, van Impel A, Urasaki A, Bussmann J, Duckers HJ, Schulte-Merker S (2015) Sox7 controls arterial specification in conjunction with hey2 and efnb2 function. *Development* 142: 1695 – 1704

Hoang P, Huebsch N, Bang SH, Siemons BA, Conklin BR, Healy KE, Ma Z, Jacquir S (2018) Quantitatively characterizing drug-induced arrhythmic contractile motions of human stem cell-derived cardiomyocytes. *Biotechnol Bioeng* 115: 1958 – 1970

Hong N, Zhang E, Xie H, Jin L, Zhang Q, Lu Y, Chen AF, Yu Y, Zhou B, Chen S *et al* (2021) The transcription factor Sox7 modulates endocardiac cushion formation contributed to atrioventricular septal defect through Wnt4/Bmp2 signaling. *Cell Death Dis* 12: 393

Ingolia NT, Lareau LF, Weissman JS (2011) Ribosome profiling of mouse embryonic stem cells reveals the complexity and dynamics of mammalian proteomes. *Cell* 147: 789 – 802

Jankowska-Steifer E, Madej M, Niderla-Bielińska J, Ruminski S, Flaht-Zabost A, Czarnowska E, Gula G, Radomska-Leśniewska DM, Ratajska A (2015) Vasculogenic and hematopoietic cellular progenitors are scattered within the prenatal mouse heart. *Histochem Cell Biol* 143: 153 – 169

Jiang X, Li T, Li B, Wei W, Li F, Chen S, Xu R, Sun K (2021) SOX7 suppresses endothelial-to-mesenchymal transitions by enhancing VE-cadherin expression during outflow tract development. *Clin Sci* 135: 829 – 846

Kaminow B, Yunusov D, Dobin A (2021) STARsolo: accurate, fast and versatile mapping/quantification of single-cell and single-nucleus RNA-seq data. *bioRxiv* https://doi.org/10.1101/2021.05.05.442755 [PREPRINT]

Kanzler B, Kuschert SJ, Liu YH, Mallo M (1998) Hoxa-2 restricts the chondrogenic domain and inhibits bone formation during development of the branchial area. *Development* 125: 2587 – 2597

Karbassi E, Fenix A, Marchiano S, Muraoka N, Nakamura K, Yang X, Murry CE (2020) Cardiomyocyte maturation: advances in knowledge and implications for regenerative medicine. *Nat Rev Cardiol* 17: 341 – 359

Kataoka H, Hayashi M, Nakagawa R, Tanaka Y, Izumi N, Nishikawa S, Jakt ML, Tarui H, Nishikawa S-I (2011) Etv2/ER71 induces vascular mesoderm from Flk1$^+$PDGFRα^+ primitive mesoderm. *Blood* 118: 6975 – 6986

Kim K, Kim I-K, Yang JM, Lee E, Koh BI, Song S, Park J, Lee S, Choi C, Kim JW *et al* (2016) SOXF transcription factors are positive feedback regulators of VEGF signaling. *Circ Res* 121: 839 – 852

Kolde R, Laur S, Adler P, Vilo J (2012) Robust rank aggregation for gene list integration and meta-analysis. *Bioinformatics* 28: 573 – 580

Li J, Miao L, Shieh D, Spiotto E, Li J, Zhou B, Paul A, Schwartz RJ, Firulli AB, Singer HA *et al* (2016) Single-cell lineage tracing reveals that oriented cell division contributes to trabecular morphogenesis and regional specification. *Cell Rep* 15: 158 – 170

Lien CL, Wu C, Mercer B, Webb R, Richardson JA, Olson EN (1999) Control of early cardiac-specific transcription of Nkx2-5 by a GATA-dependent enhancer. *Development* 126: 75 – 84

Lilly AJ, Costa G, Largeot A, Fadlullah MZH, Lie-A-Ling M, Lacaud G, Kouskoff V (2016) Interplay between SOX7 and RUNX1 regulates hemogenic endothelial fate in the yolk sac. *Development* 143: 4341 – 4351

Lilly AJ, Mazan A, Scott DA, Lacaud G, Kouskoff V (2017) SOX7 expression is critically required in FLK1-expressing cells for vasculogenesis and angiogenesis during mouse embryonic development. *Mech Dev* 146: 31 – 41

Lim WF, Burdach J, Funnell APW, Pearson RCM, Quinlan KGR, Crossley M (2016) Directing an artificial zinc finger protein to new targets by fusion to a non-DNA-binding domain. *Nucleic Acids Res* 44: 3118 – 3130

Lincoln J, Alfieri CM, Yutzey KE (2004) Development of heart valve leaflets and supporting apparatus in chicken and mouse embryos. *Dev Dyn* 230: 239 – 250

Love MI, Huber W, Anders S (2014) Moderated estimation of fold change and dispersion for RNA-seq data with DESeq2. *Genome Biol* 15: 550

Metzis V, Courtney AD, Kerr MC, Ferguson C, Rondón Galeano MC, Parton RG, Wainwright BJ, Wicking C (2013) Patched1 is required in neural crest cells for the prevention of orofacial clefts. *Hum Mol Genet* 22: 5026 – 5035

Mills RJ, Titmarsh DM, Koenig X, Parker BL, Ryall JG, Quaife-Ryan GA, Voges HK, Hodson MP, Ferguson C, Drowley L *et al* (2017) Functional screening in human cardiac organoids reveals a metabolic mechanism for cardiomyocyte cell cycle arrest. *Proc Natl Acad Sci USA* 114: E8372 – E8381

Muzumdar MD, Tasic B, Miyamichi K, Li L, Luo L (2007) A global double-fluorescent Cre reporter mouse. *Genesis* 45: 593 – 605

Mysliwiec MR, Bresnick EH, Lee Y (2011) Endothelial Jarid2/Jumonji is required for normal cardiac development and proper Notch1 expression. *J Biol Chem* 286: 17193 – 17204

Nakano H, Liu X, Arshi A, Nakashima Y, van Handel B, Sasidharan R, Harmon AW, Shin JH, Schwartz RJ, Conway SJ *et al* (2013) Haemogenic endocardium contributes to transient definitive haematopoiesis. *Nat Commun* 4: 1564

Nelson TJ, Chiriac A, Faustino RS, Crespo-Diaz RJ, Behfar A, Terzic A (2009) Lineage specification of Flk-1$^+$ progenitors is associated with divergent Sox7 expression in cardiopoiesis. *Differentiation* 77: 248 – 255

Patel J, Seppanen EJ, Rodero MP, Wong HY, Donovan P, Neufeld Z, Fisk NM, Francois M, Khosrotehrani K (2017) Functional definition of progenitors versus mature endothelial cells reveals key SoxF-dependent differentiation process. *Circulation* 135: 786 – 805

Picelli S, Faridani OR, Björklund ÅK, Winberg G, Sagasser S, Sandberg R (2014) Full-length RNA-seq from single cells using Smart-seq2. *Nat Protoc* 9: 171 – 181

Pinto AR, Ilinykh A, Ivey MJ, Kuwabara JT, D'Antoni ML, Debuque R, Chandran A, Wang L, Arora K, Rosenthal NA *et al* (2016) Revisiting cardiac cellular composition. *Circ Res* 118: 400 – 409

Qu X, Harmelink C, Baldwin HS (2019) Tie2 regulates endocardial sprouting and myocardial trabeculation. *JCI Insight* 5: e96002

Red-Horse K, Ueno H, Weissman IL, Krasnow MA (2010) Coronary arteries form by developmental reprogramming of venous cells. *Nature* 464: 549 – 553

Rhee S, Paik DT, Yang JY, Nagelberg D, Williams I, Tian L, Roth R, Chandy M, Ban J, Belbachir N *et al* (2021) Endocardial/endothelial angiocrines regulate cardiomyocyte development and maturation and induce features of ventricular non-compaction. *Eur Heart J* 42: 4264 – 4276

Robinson MD, McCarthy DJ, Smyth GK (2010) edgeR: a Bioconductor package for differential expression analysis of digital gene expression data. *Bioinformatics* 26: 139 – 140

Saba R, Kitajima K, Rainbow L, Engert S, Uemura M, Ishida H, Kokkinopoulos I, Shintani Y, Miyagawa S, Kanai Y *et al* (2019) Endocardium differentiation through Sox17 expression in endocardium precursor cells regulates heart development in mice. *Sci Rep* 9: 11953

Sacilotto N, Monteiro R, Fritzsche M, Becker PW, Sanchez-del-Campo L, Liu K, Pinheiro P, Ratnayaka I, Davies B, Goding CR *et al* (2013) Analysis of Dll4 regulation reveals a combinatorial role for Sox and Notch in arterial development. *Proc Natl Acad Sci USA* 110: 11893 – 11898

Sakamoto Y, Hara K, Kanai-Azuma M, Matsui T, Miura Y, Tsunekawa N, Kurohmaru M, Saijoh Y, Koopman P, Kanai Y (2007) Redundant roles of Sox17 and Sox18 in early cardiovascular development of mouse embryos. *Biochem Biophys Res Commun* 360: 539 – 544

Sampath P, Pritchard DK, Pabon L, Reinecke H, Schwartz SM, Morris DR, Murry CE (2008) A hierarchical network controls protein translation

 © 2023 The Authors

during murine embryonic stem cell self-renewal and differentiation. *Cell Stem Cell* 2: 448–460

Sandireddy R, Cibi DM, Gupta P, Singh A, Tee N, Uemura A, Epstein JA, Singh MK (2019) Semaphorin 3E/PlexinD1 signaling is required for cardiac ventricular compaction. *JCI Insight* 4: e125908

Schmidt U, Weigert M, Broaddus C, Myers G (2018) Cell detection with star-convex polygons. In *Medical Image Computing and Computer Assisted Intervention–MICCAI 2018*, Frangi AF, Schnabel JA, Davatzikos C, Alberola-López C, Fichtinger G (eds), pp 265–273. Cham: Springer International Publishing

Sharma B, Ho L, Ford G, Chen H, Goldstone A, Woo Y, Quertermous T, Reversade B, Red-Horse K (2017) Alternative progenitor cells compensate to rebuild the coronary vasculature in Elabela-and Apj-deficient hearts. *Dev Cell* 42: 655–666

Shaw JP, Basch R, Shamamian P (2004) Hematopoietic stem cells and endothelial cell precursors express Tie-2, CD31 and CD45. *Blood Cells Mol Dis* 32: 168–175

Sim CB, Phipson B, Ziemann M, Rafehi H, Mills RJ, Watt KI, Abu-Bonsrah KD, Kalathur RKR, Voges HK, Dinh DT *et al* (2021) Sex-specific control of human heart maturation by the progesterone receptor. *Circulation* 143: 1614–1628

Simon AM, McWhorter AR (2002) Vascular abnormalities in mice lacking the endothelial gap junction proteins Connexin37 and Connexin40. *Dev Biol* 251: 206–220

Stuart T, Butler A, Hoffman P, Hafemeister C, Papalexi E, Mauck WM 3rd, Hao Y, Stoeckius M, Smibert P, Satija R (2019) Comprehensive integration of single-cell data. *Cell* 177: 1888–1902

Su X, Shi Y, Zou X, Lu Z-N, Xie G, Yang JYH, Wu C-C, Cui X-F, He K-Y, Luo Q *et al* (2017) Single-cell RNA-Seq analysis reveals dynamic trajectories during mouse liver development. *BMC Genomics* 18: 946

Su T, Stanley G, Sinha R, D'Amato G, Das S, Rhee S, Chang AH, Poduri A, Raftrey B, Dinh TT *et al* (2018) Single-cell analysis of early progenitor cells that build coronary arteries. *Nature* 559: 356–362

Trapnell C, Cacchiarelli D, Grimsby J, Pokharel P, Li S, Morse M, Lennon NJ, Livak KJ, Mikkelsen TS, Rinn JL (2014) The dynamics and regulators of cell fate decisions are revealed by pseudotemporal ordering of single cells. *Nat Biotechnol* 32: 381–386

Virágh S, Challice CE (1981) The origin of the epicardium and the embryonic myocardial circulation in the mouse. *Anat Rec* 201: 157–168

Wang Y, Nakayama M, Pitulescu ME, Schmidt TS, Bochenek ML, Sakakibara A, Adams S, Davy A, Deutsch U, Luthi U *et al* (2010) Ephrin-B2 controls VEGF-induced angiogenesis and lymphangiogenesis. *Nature* 465: 483–486

Wat MJ, Beck TF, Hernández-García A, Yu Z, Veenma D, Garcia M, Holder AM, Wat JJ, Chen Y, Mohila CA *et al* (2012) Mouse model reveals the role of SOX7 in the development of congenital diaphragmatic hernia associated with recurrent deletions of 8p23.1. *Hum Mol Genet* 21: 4115–4125

Wickham H (2009) *ggplot2: elegant graphics for data analysis*. New York: Springer Publishing Company, Incorporated

Wu B, Zhang Z, Lui W, Chen X, Wang Y, Chamberlain AA, Moreno-Rodriguez RA, Markwald RR, O'Rourke BP, Sharp DJ *et al* (2012) Endocardial cells form the coronary arteries by angiogenesis through myocardial-endocardial VEGF signaling. *Cell* 151: 1083–1096

Zamir L, Singh R, Nathan E, Patrick R, Yifa O, Yahalom-Ronen Y, Arraf AA, Schultheiss TM, Suo S, Han JJ *et al* (2017) Nkx2.5 marks angioblasts that contribute to hemogenic endothelium of the endocardium and dorsal aorta. *Elife* 6: e20994

Zhang C, Basta T, Klymkowsky MW (2005) Sox7 and Sox18 are essential for cardiogenesis in Xenopus. *Dev Dyn* 234: 878–891

Zhang H, Pu W, Li G, Huang X, He L, Tian X, Liu Q, Zhang L, Wu SM, Sucov HM *et al* (2016) Endocardium minimally contributes to coronary endothelium in the embryonic ventricular free walls. *Circ Res* 118: 1880–1893

License: This is an open access article under the terms of the Creative Commons Attribution License, which permits use, distribution and reproduction in any medium, provided the original work is properly cited.

Expanded View Figures

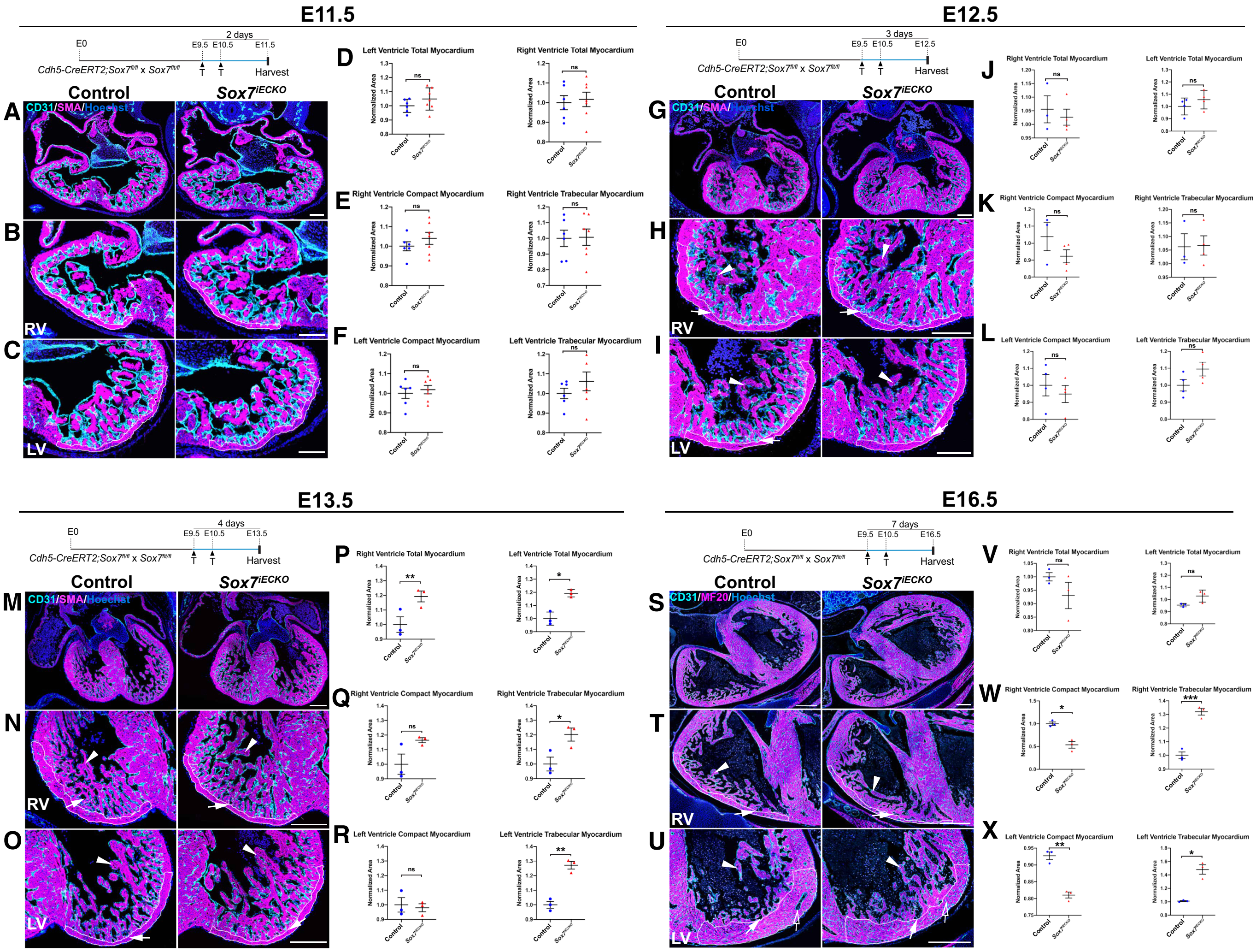

Figure EV1. Time-course morphological analysis of Sox7 endothelial loss-of-function embryos.

A–X (A–C, G–I, M–O, S–U) Morphological characterization of the cardiac defects present in *Sox7*^{iECKO} hearts compared to control hearts at E11.5 (A–C), E12.5 (G–I), E13.5 (M–O), and E16.5 (S–U) after Cre induction by tamoxifen injection at E9.5 and E10.5, by immunofluorescence with tissue-specific markers CD31 (endocardium/endothelium; cyan), MF20 (myocardium; magenta), and nuclei (Hoechst; blue). (A, G, M, S) Low-magnification images showing the general heart morphology. (B, C, H, I, N, O, T, U) High-magnification images showing the morphology of the compact (arrows) and trabecular (arrowheads) myocardium, and coronary arteries (empty arrow) in the right (RV; B, H, N, T) and left (LV; C, I, O, U) ventricles. White contours in high-magnification panels highlight the compact myocardium area. Morphological defects are evident from E13.5 (M–O) and maintained at E16.5 (S–U). (D–F, J–L, P–R, V–X) Quantification of total (D, J, P, V), compact and trabecular myocardium (E, F, K, L, Q, R, W, X) area in right (E, K, Q, W) and left (F, L, R, X) ventricular chambers of *Sox7*^{iECKO} hearts compared to control hearts. Quantification results confirmed no significant difference in tissue area at E11.5 (D–F) and E12.5 (J–L). It also identified a significant increase in total and trabecular myocardium area at E13.5 (P–R) and the maintenance of the E14.5 cardiac defects at E16.5 (V–X). Biological replicates as per the number of data points in the graphs. Mean ± SEM; *t*-test. $P < 0.05$ (*); $P < 0.005$ (**); $P < 0.0005$ (***); ns = not significant. Scale bars = 100 µm (A–C), 150 µm (G–I), 200 µm (M–O), and 300 µm (S–U).

 © 2023 The Authors

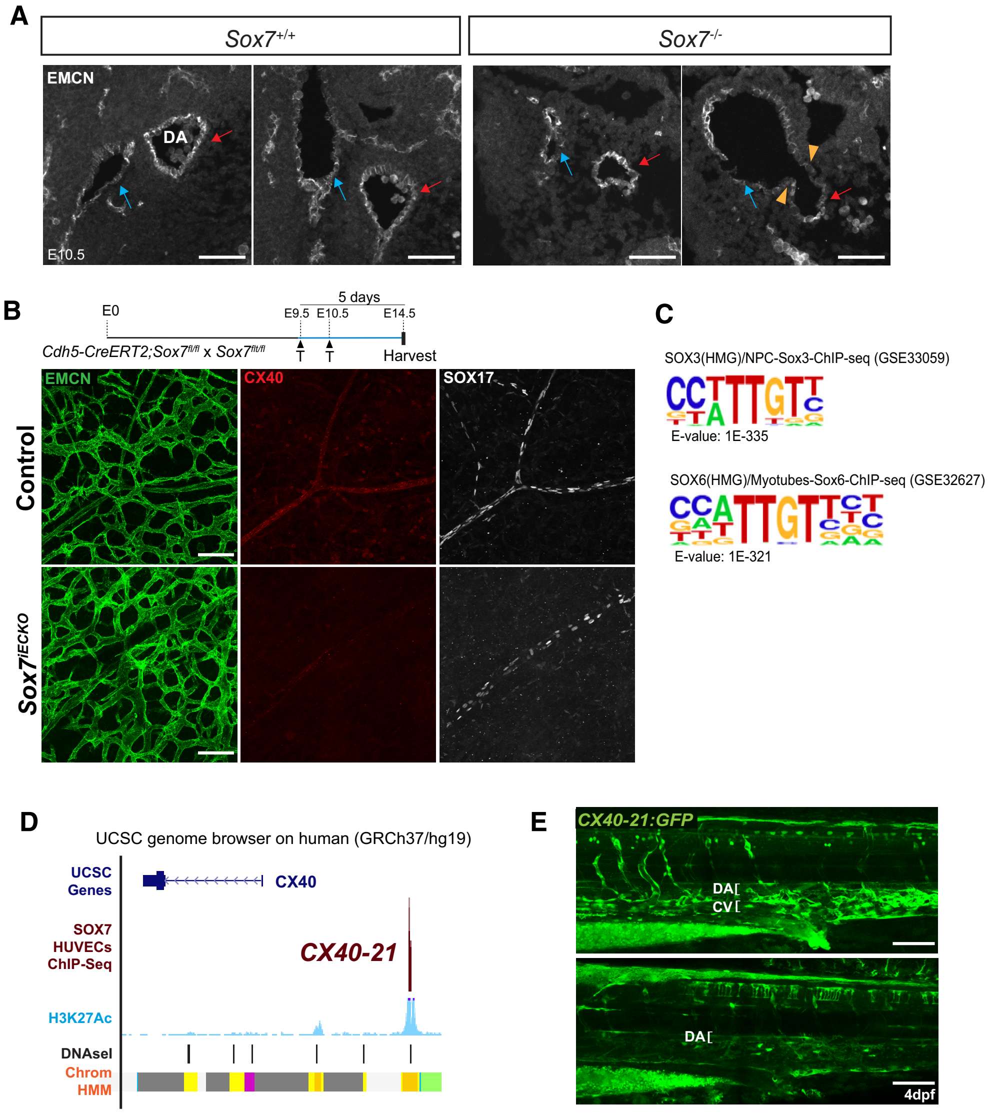

Figure EV2. SOX7 transcriptionally regulates the arterial specification marker, *Cx40*.

A Serial sections of a wild type and *Sox7*$^{-/-}$ hearts at E10.5, stained with endomucin (EMCN, white) to detect the endothelial lining. The dorsal aorta (DA) is labeled by red arrows, and the cardinal vein (CV) by blue arrows. Fusion between the DA and CV is indicated by orange arrowheads.

B Whole-mount immunostaining of *Sox7*^{iECKO} mutant and sibling control skin at E14.5, after Cre induction by tamoxifen injection at E9.5 and E10.5. The blood plexus is marked by endomucin (green), arteries by Cx40 (red), and SOX17 staining is shown in white.

C Schematic showing that SOX transcription motifs are the top binding motifs in SOX7 HUVECs ChIP-Seq data.

D Schematic representation of the human *CX40* locus showing a 500 bp putative regulatory element situated 21 kb upstream from the transcription start site (TSS) (denoted as *CX40-21* region) from UCSC Genome browser. The H3K27Ac is denoted in light blue, DNAseI hypersensitive hotspots are indicated by black/gray boxes, where the darkness is proportional to the maximum signal strength observed in any cell line. The chromatin state in HUVECs is shown in orange (indicates strong enhancer), green (weak transcribed), yellow (weak/poised enhancer), purple (inactive/poised promoter), and gray (polycomb repressed regions).

E The *CX40-21:GFP* transgene directs GFP fluorescence expression to vascular endothelium in transgenic zebrafish larvae at 4 dpf.

Data information: DA, dorsal aorta; CV, cardinal vein. Scale bars = 100 μm.

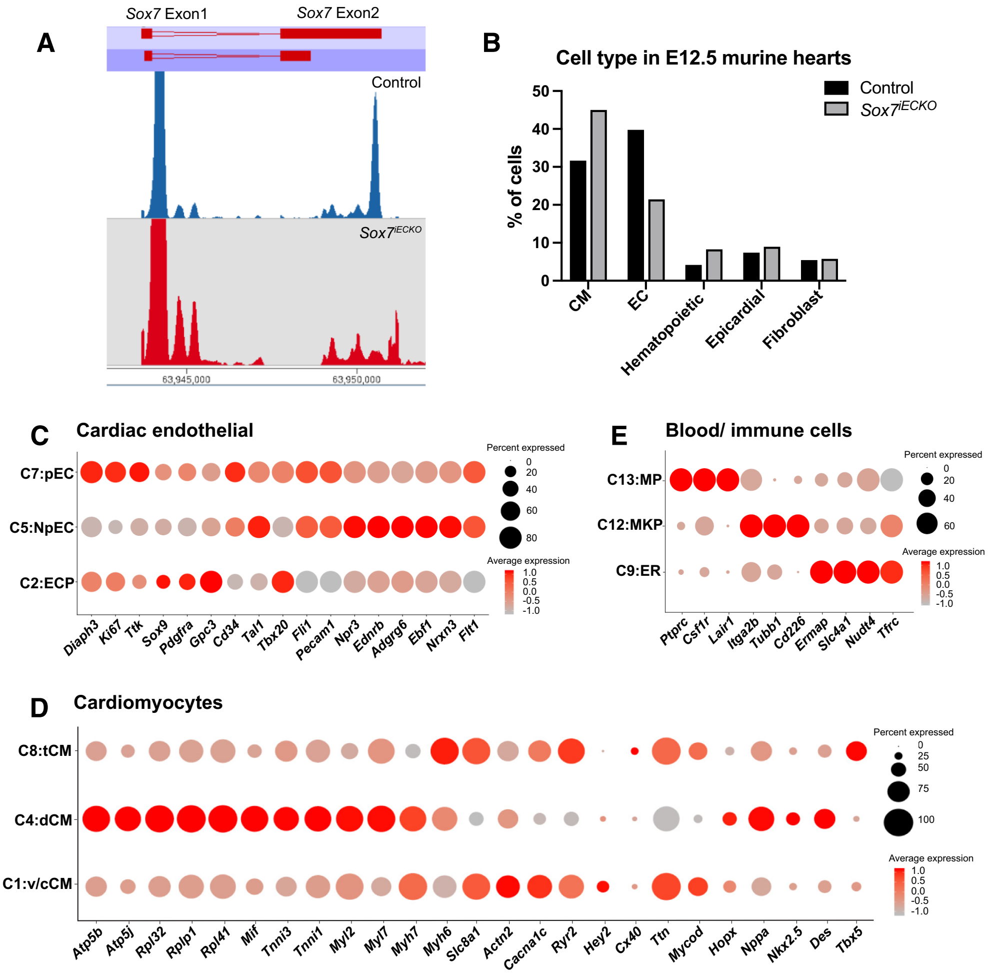

Figure EV3. Cell composition and marker gene expression profiles of cardiac populations in *Sox7^{iECKO}* hearts.

A There is a lack of transcripts harboring the exon 2 (floxed exon) of the *Sox7* locus in the *Sox7^{iECKO}* hearts (red track).

B Graphs showing the different cell types comprising E12.5 control and *Sox7^{iECKO}* hearts.

C–E Dot plots showing expression of genes that define each subcluster within major cell type population: cardiac endothelial cells (C), cardiomyocytes (D), and blood/immune cells (E).

 © 2023 The Authors

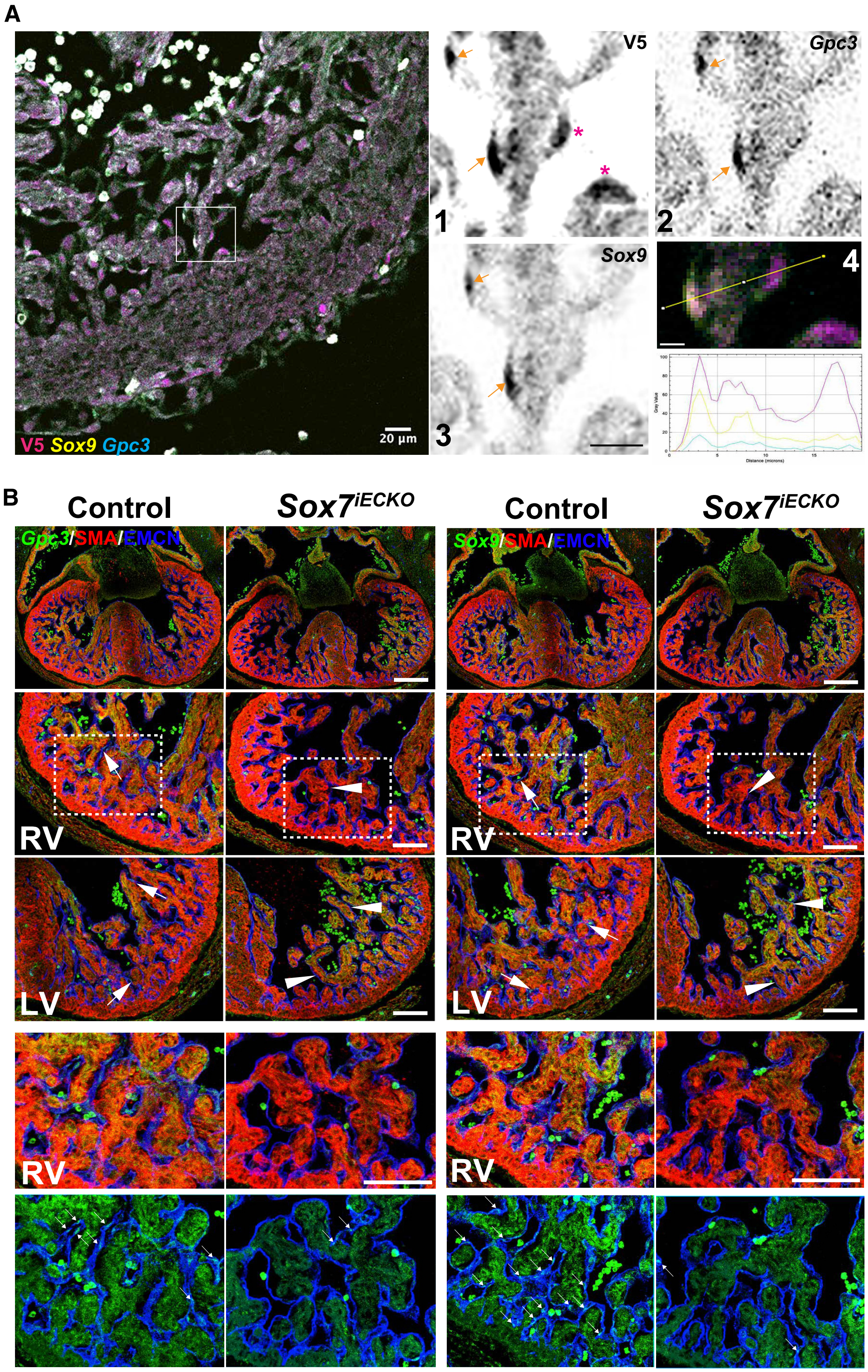
A
V5 Sox9 Gpc3
20 µm
V5
Gpc3
Sox9
1
2
3
4
B
Control
Sox7iECKO
Control
Sox7iECKO
Gpc3/SMA/EMCN
Sox9/SMA/EMCN
RV
LV
RV
LV
RV
RV

Figure EV4.

◀ **Figure EV4. Triple-positive Gpc3, Sox9, and Sox7 endocardial cells in 12.5 developing hearts are depleted in the Sox7^{iECKO}.**

A smFISH for *Gpc3* and *Sox9* combined with V5 immunofluorescence in *Sox7-V5* transgenic reporter embryos shows the presence of a subset of trabecular endocardial cells that are triple positive for SOX7, *Sox9*, and *Gpc3* (orange arrows). Pink asterisks, SOX7 single-positive cells. High magnification from inset with 1, 2, and 3 showing individual channels for each marker; 4 shows the plot profile of fluorophore distribution for triple- or single-positive cells. Scale bar in 3 and 10 µm.

B Expression pattern of C2 population markers. Analysis by smFISH of the C2 endocardial markers *Gpc3* (left panels), *Sox9* (right panels) in combination with immunofluorescence of endocardial (endomucin, blue), and myocardial (SMA, red) markers on tissue sections of control and *Sox7*^{iECKO} mutant hearts. The first row panels are low-magnification images showing the entire heart tissue. The second row panels show images of the right ventricles. The third row panels show the left ventricle. The two bottom row panels show a high magnification from inset in RV panels and reveal the loss of *Gpc3* and *Sox9* expression in the endocardium of the *Sox7*^{iECKO}. Markers (green), myocardium (red), and endocardium (blue). Scale bars = 200 µm (top panels), 100 µm (middle and bottom panels), arrows indicate *Gpc3*- and *Sox9*-positive endocardium in the control embryos; arrowheads indicate lack of expression of *Gpc3* and *Sox9* in the endocardium of *Sox7*^{iECKO} embryos.

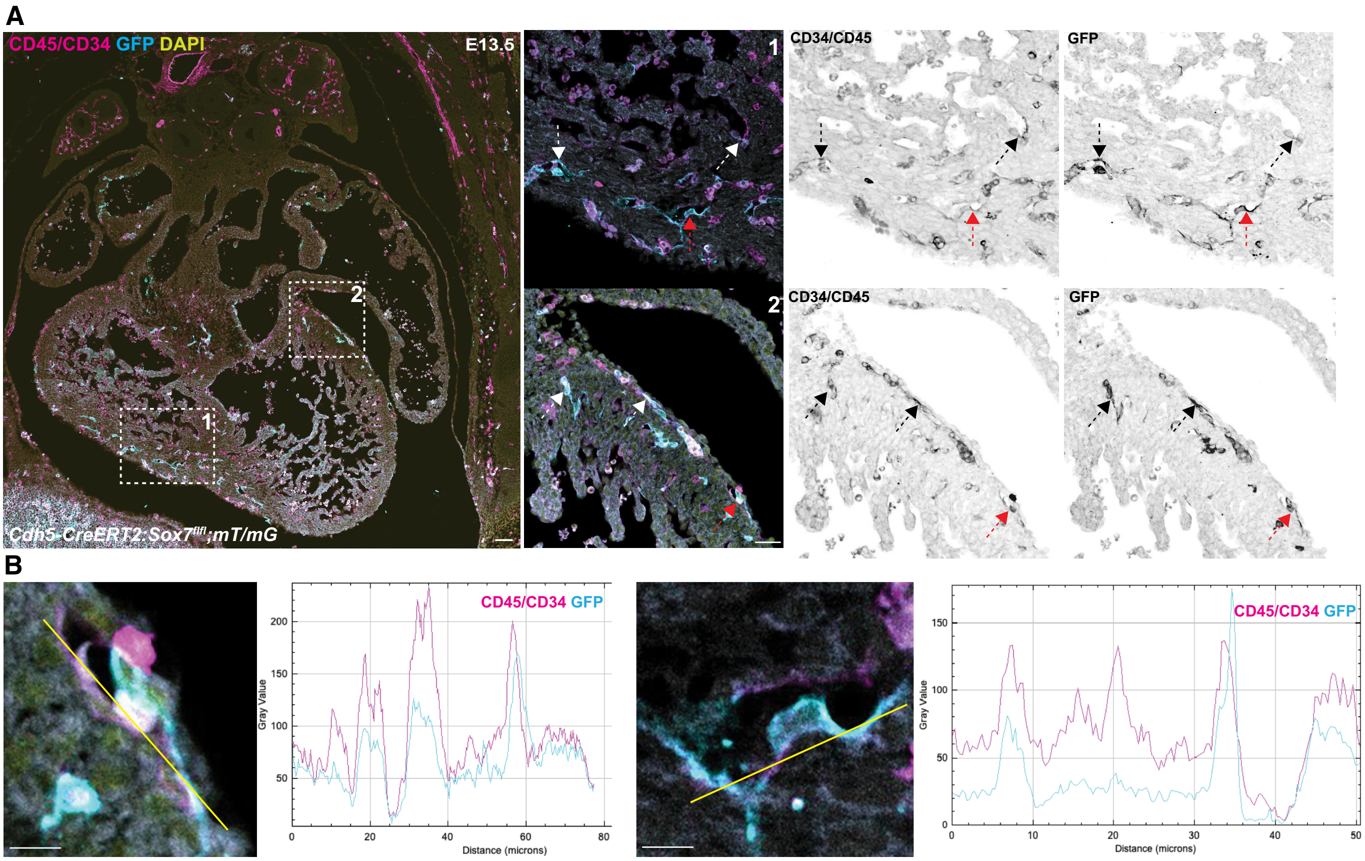

Figure EV5. Sox7-null endothelial cells transdifferentiate into hematopoietic cell lineages in vivo.

A Transverse section of an E13.5 heart from a triple transgenic *SOX7*^{iECKO}; *mT/mG* embryo stained for GFP, CD45/CD34 (same fluorophore), and DAPI. This staining shows the presence of GFP-positive endothelial cells in the coronary vasculature that express either CD34 or CD45 hematopoietic markers (red and black or white arrows). Scale bar = 20 µm.

B Plot profile analysis of a cell from the inner lining of a vessel showing the colocalization of the fluorescent signal. These regions are blown-up areas indicated by the red arrows. Scale bar = 10 µm.

 © 2023 The Authors

Appendix table of content

Figure legends S1 to S9……………………………………………………………………….2-4
Figures S1 to S9…………………………………………………………………………...5-13

Appendix figures legends

Appendix Figure S1. Time-course cell count and proliferation analysis of *Sox7* endothelial loss of function embryos.

(**A-C**) Proliferation analysis by Ki67 immunofluorescence on tissue sections from $Sox7^{iECKO}$ hearts (left panels) compared to control (right panels) at E12.5 (A-A''), E13.5 (B-B'') and E14.5 (C-C''). Images corresponding to low magnification showing the entire heart (A-C) and higher magnification showing the right (A'-C') and left (A''-C'') ventricles. Ki67 staining (red), nuclei (blue) and myocardial transparent mask (green). Scale bars = 300 µm (A,C), 200µm (B,C',C''), 150µm (A',A'') and 100µm (B',B'').

(**D-M**) Quantification of the number of cells forming the ventricular chamber (D, I), chamber myocardium (E, J), chamber endocardium (F, K), and the regional subdivision of the chamber endocardium into apical (G, L) and basal (H, M) endocardium at E12.5, E13.5 and E14.5 in $Sox7^{iECKO}$ hearts compared to control hearts. No significant changes were observed in these parameters and stages besides a significant decrease in endocardial cell numbers mainly in the basal endocardium (H, K, M).

(**N-W**) Quantification of the number of $Ki67^{+}$ proliferating cells in the ventricular chamber (N, S), chamber myocardium (O, T), chamber endocardium (P, U), and the regional subdivision of the chamber endocardium into apical (Q, V) and basal (R, W) endocardium at E12.5, E13.5 and E14.5 in $Sox7^{iECKO}$ hearts compared to control hearts. Results show reduced cell proliferation in the left chamber (N) and in the myocardium of both chambers (O, T) at E14.5, and a significant increase in cell proliferation in the apical endocardium at E13.5 (Q).

Data information: Biological replicates as per the number of data points in the graphs. Mean ± SEM; *t*-test. P <0.05 (*); P<0.005 (**); P<0.0005 (***); ns = not significant.

Appendix Figure S2. Whole-mount immunostaining of control and $Sox7^{iECKO}$ hearts at E14.5 after Cre induction by tamoxifen injection at E9.5, E10.5.

Coronary vessels are stained with VEGFR2 (green), coronary arteries (arrows) and trabecular myocardium (TM) are marked with CX40 (red). Asterisks indicate where the coronary arteries are lost in $Sox7^{iECKO}$ heart. Scale bars = 100 µm.

Appendix Figure S3. Morphometric quantification of epicardial and intramyocardial coronary vessel phenotype.

(A-D) (**A**, **C**) Immunofluorescence images similar to the ones showed in Fig. 2, highlighting the epicardial and coronary vessel phenotypes comparing Control (left panels) and $Sox7^{iECKO}$ (right panels) hearts at E14.5 (A) and E16.5 (C). Myocardium (MF20, magenta), endocardium (CD31, cyan), nuclei (Hoechst, blue). Arrows point at the intra-myocardial coronary vessels. Scale bars = 200µm (A) and 150µm (C). (**B**, **D**) Graphs showing the morphological quantifications of intramyocardial coronary vessel area (left), subepicardial area (middle) and subepicardial vessel area (right) at E14.5 (B) and E16.5 (D). Biological replicates as per the number of data points in the graphs. Mean ± SEM; *t*-test. P <0.05 (*); P<0.005 (**); P<0.0005 (***);P<0.0001 **(****)** ns = not significant.

Appendix Figure S4. Genome-wide transcriptomic analysis of *Sox7* loss of function conditions.

(A) Heatmap showing the gene expression profile from control and $Sox7^{\Delta ex2/\Delta ex2}$ whole-embryo RNA-Seq at E8.5. 15 genes were downregulated and 50 were upregulated in the mutant samples.

(B) Gene ontology analysis by DAVID bioinformatics database, showing the top biological processes associated to the differentially expressed genes in SOX7 E8.5 RNA-Seq data. The top 2 biological processes include blood vessel development (*Egfl7, Cdx4, Cx37, Cx40, Lama4*) and angiogenesis (*Egfl7, Flt4, Rapgef3, Tbx4, Dll4, Cx40, Mmrn2*).

(C) Heatmap showing the gene expression profile from RNA-Seq performed on sorted $CD31^{+}$ endothelial cells from E10.5 control and $Sox7^{iECKO}$ whole embryos, injected with tamoxifen at E9.5 to activate endothelial specific *Sox7* deletion.

Appendix Figure S 5. Expression pattern of C4 population markers.

(A-F) Expression pattern analysis by smFISH of the C4 myocardial markers Hopx (A-C) and Nppa (D-F) in combination with immunofluorescence of endocardial (endomucin) and myocardial (SMA) markers on tissues sections of Control (A-C) and $Sox7^{iECKO}$ mutant (D-F) hearts. Low magnification images showing the entire heart tissue (A-F). Marker (green), myocardium (red), endocardium (blue). Scale bars = 100 µm. The green panels show the signal for *Hopx* and *Nppa* only. Arrows point at the expression of the marker whereas arrowheads point at the lack of expression of the marker in control embryos at the trabecular base.

(G) Cell population of the compact myocardium (C1) showing the expression levels of *Mycn*, *Kcnd2* and *Hey2* from snRNA-Seq analysis. Comparison between control and $Sox7^{iECKO}$ shows a decrease in the number of cells expressing these 3 markers in the mutant embryos.

Appendix Figure S6. Pseudotime trajectory analysis of cells in control and $Sox7^{iECKO}$ hearts.

(A) Pseudotime analysis graphs of cells in all 13 identified clusters in control (left) and $Sox7^{iECKO}$ (right) hearts.

(B) Pseudotime analysis graphs of cells in all 12 identified populations in control (left) and $Sox7^{iECKO}$ (right) hearts, after removing the C2: Endocardial progenitors (ECP) population.

Appendix Figure S7. Lineage tracing analysis indicates that Sox7-null endothelial cells do not give rise to cardiomyocytes.

Immunofluorescence staining in transverse heart sections of $Sox7^{iECKO}$; *mT/mG* mutant and *Cre+; mT/mG* control embryos at E14.5, after injection with tamoxifen at E9.5 and E10.5. Blood vessels are stained with endomucin, EMCN (magenta), cells after Cre excision are shown by EGFP expression (green), and cardiomyocytes are marked by tropomyosin, TPM (blue). No EGFP/TPM double positive cells were found in the $Sox7^{iECKO}$. Scale bars = 100 µm.

Appendix Figure S8. Triple negative CD71$^-$CD45$^-$CD31$^-$ control cell population remains unchanged in Sox7 loss of function.

(A) Flow analysis of the CD71$^-$CD45$^-$CD31$^-$ control cell population, revealing that it remains unchanged in E13.5 *Sox7^{iECKO}* hearts relative to controls.

(B) Graphs showing the proportion of CD71$^-$CD45$^-$CD31$^-$ control population normalized to the CD71$^-$CD45$^{-/low}$ parent events.

Of note the panel shown here is duplicated from Figure 6B, to emphasise on a different cell population.

Data information: Biological replicates, n = 6 control hearts; n = 8 *Sox7^{iECKO}* hearts. Mean ± SEM; *t*-test, ns = not significant.

Appendix Figure S9 Multiplexed imaging method.

Top panel: Schematic depicting the serial sections and the different combinations of markers used to analyse the proliferative states of different cell types (eg either endothelial or cardiomyocytes).

Bottom panels: **(A)** Fluorescence image of left ventricle showing myocardium (SMA; magenta) and endocardium (CD31; cyan). **(B)** Tissue masks of myocardial and endocardial regions generated via intensity thresholding. **(C)** Myocardial mask with manually selected compact myocardial layer. **(D)** Layered myocardial mask depicting compact myocardium in addition to basal and apical trabecular layers, as determined from the compact layer selection. **(E)** Nuclear staining (Hoechst 33342). **(F)** Nuclear segmentation label generated by StarDist plugin in Fiji. **(G)** Ki67 immunofluorescence of cellular proliferation. **(H)** Classification of nuclei as either Ki67$^+$ (active; red) or Ki67$^-$ (blue) via average intensity thresholding. **(I)** Tissue classification of inactive/active myocardial nuclei. **(J)** Tissue classification of inactive/active endocardial nuclei.

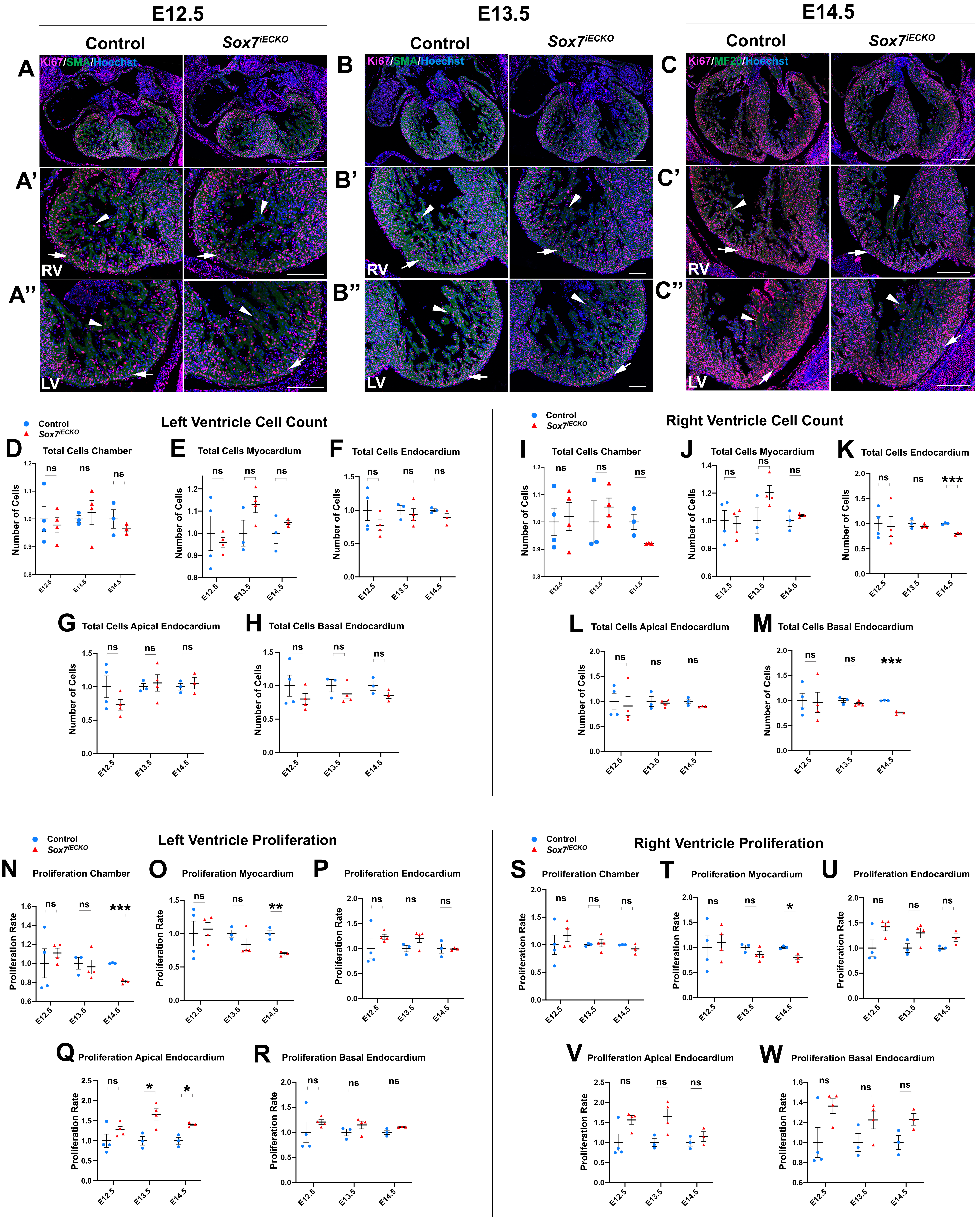

Appendix Fig. 1

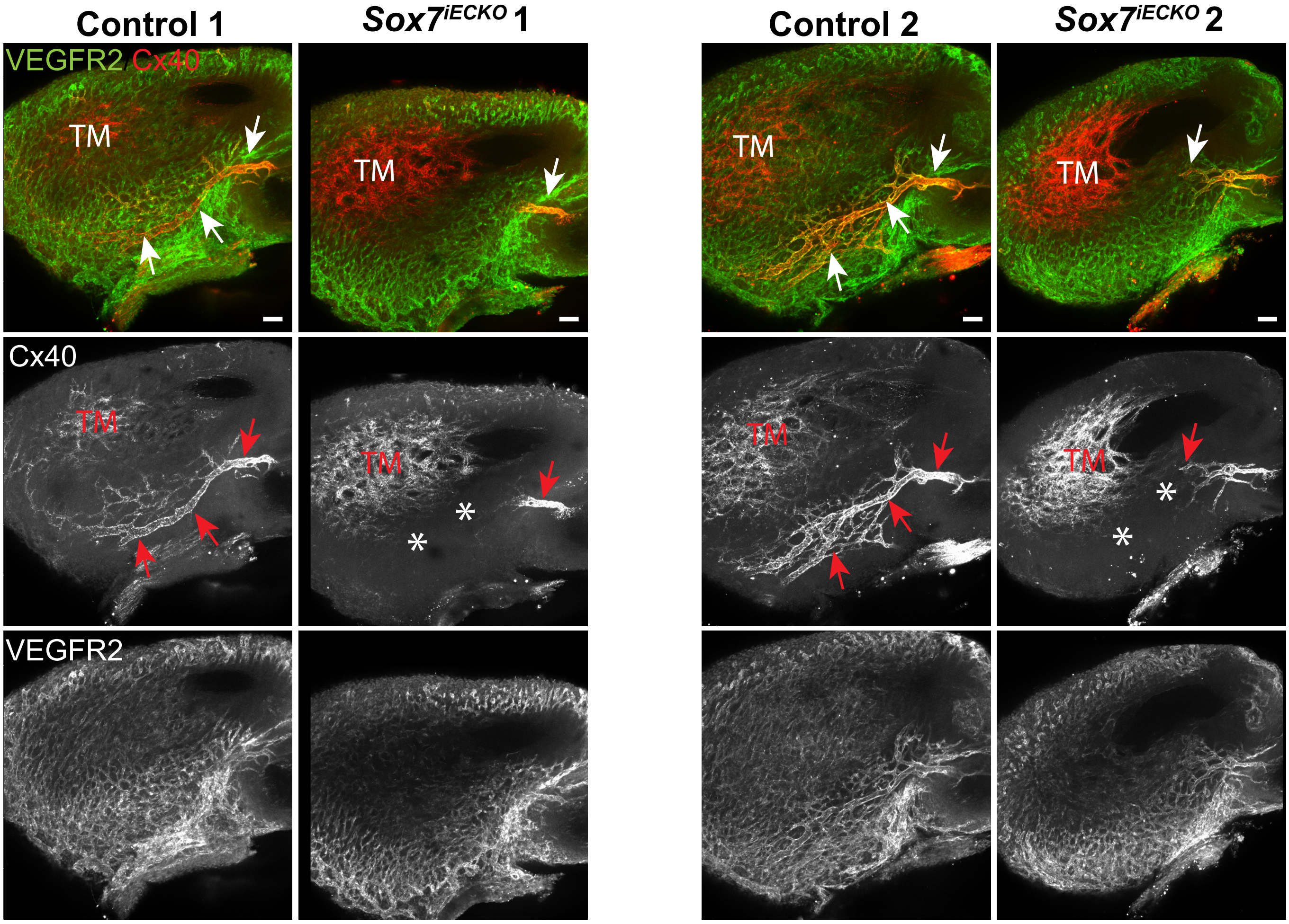

Appendix Fig.2

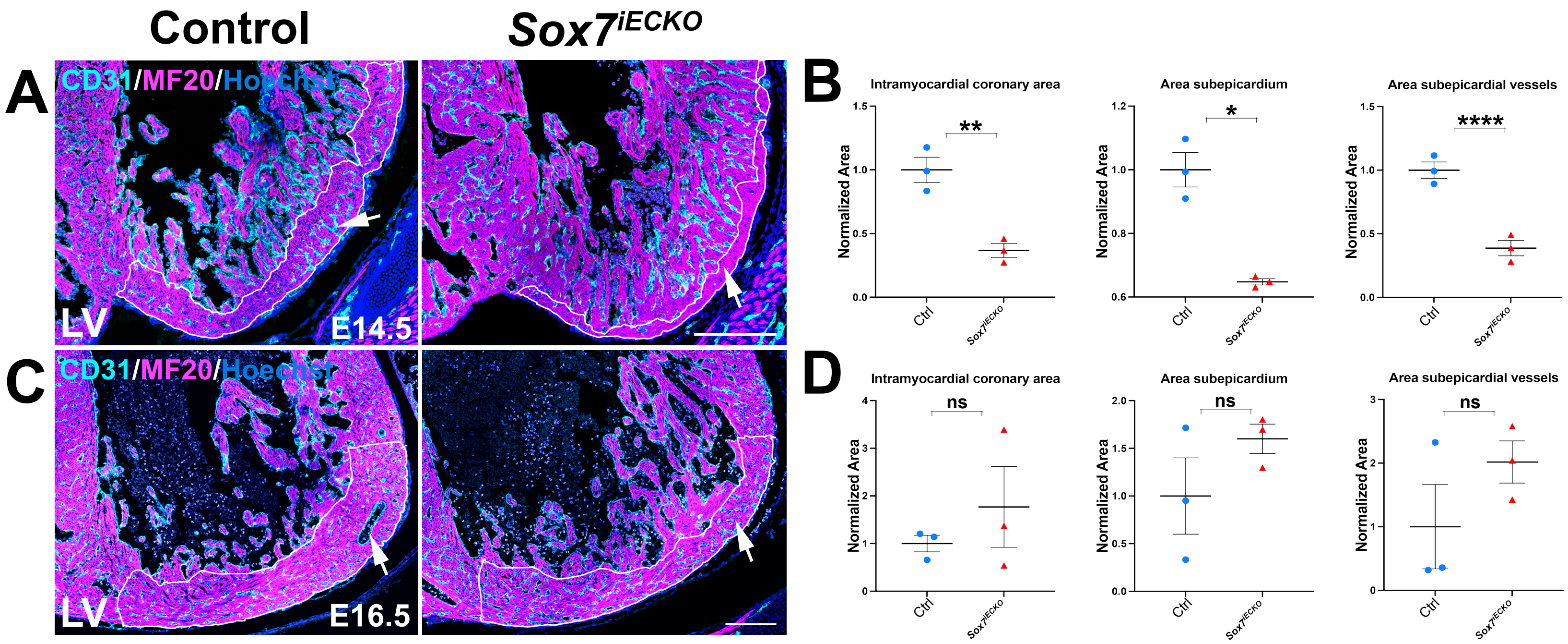

Appendix Fig.3

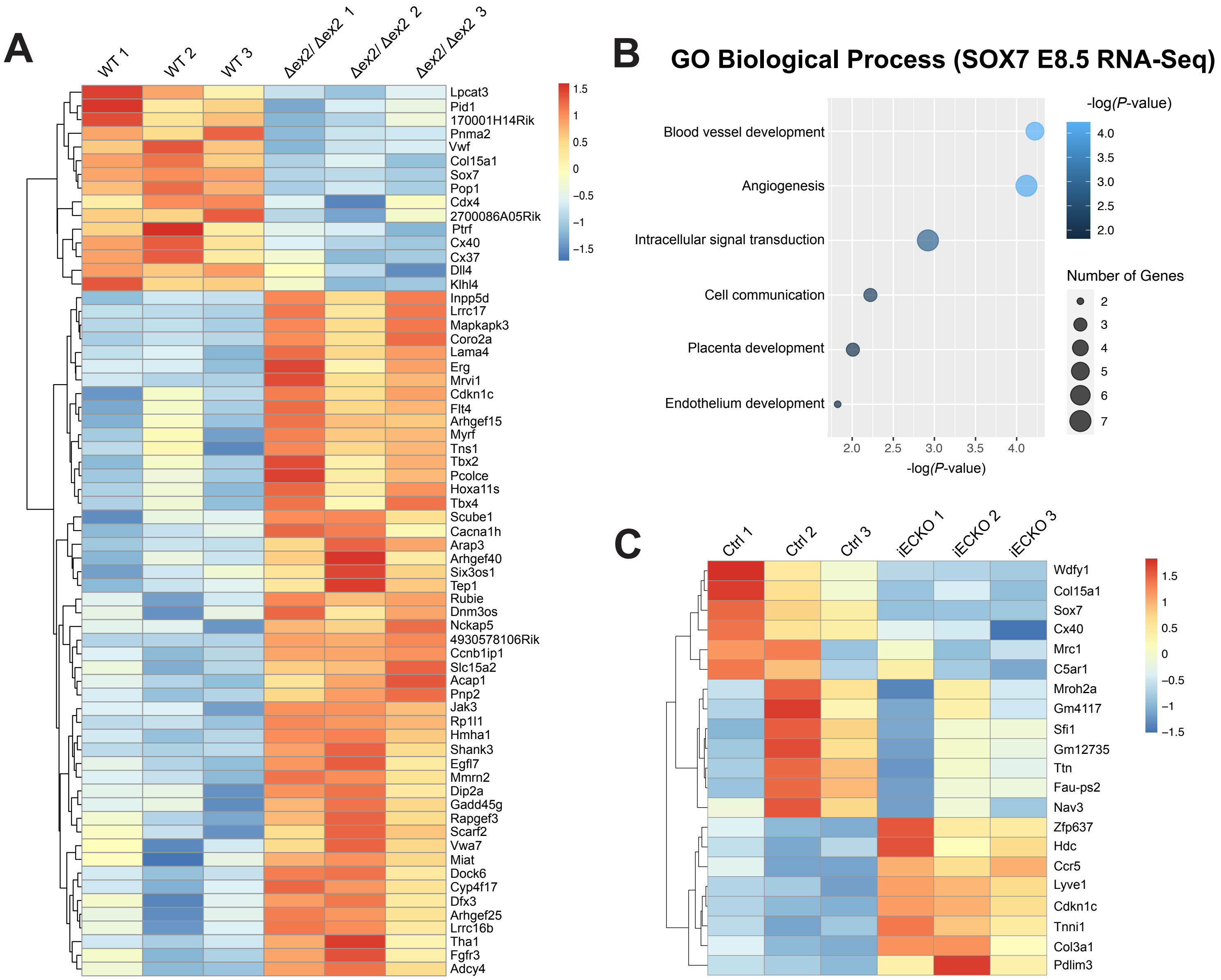

Appendix Fig.4

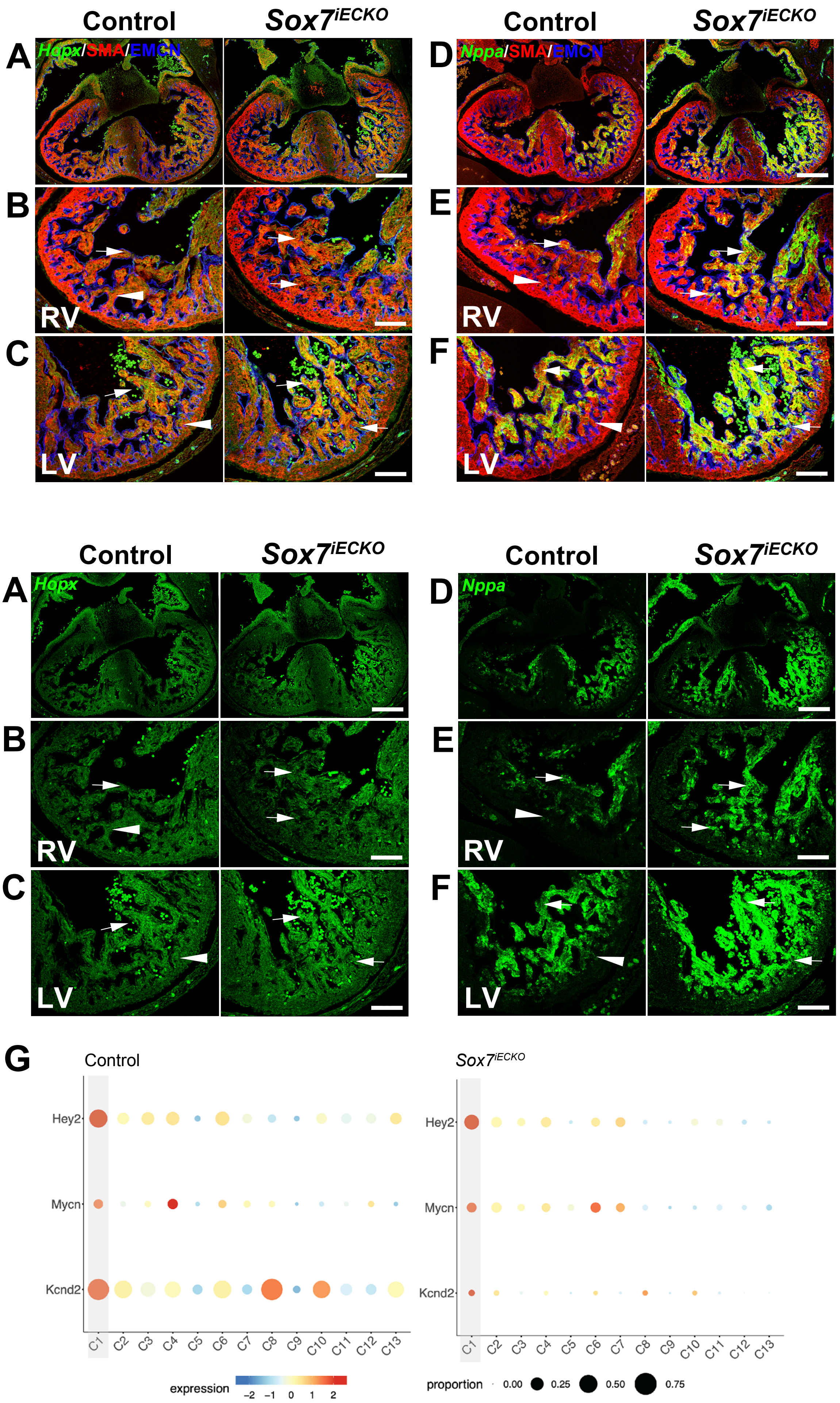

Appendix Fig. 5

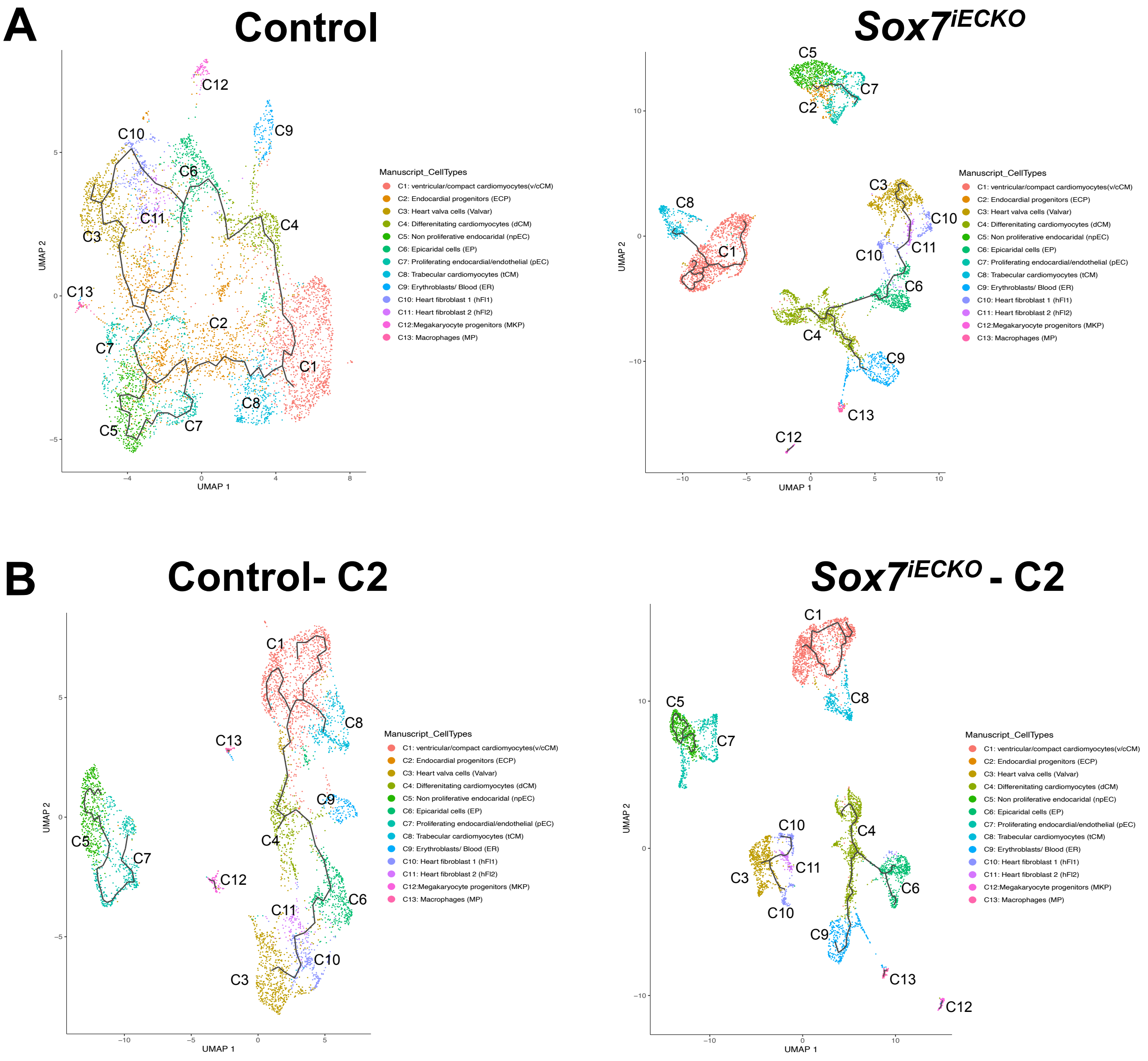

Appendix Fig.6

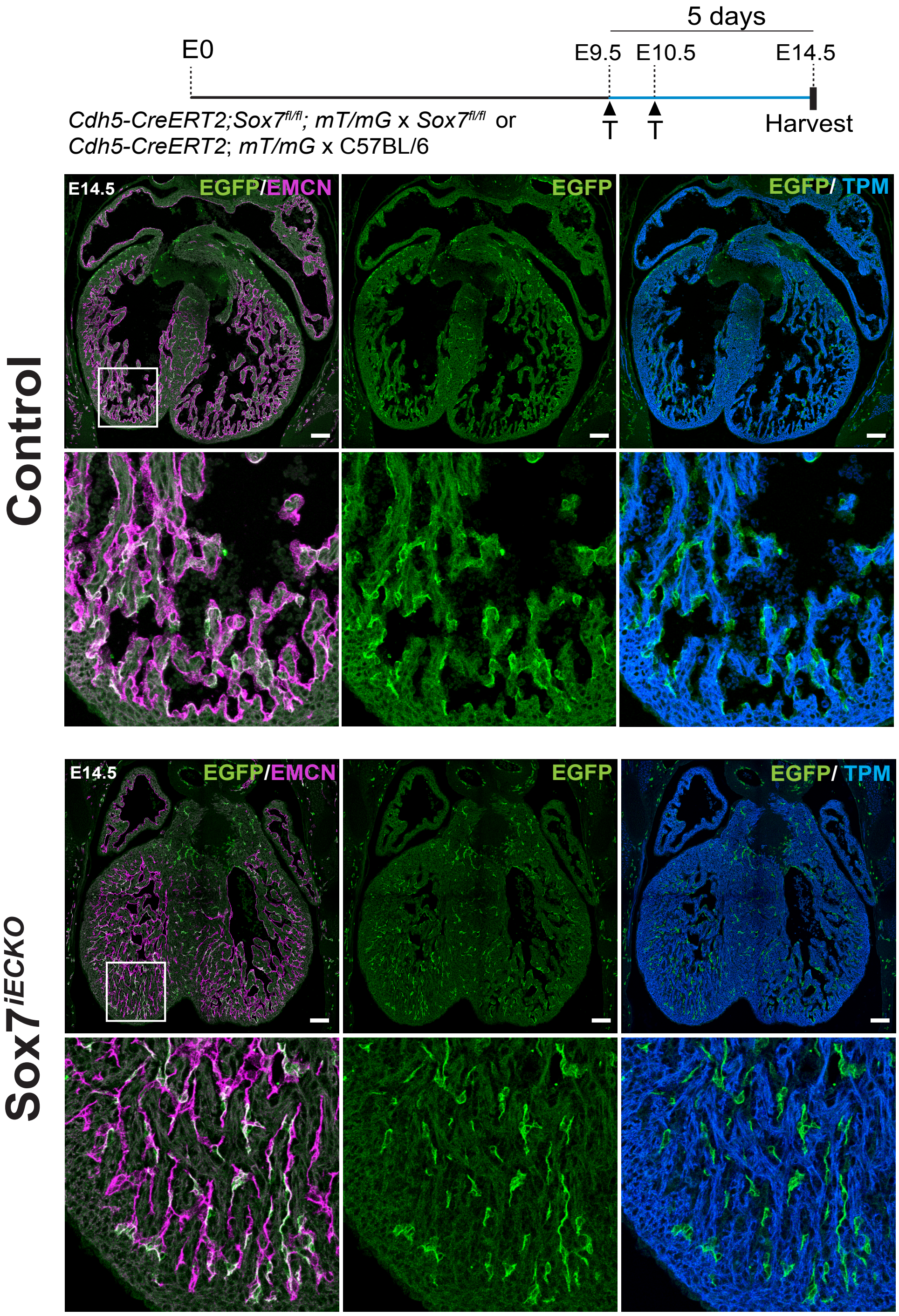

Appendix Fig.7

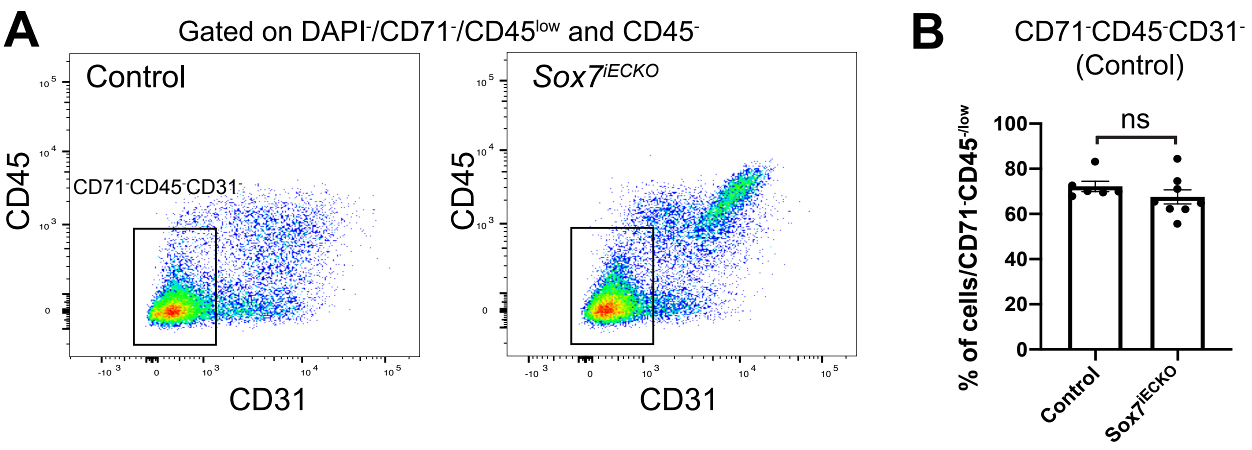

Appendix Fig.8

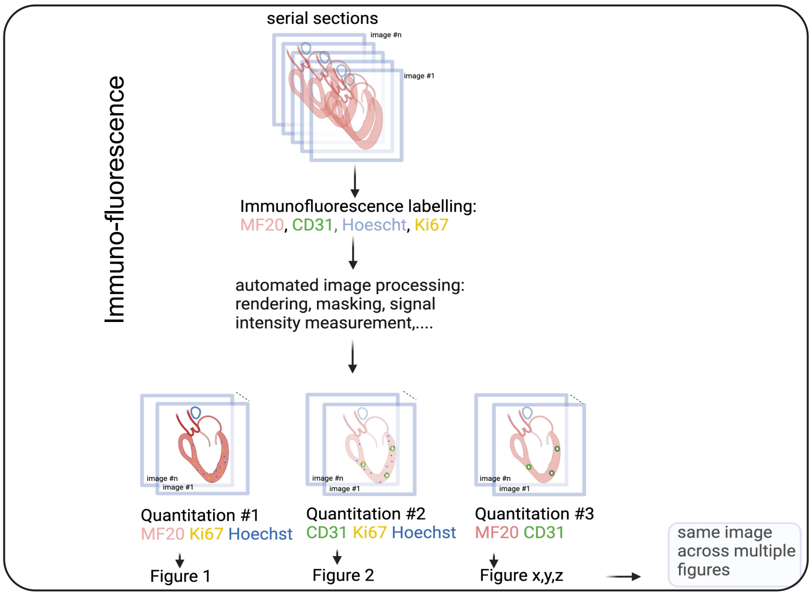

E11.5 - Left Ventricle

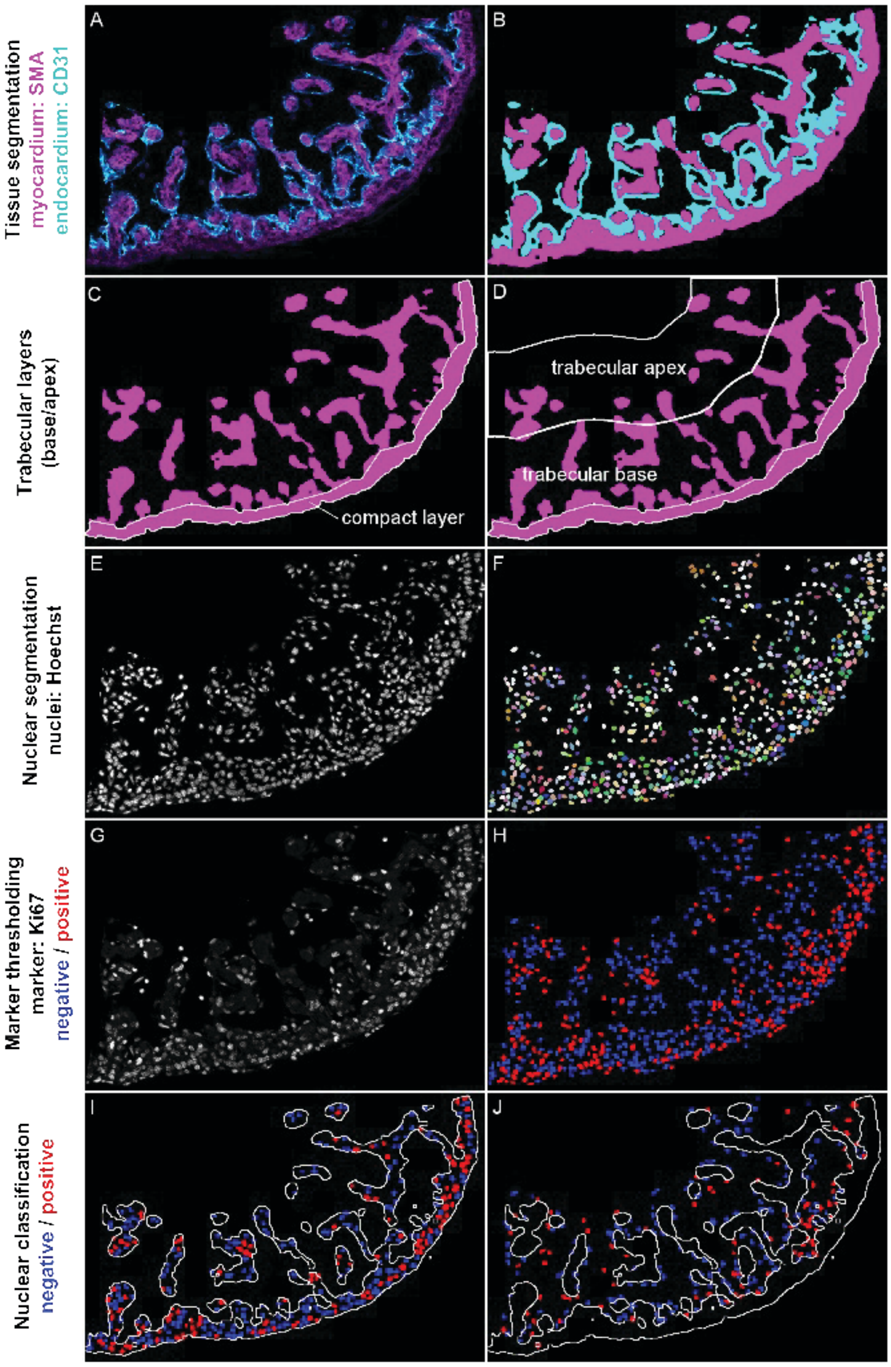

Appendix Fig.9
