# Supplementary material for: Sox7‐positive endothelial progenitors establish coronary arteries and govern ventricular compaction
Source: EMBO Rep. 2023 Aug 8;24(10):e55043. doi: 10.15252/embr.202255043 (PMC10561369; doi:10.15252/embr.202255043)
Supplement: Supplementary file 4 [file EMBR-24-e55043-s003.pdf]

EMBO reports

Sox7-positive endothelial progenitors establish coronary arteries and govern ventricular compaction

Ivy Chiang, David Humphrey, Richard Mills, Peter Kaltzis, Shikha Pachauri, Matthew Graus, Diptarka Saha, Zhijan Wu, Paul Young, Choon-Boon Sim, Tara Davidson, Andres Hernandez-Garcia, Chad Shaw, Alexander Renwick, Daryl Scott, Enzo Porrello, Emily Wong, James Hudson, Kristy Red-Horse, Gonzalo del Monte Nieto, and Mathias Francois
DOI: 10.15252/embr.202255043

Corresponding author(s): Mathias Francois (m.francois@centenary.org.au) , Gonzalo del Monte Nieto (gonzalo.delmontenieto@monash.edu)

Review Timeline:

Submission Date:	16th Mar 22
Editorial Decision:	21st Apr 22
Revision Received:	20th Apr 23
Editorial Decision:	16th Jun 23
Revision Received:	12th Jul 23
Accepted:	24th Jul 23

Editor: Martina Rembold/Deniz Senyilmaz-Tiebe

Transaction Report:

(Note: With the exception of the correction of typographical or spelling errors that could be a source of ambiguity, letters and reports are not edited. Depending on transfer agreements, referee reports obtained elsewhere may or may not be included in this compilation. Referee reports are anonymous unless the Referee chooses to sign their reports.)

Dear Prof. Francois,

Thank you for the submission of your research manuscript to our journal, which was now seen by two referees, whose reports are copied below.

We concur with the referees that the proposed role of Sox7 in cardiac development in principle very interesting. However, referees also raise some concerns that need to be addressed to consider publication here.

I find the reports informed and constructive, and believe that addressing the concerns raised will significantly strengthen the manuscript. As the reports are below, and I think all points need to be addressed, I will not detail them here.

Given these positive recommendations, we would like to invite you to revise your manuscript with the understanding that the referee concerns (as in their reports) must be fully addressed and their suggestions taken on board. Please address all referee concerns in a complete point-by-point response. Acceptance of the manuscript will depend on a positive outcome of a second round of review. It is EMBO reports policy to allow a single round of revision only and acceptance or rejection of the manuscript will therefore depend on the completeness of your responses included in the next, final version of the manuscript.

We generally allow three months as standard revision time. As a matter of policy, competing manuscripts published during this period will not negatively impact on our assessment of the conceptual advance presented by your study. However, we request that you contact the editor as soon as possible upon publication of any related work, to discuss how to proceed. Should you foresee a problem in meeting this three-month deadline, please let us know in advance and we may be able to grant an extension.

*** Temporary update to EMBO Press scooping protection policy:
We are aware that many laboratories cannot function at full efficiency during the current COVID-19/SARS-CoV-2 pandemic and have therefore extended our 'scooping protection policy' to cover the period required for a full revision to address the experimental issues highlighted in the editorial decision letter. Please contact the scientific editor handling your manuscript to discuss a revision plan should you need additional time, and also if you see a paper with related content published elsewhere.***

IMPORTANT NOTE: we perform an initial quality control of all revised manuscripts before re-review. Your manuscript will FAIL this control and the handling will be DELAYED if the following APPLIES:
1. A data availability section providing access to data deposited in public databases is missing (where applicable).
2. Your manuscript contains statistics and error bars based on n=2. Please use scatter plots in these cases.

You can submit the revision either as a Scientific Report or as a Research Article. For Scientific Reports, the revised manuscript can contain up to 5 main figures and 5 Expanded View figures. If the revision leads to a manuscript with more than 5 main figures it will be published as a Research Article. In this case the Results and Discussion section should be separate. If a Scientific Report is submitted, these sections have to be combined. This will help to shorten the manuscript text by eliminating some redundancy that is inevitable when discussing the same experiments twice. In either case, all materials and methods should be included in the main manuscript file

Supplementary/additional data: The Expanded View format, which will be displayed in the main HTML of the paper in a collapsible format, has replaced the Supplementary information. You can submit up to 5 images as Expanded View. Please follow the nomenclature Figure EV1, Figure EV2 etc. The figure legend for these should be included in the main manuscript document file in a section called Expanded View Figure Legends after the main Figure Legends section. Additional Supplementary material should be supplied as a single pdf labeled Appendix. The Appendix includes a table of content on the first page with page numbers, all figures and their legends. Please follow the nomenclature Appendix Figure Sx throughout the text and also label the figures according to this nomenclature. For more details please refer to our guide to authors.

Please note that for all articles published beginning 1 July 2020, the EMBO Reports reference style will change to the Harvard style for all article types. Details and examples are provided at
https://www.embopress.org/page/journal/14693178/authorguide#referencesformat

When submitting your revised manuscript, please carefully review the instructions that follow below. Failure to include requested items will delay the evaluation of your revision.

1) a .docx formatted version of the manuscript text (including legends for main figures, EV figures and tables). Please make sure that the changes are highlighted to be clearly visible.

2) individual production quality figure files as .eps, .tif, .jpg (one file per figure).

3) a .docx formatted letter INCLUDING the reviewers' reports and your detailed point-by-point responses to their comments. As

part of the EMBO Press transparent editorial process, the point-by-point response is part of the Review Process File (RPF), which will be published alongside your paper. For more details on our Transparent Editorial Process, please visit our website: https://www.embopress.org/page/journal/14693178/authorguide#transparentprocess
You are able to opt out of this by letting the editorial office know (emboreports@embo.org). If you do opt out, the Review Process File link will point to the following statement: "No Review Process File is available with this article, as the authors have chosen not to make the review process public in this case."

4) a complete author checklist, which you can download from our author guidelines (<http://embor.embopress.org/authorguide>). Please insert information in the checklist that is also reflected in the manuscript. The completed author checklist will also be part of the RPF.

5) Please note that all corresponding authors are required to supply an ORCID ID for their name upon submission of a revised manuscript (<https://orcid.org/>). Please find instructions on how to link your ORCID ID to your account in our manuscript tracking system in our Author guidelines (<http://embor.embopress.org/authorguide>).

6) We replaced Supplementary Information with Expanded View (EV) Figures and Tables that are collapsible/expandable online. A maximum of 5 EV Figures can be typeset. EV Figures should be cited as 'Figure EV1, Figure EV2" etc... in the text and their respective legends should be included in the main text after the legends of regular figures.

- For the figures that you do NOT wish to display as Expanded View figures, they should be bundled together with their legends in a single PDF file called *Appendix*, which should start with a short Table of Content. Appendix figures should be referred to in the main text as: "Appendix Figure S1, Appendix Figure S2" etc. See detailed instructions regarding expanded view here: <http://embor.embopress.org/authorguide#expandedview>.

- Additional Tables/Datasets should be labeled and referred to as Table EV1, Dataset EV1, etc. Legends have to be provided in a separate tab in case of .xls files. Alternatively, the legend can be supplied as a separate text file (README) and zipped together with the Table/Dataset file.

7) We would also encourage you to include the source data for figure panels that show essential data.

Numerical data should be provided as individual .xls or .csv files (including a tab describing the data). For blots or microscopy, uncropped images should be submitted (using a zip archive if multiple images need to be supplied for one panel). Additional information on source data and instruction on how to label the files are available <http://embor.embopress.org/authorguide#sourcedata>.

8) Our journal encourages inclusion of *data citations in the reference list* to directly cite datasets that were re-used and obtained from public databases. Data citations in the article text are distinct from normal bibliographical citations and should directly link to the database records from which the data can be accessed. In the main text, data citations are formatted as follows: "Data ref: Smith et al, 2001" or "Data ref: NCBI Sequence Read Archive PRJNA342805, 2017". In the Reference list, data citations must be labeled with "[DATASET]". A data reference must provide the database name, accession number/identifiers and a resolvable link to the landing page from which the data can be accessed at the end of the reference. Further instructions are available at <http://embor.embopress.org/authorguide#datacitation>.

9) Please make sure to include a Data Availability Section before submitting your revision - if it is not applicable, make a statement that no data were deposited in a public database. Primary datasets (and computer code, where appropriate) produced in this study need to be deposited in an appropriate public database (see <http://embor.embopress.org/authorguide#dataavailability>).

Please remember to provide a reviewer password if the datasets are not yet public.

The accession numbers and database should be listed in a formal "Data Availability " section (placed after Materials & Method) that follows the model below. Please note that the Data Availability Section is restricted to new primary data that are part of this study.

Data availability

The datasets (and computer code) produced in this study are available in the following databases:

- RNA-Seq data: Gene Expression Omnibus GSE46843 (https://www.ncbi.nlm.nih.gov/geo/query/acc.cgi?acc=GSE46843)
- [data type]: [name of the resource] [accession number/identifier/doi] ([URL or identifiers.org/DATABASE:ACCESSION])

*** Note - All links should resolve to a page where the data can be accessed. ***

10) Regarding data quantification, please ensure to specify the name of the statistical test used to generate error bars and P values, the number (n) of independent experiments underlying each data point (not replicate measures of one sample), and the test used to calculate p-values in each figure legend. Discussion of statistical methodology can be reported in the materials and methods section, but figure legends should contain a basic description of n, P and the test applied.
Please note that error bars and statistical comparisons may only be applied to data obtained from at least three independent biological replicates.
Please also include scale bars in all microscopy images.

We would also welcome the submission of cover suggestions, or motifs to be used by our Graphics Illustrator in designing a cover.

11) The journal requires a statement specifying whether or not authors have competing interests (defined as all potential or actual interests that could be perceived to influence the presentation or interpretation of an article). In case of competing interests, this must be specified in your disclosure statement. Further information: https://www.embopress.org/competing-interests

I look forward to seeing a revised version of your manuscript when it is ready. Please let me know if you have questions or comments regarding the revision.

Kind regards,

Deniz Senyilmaz Tiebe

Deniz Senyilmaz Tiebe, PhD
Editor
EMBO Reports

Referee #1:

This manuscript describes the effect of Sox7 deletion in endothelial cells on heart development. Null embryos show defects in coronary assembly at plexus level and i presence of excessive trabecular myocardium. By exploiting snRNAseq the authors demonstrate that cell specific Sox7 deletion causes reduction of a subset of cardiac endothelial cluster with stemness feature and an enrichment of cardiomyocyte subpopulations and influences the dynamics within the cardiomyocyte sub-populations Furthermore they demonstrate in an organoid system that Sox7 deletion in EC modifies physiological parameters of cardiomyocytes. The molecular analysis as well as the phenotype description are outstanding, but the present version lacks mechanistic insights (see comments on Fig 4 and 7)

CRITICISMS

Fig 2. In the result section, the authors describe the method to count the cell number. However this analysis has to be better explained and detailed in method section. Furthermore Ki67 staining used to define the proliferation index should be shown .
Fig 2. Is EC Sox7 deletion lethal?
Fig 2C. Morphometric analysis of coronary vessel reduction is required

Fig 3. I suggest to add PECAM staining in the analysis of coronary vessels. It is a more preferable EC marker than VEGFR2

Which is the effect of Sox7 deletion on subepicardial epicardium?

Figure 4 addresses the effect of Sox7 deletion on coronary formation and identify some putative targets by exploiting EC isolated from mice mutants and in vitro in Sox7-silenced HUAEC
The following new experiments are important: 1) the analysis of direct transcriptional activity of SOX7 on CX40 and CX37 ChiP and by luciferase reporter assays in HUEAC; 2) the analysis of Sox7-silenced HUAEC in term of activation (in vitro sprouting activity, proliferation , migration, ephrin B expression); 3) the ability of CX40, CX37, Dll4 to rescue the observed phenotype.

The organoid experiment represent an invaluable tool to understand the circuits between EC and cardiomyocytes. The current version of this strategy just directly confirm the in vivo analysis. I suggest to perform a. bulk RNAseq on the two separated populations or a secretome analysis by exploiting protein arrays

Referee #2:

In this article Chiang et al report that the deletion of Sox7 from endothelial cells results in defective development of coronary blood vessels, cardiomyocytes and hematopoietic cells. They have used many sophisticated approaches including snRNA-seq, bulk RNA-seq, and zebrafish transgenics. The manuscript is also very well written, and I enjoyed reading it. Nevertheless, the main point of the paper i.e., endothelial cells regulate the development of cardiomyocytes is somewhat insufficiently explored.

Major concerns

1. It is pretty clear that Sox7 regulates angiogenesis. Based on previous reports this is not surprising. Using snRNA-seq Chiang et al have identified some intriguing heterogeneity in the ECs that are affected by the loss of Sox7. The C2 cluster appear to be specifically reduced in the mutants. It will be extremely important to determine where these cells are located in vivo. Please select a few specific markers from the C2 cluster and perform IHC or in situ hybridization in control and mutant embryos.
2. The authors demonstrated by bulk RNA-seq that DLL4, Cx40 and Cx37 are reduced in embryos lacking Sox7. Are these genes specifically expressed in the C2 cluster? If not, please explain/discuss the meaning of such a finding.
3. The C4 cluster of cardiomyocytes appear to be enriched in mutants. As mentioned above, please select a few specific markers and show that these cells correspond to the trabecular cells as hypothesized.
4. I am also curious to know the cluster in which the compact layer cells are located. Based on the results one would predict that these cells are reduced in the mutants.
5. The changes in the trabecular and compact layers are very hard to appreciate. They appear comparable to me. The authors should try to explain this analysis better, may be with a schematic, and demonstrate convincingly that there are indeed some changes.
6. The zebrafish Cx40 transgenics is elegant. However, it will be more helpful to show that the expression of this reporter is reduced or absent in zebrafish embryos treated with Sox7 MO. If not, please explain the result.
7. Does lineage tracing support the argument that Sox7-/- endocardial cells transdifferentiate into hematopoietic cells? If not, please explain the result.

Minor concerns

1. Page 23: "non-statistically significant" should probably be "statistically non-significant".
2. Page 32: What are monocle algorithm and pseudo time analysis? Please explain these approaches in a manner that common readers can understand.

Chiang et al. Response to Reviewers

Referee #1:

This manuscript describes the effect of Sox7 deletion in endothelial cells on heart development. Null embryos show defects in coronary assembly at plexus level and i presence of excessive trabecular myocardium. By exploiting snRNAseq the authors demonstrate that cell specific Sox7 deletion causes reduction of a subset of cardiac endothelial cluster with stemness feature and an enrichment of cardiomyocyte subpopulations and influences the dynamics within the cardiomyocyte sub-populations Furthermore they demonstrate in an organoid system that Sox7 deletion in EC modifies physiological parameters of cardiomyocytes. The molecular analysis as well as the phenotype description are outstanding, but the present version lacks mechanistic insights (see comments on Fig. 4 and 7).

1- Fig 2. In the result section, the authors describe the method to count the cell number. However, this analysis has to be better explained and detailed in method section. Furthermore, Ki67 staining used to define the proliferation index should be shown.

We thank the reviewer for the suggestions to improve the manuscript. Following these suggestions, we have now modified the description of the quantification method used in the study. We have also generated a methods figure illustrating the different steps followed during these quantifications. For now, this is a figure for reviewer only (Fig. 1 for reviewer) but if assessed necessary, this could be integrated into the manuscript.

In addition, we have now included the images corresponding to the Ki67 immunofluorescence analysis on tissue sections from *Sox7*^{iECKO} hearts compared to control at E12.5, E13.5 and E14.5 (Sup. Fig. 2). Below are the details of what has been included in the new version of the manuscript.

New Methods section:

Quantification and data analysis

Image analysis to perform tissue area, quantification of cell number and actively proliferating cells was performed in Fiji. Image channels were isolated and overlaid with a mask for area quantification and nuclear classification performed via intensity thresholding. We generated endocardial, myocardial, and epicardial masks. Myocardial masks were further segmented into trabecular and compact myocardial masks by manual selection of the compact layer boundary following endocardial touchdowns as described in (Del Monte-Nieto et al., 2018). The luminal boundary of the trabecular myocardium and endocardium was found by iteratively expanding this compact layer selection in a pixelwise manner until it encompassed the entirety of the tissue; the distance at which all tissue was enclosed was labelled as the luminal apical boundary, with the basal/apical boundary defined as halfway between the compact myocardium and the apex of the trabeculae. This allowed further sub-segmentation of the endocardial and trabecular myocardial masks into basal and apical layers. Nuclear segmentation was performed with the StarDist plugin in Fiji (Schmidt et al., 2018). Individualised nuclei were subsequently classified using the previously generated tissue masks. This identified nuclei from cells forming the compact myocardium, basal and apical trabecular myocardium, epicardium, and basal and apical endocardium. The average intensity

of Ki67 staining within each nucleus was then measured, and nuclei further classified as either Ki67$^+$ or Ki67$^-$ based on a calibrated threshold; positive nuclei were labelled red and negative blue. The data collected was scaled to the characteristic length of the specific tissue area analyzed and normalized to the control value. Once scaled and normalized, data was plotted using GraphPad Prism. Data were analyzed for normal distribution using Shapiro-Wilk test. Once confirmed, statistical analysis was performed using parametric Student's *t*-test with significance achieved when p-value was <0.05. Data was represented in the graph as mean +/- SEM.

Methods Figure

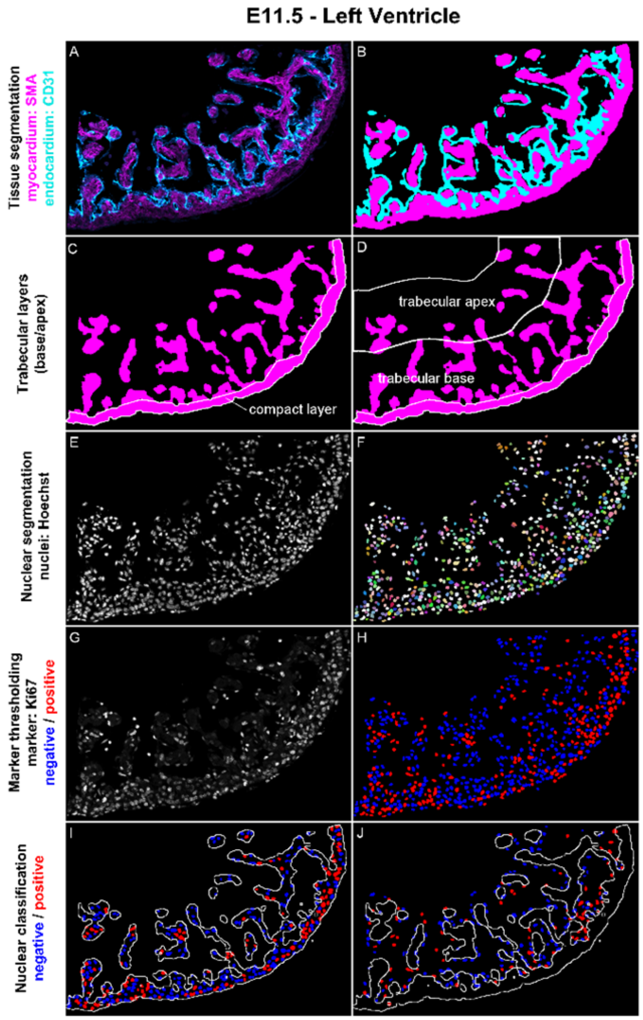

Figure 1 for reviewer only. Image processing pipeline.

(A) Fluorescence image of left ventricle showing myocardium (SMA; magenta) and endocardium (CD31; cyan). (B) Tissue masks of myocardial and endocardial regions generated via intensity thresholding. (C) Myocardial mask with manually selected compact myocardial layer. (D) Layered myocardial mask depicting compact myocardium in addition to basal and apical trabecular layers, as determined from the compact layer selection. (E) Nuclear staining (Hoechst 33342). (F) Nuclear segmentation label generated by StarDist plugin in Fiji. (G) Ki67 immunofluorescence of cellular proliferation. (H) Classification of nuclei as either Ki67$^+$ (active; red) or Ki67$^-$ (blue) via average intensity thresholding. (I) Tissue classification of inactive/active myocardial nuclei. (J) Tissue classification of inactive/active endocardial nuclei.

Ki67 images: These new IF panels have been integrated into the Sup. Fig. 2. Please see full Sup. Fig. 2 in the manuscript.

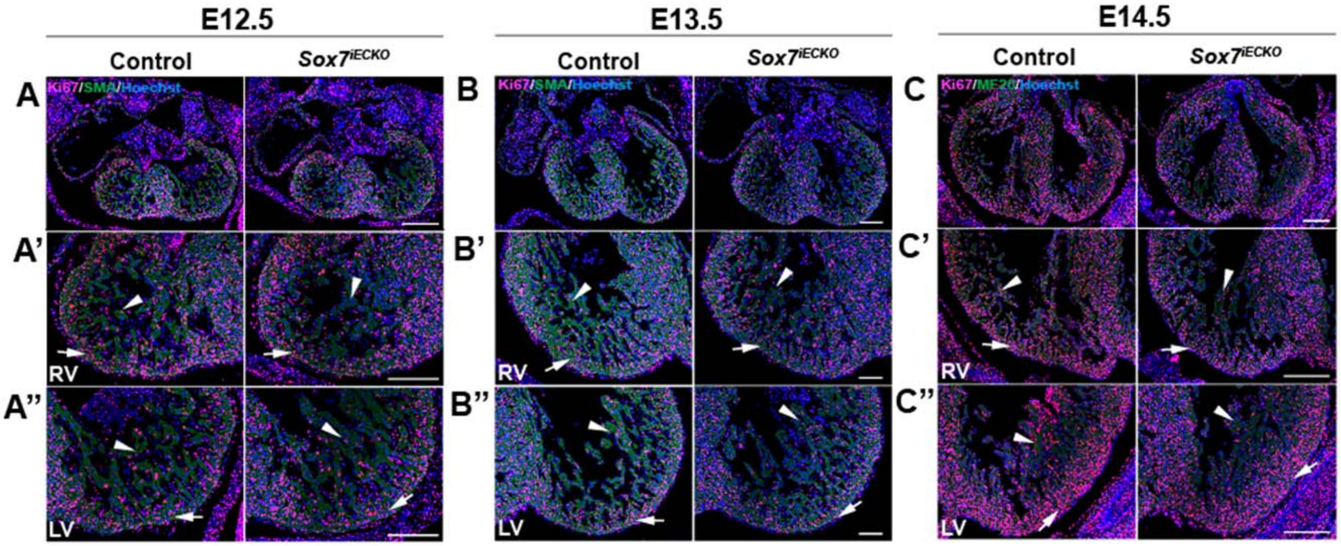

The corresponding figure legend has been updated accordingly as described below:

Supplementary Figure 2. Time-course cell count and proliferation analysis of *Sox7* endothelial loss of function embryos. (A-C'') Proliferation analysis by Ki67 immunofluorescence on tissue sections from *Sox7*^{iECKO} hearts (left panels) compared to control (right panels) at E12.5 (A-A''), E13.5 (B-B'') and E14.5 (C-C''). Images corresponding to low magnification showing the entire heart (A-C) and higher magnification showing the right (A'-C') and left (A''-C'') ventricles. Ki67 staining (red), nuclei (blue) and myocardial transparent mask (green). Scale bars = 300 µm (A, C), 200 µm (B, C', C''), 150 µm (A', A'') and 100 µm (B', B'').

2- Fig 2. Is EC Sox7 deletion lethal?

Yes, that is correct, we have recently reported the complete characterisation of the lymphatic vascular phenotype and embryonic lethality when *Sox7* gene is conditionally deleted in the endothelium compartment (Chiang et al., 2023).

3- Fig 2C. Morphometric analysis of coronary vessel reduction is required

In order to respond to questions 3 and 5 from Referee 1, we have performed a quantitative analysis based on CD31-staining of the coronary vessels area present within the compact myocardium region (intramyocardial coronaries), or present in the subepicardial region (subepicardial vessels). In addition, we have analysed the subepicardial area as the combination of the area of subepicardial vessels and the rest of the subepicardial tissue present outside the myocardial layer. This analysis was done at E14.5 comparing *Sox7*^{iECKO} to control embryonic hearts and identified a significant reduction of intramyocardial vessels, subepicardial vessels and subepicardial area. The results suggest that coronary and subepicardial defects are associated to the loss of *Sox7* function in the endothelium (Sup. Fig. 4 A-B). However, all these changes were not maintained at E16.5 (Sup. Fig. 4 C-D),

suggesting that the reduction of these parameters at E14.5 may be due to a delay in epicardium, subepicardium and coronary vessel formation most likely due to the absence of proper growth of the compact layer. Indeed at E16.5, the compact myocardium of *Sox7*^{iECKO} hearts is perfused by coronary vessels and both the subepicardium and its vessels look normal (Sup. Fig. 4C). Interestingly, no major coronary vessels are present inside the compact myocardium of *Sox7*^{iECKO} hearts compared to control (Sup. Fig. 4C, arrow) suggesting that *Sox7* may play a role in the maturation and hierarchization of the coronary vessels. This is consistent with the defects in the connection with the coronary artery stem described in the study in Fig. 3. These results have been integrated in the manuscript in Sup. Fig. 4 and in the results section on pages 10 and 11.

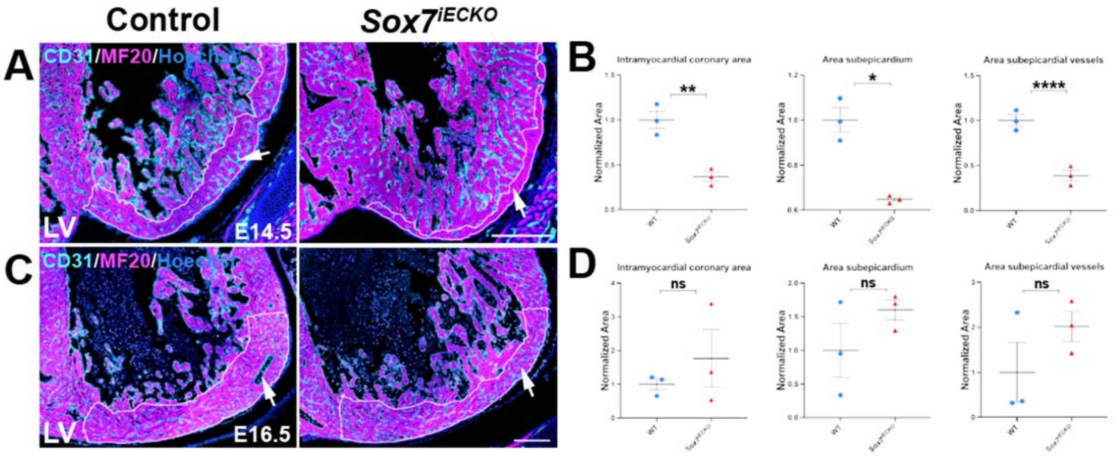

Supplementary Figure 4: Morphometric quantification of epicardial and intramyocardial coronary vessel phenotype. (A, A' and C, C') Immunofluorescence images similar to the ones showed in Fig. 2 highlighting the epicardial and coronary vessel phenotypes comparing Control (left panels) and *Sox7*^{iECKO} (right panels) hearts at E14.5 (A) and E16.5 (C). Myocardium (MF20, magenta), endocardium (CD31, cyan), nuclei (Hoechst, blue). Arrows point at the intra-myocardial coronary vessels Scale bars = 200 µm (A) and 150 µm (C). (B, D) Graphs showing the morphological quantifications of intramyocardial coronary vessel area (left), subepicardial area (middle) and subepicardial vessel area (right) at E14.5 (B) and E16.5 (D). Biological replicates as per the number of data points in the graphs. Mean ± SEM; *t*-test. P <0.05 (*); P<0.005 (**); P<0.0005 (***); P<0.0001 (****), ns = not significant.

4- Fig 3. I suggest to add PECAM staining in the analysis of coronary vessels. It is a more preferable EC marker than VEGFR2

We agree with Reviewer 1 that PECAM (CD31) is broadly accepted as a pan-endothelial cell marker. However, VEGFR2 has been also widely used to stain coronary vessels and to mark sprouting angiogenesis (Su et al., 2018). Here, we are facing a technical limitation, which is the suitability of using CD31 antibody for heart wholemount staining. Despite the fact that CD31 staining is reliable on tissue section, wholemount immunofluorescence requires a TSA amplification step, which is not compatible with late stages of heart development, as the amplification reagents do not penetrate the thick myocardium. The signal amplification only occurs at the periphery whereas the centre of the tissue remains dark. As suggested by

reviewer 1 we attempted to perform wholemount immunofluorescence of PECAM (CD31) in order to visualize coronary vessels and perform 3D volumetric analysis with this marker. As shown in the figure below, PECAM (CD31) staining is suboptimal in wholemount immunofluorescence and does not allow the proper analysis of coronary vessels. As an alternative, we performed PECAM (CD31) analysis of coronary vessels on tissue sections as described in point 3 above.

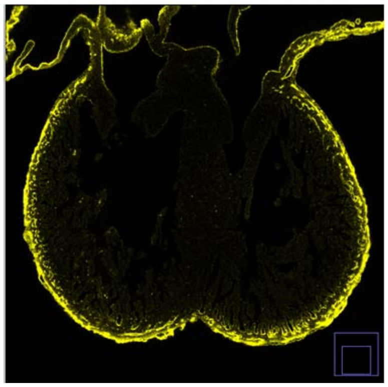

Figure 2 for reviewer only: Wholemount CD31 staining of a E13.5 heart shows a lack of staining in most of the cardiac tissue except at the periphery. This is due to a lack of efficient penetration from the amplification reagents.

5- Which is the effect of Sox7 deletion on subepicardial epicardium?

See response in results presented under point 3.

6- Figure 4 addresses the effect of Sox7 deletion on coronary formation and identify some putative targets by exploiting EC isolated from mice mutants and in vitro in Sox7-silenced HUAEC

The following new experiments are important: 1) the analysis of direct transcriptional activity of SOX7 on CX40 and CX37 ChiP and by luciferase reporter assays in HUEAC; 2) the analysis of Sox7-silenced HUAEC in term of activation (in vitro sprouting activity, proliferation , migration, ephrin B expression); 3) the ability of CX40, CX37, Dll4 to rescue the observed phenotype.

The promoter region of the human CX40 gene has been poorly characterised and was not readily available. To date, only work done in mouse has reported the activity of a Cx40 promoter fragment in non-endothelial cell context (Linhares et al., 2004). We therefore designed a human promoter fragment based on available information on gene regulation from the ENCODE consortium an opted for a regulatory region that ranges from -1190 to +121bp (relative to the TSS). This fragment was cloned into a pGL3basic luciferase reporter plasmid and HUAECs were transfected in presence of the promoter with or without over-expressing a SOX7 expression vector.

A basal activity was detectable, suggesting that this construct contains a minimal promoter that is suitable for the endothelial cell transcriptional machinery. Over-expression of SOX7 in presence of this promoter fragment give rise to an increase of the promoter activity, indicating that CX40 is transcriptionally up-regulated by this transcription factor in arterial cells.

This result brings another line of supporting evidence that Cx40 is a direct target of Sox7 and further support *in vivo* observation that shows a transcriptional regulation of Cx40 in developing zebrafish heart.

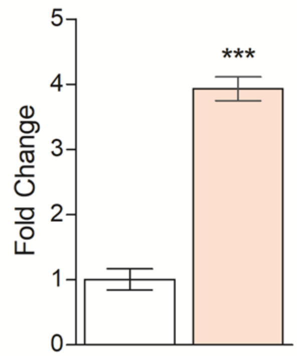

Figure 3 for reviewer only. SOX7 induce CX40 promoter activity in HUAEC cells. A 1Kb fragment of the human CX40 promoter fused to the luciferase gene was transfected with or without SOX7 expression plasmid. We used a dual luciferase reporter system to normalise for transfection efficiency across the conditions, N=3 biological independent repeats, each done with technical triplicate. Open bar: control; salmon bar: SOX7 overexpression.

With respects to a potential rescue experiment we have decided not to engage time and resources to perform this experimental approach based on the following rationale:

1) SOX7 is directly upstream of Cx40/37 (this study) but also of Dll4 (Sacilotto et al., 2013) and Notch1 (Chiang et al., 2017). Impaired *Sox7* function therefore causes perturbations of these two pathways (Cx and Notch). It is most likely that a gain of Cx40 function would be insufficient to rescue the additive phenotypes caused by *Sox7* gene depletion and its associated downstream Notch perturbation.
2) It has been shown that in fact gain of Notch function gives rise to smaller calibre arteries suggesting that non physiological variation of Notch drives to an arrest of coronary artery differentiation (Travisano et al., 2019).
3) Arterial specification progresses with step wise and time sensitive signalling and it is highly unlikely that this molecular approach would have yielded any insights using cells already fully differentiated, which relies on a different gene regulatory network to maintain its identity.

7- The organoid experiment represent an invaluable tool to understand the circuits between EC and cardiomyocytes. The current version of this strategy just directly confirm the in vivo analysis. I suggest to perform a bulk RNAseq on the two separated populations or a secretome analysis by exploiting protein arrays.

We agree that the organoid based model system is a fantastic tool to explore further the cellular cross talk between endothelial cell and cardiomyocytes. The suggested unbiased type of screening experiments here is part of the follow up work to this initial study. There is a great interest in identifying key pathways that modulate the interplay between the two cell types. However, to be informative, these types of proteomic or transcriptomic approaches require an extensive amount of functional validation work, which include gain and loss of function experiments as well as rescue experiments. These are all beyond the scope of this study.

Referee #2:

In this article Chiang et al report that the deletion of Sox7 from endothelial cells results in defective development of coronary blood vessels, cardiomyocytes and hematopoietic cells. They have used many sophisticated approaches including snRNA-seq, bulk RNA-seq, and zebrafish transgenics. The manuscript is also very well written, and I enjoyed reading it. Nevertheless, the main point of the paper i.e., endothelial cells regulate the development of cardiomyocytes is somewhat insufficiently explored.

Major concerns

1. It is pretty clear that Sox7 regulates angiogenesis. Based on previous reports this is not surprising. Using snRNA-seq Chiang et al have identified some intriguing heterogeneity in the ECs that are affected by the loss of Sox7. The C2 cluster appear to be specifically reduced in the mutants. It will be extremely important to determine where these cells are located in vivo. Please select a few specific markers from the C2 cluster and perform IHC or in situ hybridization in control and mutant embryos.

We performed hybridization chain reaction (HCR, single molecule Fluorescent ISH, smFISH) for *Gpc3* and *Sox9* combined with V5 immunofluorescence on embryonic heart sections from E12.5 *Sox7-V5* reporter embryos. We selected both Gpc3 and Sox9 markers because they were found enriched in the C2 endocardial population (snRNA-Seq, Sup. Fig. 7C). Gene co-expression analysis of the C2 cluster shows that around 6% of the endocardial progenitor cells are triple positive for *Sox7*, *Gpc3* and *Sox9*. A staining was performed in the *Sox7-V5* reporter background to assess whether the detection of *Sox9*, *Gpc3*, SOX7 triple-positive cells in the endocardium is viable using a microscopy-based approach.

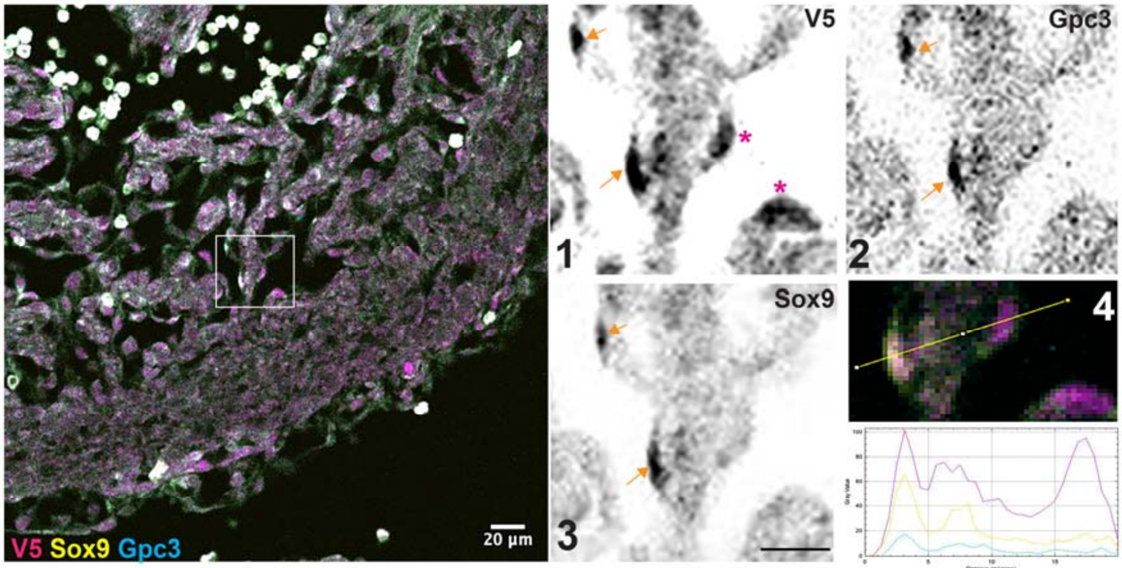

Supplementary Figure 8A. Triple positive Gpc3 Sox9, Sox7 endocardial cells in E12.5 developing hearts. smFISH for *Gpc3* and *Sox9* combined with V5 immunofluorescence in *Sox7-V5* transgenic reporter embryos shows the presence of a subset of trabecular endocardial cells that are triple-positive for SOX7, *Sox9*, *Gpc3* (orange arrows). Pink asterisks, SOX7 single positive cells. Scale bar = 10 um. High magnification from inset with 1,2,3 showing individual channels for each marker; 4 shows the plot profile of fluorophore distribution for triple or single positive cells.

Next we performed individual staining for *Sox9* and *Gpc3* in combination with tissue specific markers (Endomucin, blue, to label the endocardium and SMA, red, to label the myocardium) in order to quantitatively compare control and *Sox7*^{iECKO}. Individual expression analysis of *Gpc3* or *Sox9* in the endocardium showed a scattered distribution for these markers across the atrial and ventricular chambers as well as the atrioventricular canal. This indicates that the C2 population is likely present all throughout the different heart compartments.

When comparing control and *Sox7*^{iECKO}, we observed a general decrease of *Gpc3* and *Sox9-positive* cells in the mutant condition. This is especially evident in the right ventricle endocardium but not as pronounced in the left ventricle (Sup. Fig. 8B). This observation confirms that a subset of the C2 population is lacking expression of genes with stemness potential during *Sox7* loss of function.

These results have been integrated in the new version of the manuscript as Sup. Fig. 8, in the results section on page 15, and the method used in the supplementary methods section on page 40.

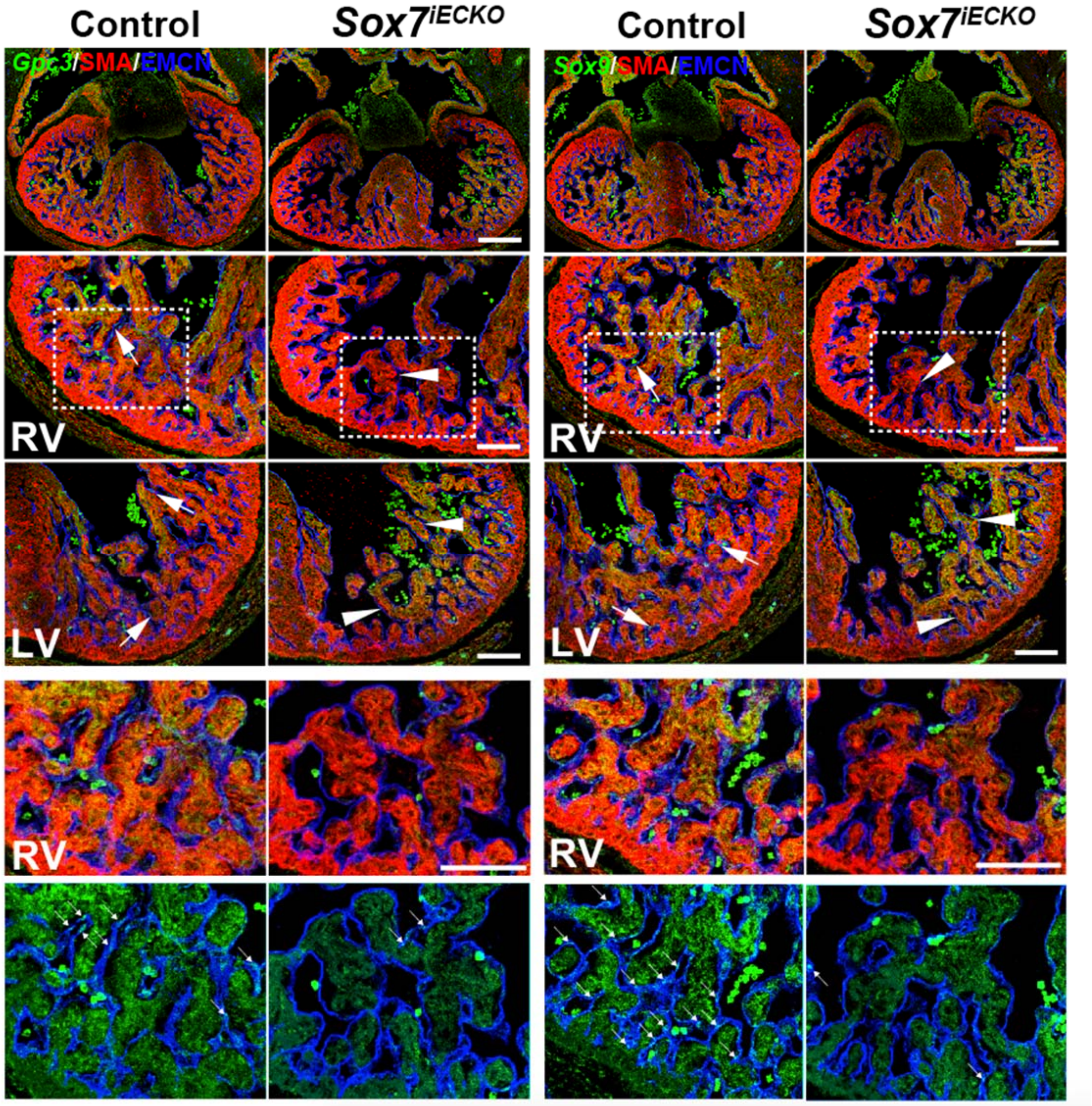

Supplementary Figure 8B. **Expression pattern of C2 population markers.** Analysis by smFISH of the C2 endocardial markers *Gpc3* (left panels), *Sox9* (right panels) in combination with immunofluorescence of endocardial (endomucin, blue) and myocardial (SMA, red) markers on tissues sections of control and *Sox7*^{iECKO} mutant hearts. The 1st row panels are low magnification images showing the entire heart tissue. The 2nd row panels show images of the right ventricles. The 3rd row panels show the left ventricle. The 4th row panels show a high magnification from inset in RV panels and reveal the loss of *Gpc3* and *Sox9* expression in the endocardium of the *Sox7*^{iECKO}. Markers: (green), myocardium (red), endocardium (blue). Scale bars = 200 µm (top panels), 100 µm (middle and bottom panels), arrows indicate *Gpc3* and *Sox9* positive endocardium in the control embryos.

This result is further supported by the analysis of the C2 population snRNA-Seq dataset, which shows that stemness markers (Fig. 4 for reviewer only) are all consistently down-regulated in the *Sox7*^{iECKO} embryo.

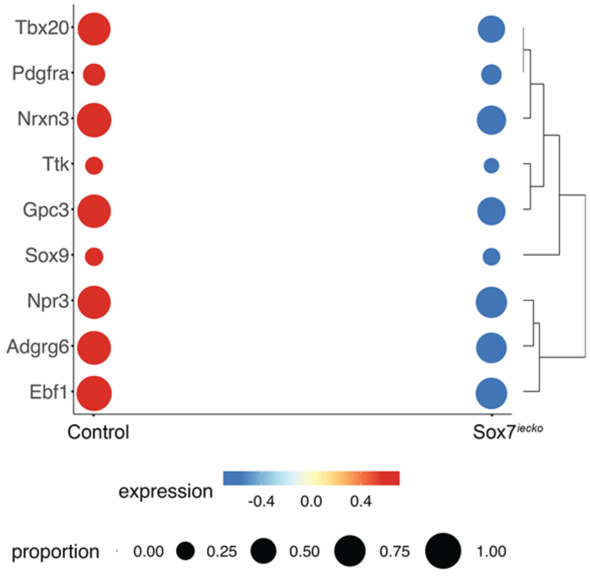

Figure 4 for reviewer only: Stemness markers are globally down regulated in the C2 cluster of the *Sox7*^{iECKO} embryonic heart.

2. The authors demonstrated by bulk RNA-seq that DLL4, Cx40 and Cx37 are reduced in embryos lacking Sox7. Are these genes specifically expressed in the C2 cluster? If not, please explain/discuss the meaning of such a finding.

It is important to note that these three genes (Dll4, Gja4 and Gja5) are not solely restricted to the endothelial cell population found in the C2 cluster and that both C5 and C7 endothelial clusters express these markers too. In addition, cardiomyocytes (C1 and C8) also express Gja5.

The C5 and C7 clusters correspond to differentiated endothelial or endocardial population that could be either coronaries or arterial cells. As shown in Fig. 5D most of C5 and C7 are Npr3-positive (endocardial marker), with only a sub-population of C7 expressing endothelial coronary markers such as Apln and Fabp4. This observation is consistent with a previous study, which showed that Cx37 and Dll4 are expressed at E12.5 in the murine hearts (Su et al., 2018).

Comparison of the expression levels of these three genes across each individual cluster comparing the control and *Sox7*^{iECKO} embryos show that major down regulation for both Gja4 and Dll4 are found in the C5 and C7 clusters of the *Sox7*^{iECKO} embryo (Fig. 5A for reviewer only).

Further, combining all cell populations (cluster C1 to C13) to simulate a bulk RNA-Seq analysis, our data set show a similar trend to the actual bulk RNA-Seq experiment (Sup. Fig. 5A), with a global decrease of Dll4, Gja4 and Gja5 in the *Sox7*^{iECKO} embryonic hearts (Fig. 5B for reviewer only).

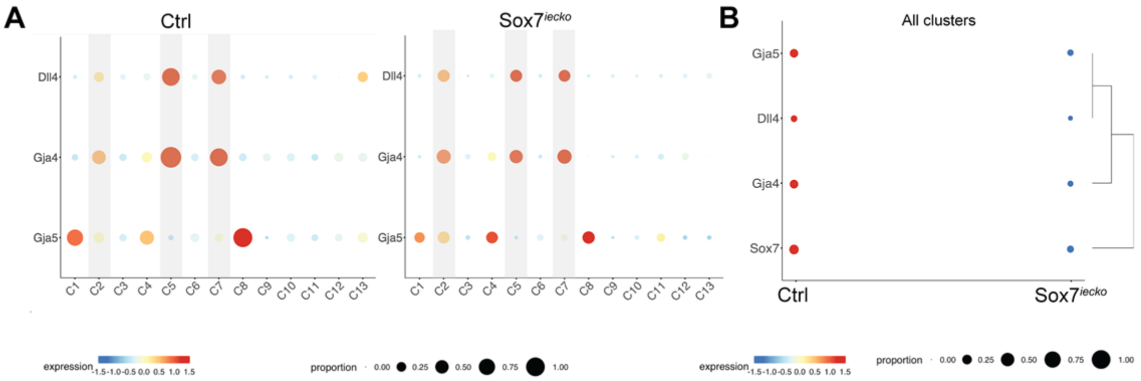

Figure 5 for reviewer only. Analysis of Dll4, Gja4 and Gja5 expression levels at E12.5 from whole heart snRNA-Seq experiment. (A) Change in gene expression levels for each individual clusters. The endocardial cell populations, C2, C5 and C7 clusters, have been highlighted in grey. (B) Change in gene expression levels using all clusters combined (C1 to C13). Blue to red heatmap shows low to high expression levels.

In this study, we are not claiming that the endocardial phenotype (loss of the C2 population) giving rise to a non-compaction phenotype is caused by a change in these three SOX7 direct target genes. It is likely that another molecular circuitry is at play. Possibly, the down-regulation of stemness markers in the C2 population (refer to Fig. 4 for reviewer only) indicates an exhaustion of this cell pool in the *Sox7* mutant heart and therefore causes a cellular imbalance, which contribute to a poor cardiac compaction process.

The change in connexin expression levels and Notch effectors in more mature endothelial cells (C5 and C7 populations) is more likely to contribute to the failure of the major coronary arteries to assemble as proper functional network. Others and we have shown that loss of *Sox7* function in the arterial cell population (Chiang et al., 2017; Sacilotto et al., 2013) drives interference with Notch signalling and perturbs arterio-venous differentiation.

3. The C4 cluster of cardiomyocytes appear to be enriched in mutants. As mentioned above, please select a few specific markers and show that these cells correspond to the trabecular cells as hypothesized.

We performed smFISH for *Hopx* and *Nppa* on E12.5, two of the markers enriched in the C4 myocardial population. In order to distinguish the expression in the different tissues forming the heart, we performed smFISH for these markers in combination with immunofluorescence for endocardial specific markers (endomucin, blue) and myocardium specific markers (SMA, red).

As expected, *Hopx* and *Nppa,* known trabecular myocardium markers, were found restricted to the trabecular myocardium in the ventricular chambers (Sup. Fig. 9). Interestingly, whereas in control embryos, the expression of both markers is restricted towards the trabecular apex (arrows in Sup. Fig. 9A-F, left panels), in *Sox7*^{iECKO} mutant hearts, we observed an ectopic expression that expands to the base of the trabeculae (arrows in Sup. Fig. 9A-F, right panels). This observation is consistent with the expansion of the C4 cluster (differentiation cardiomyocytes) found in the snRNA-Seq analysis (Fig. 5F).

These results have been integrated in the new version of the manuscript in Sup. Fig. 9, in the results section in page 16 and the method used in the supplementary methods section on page 40.

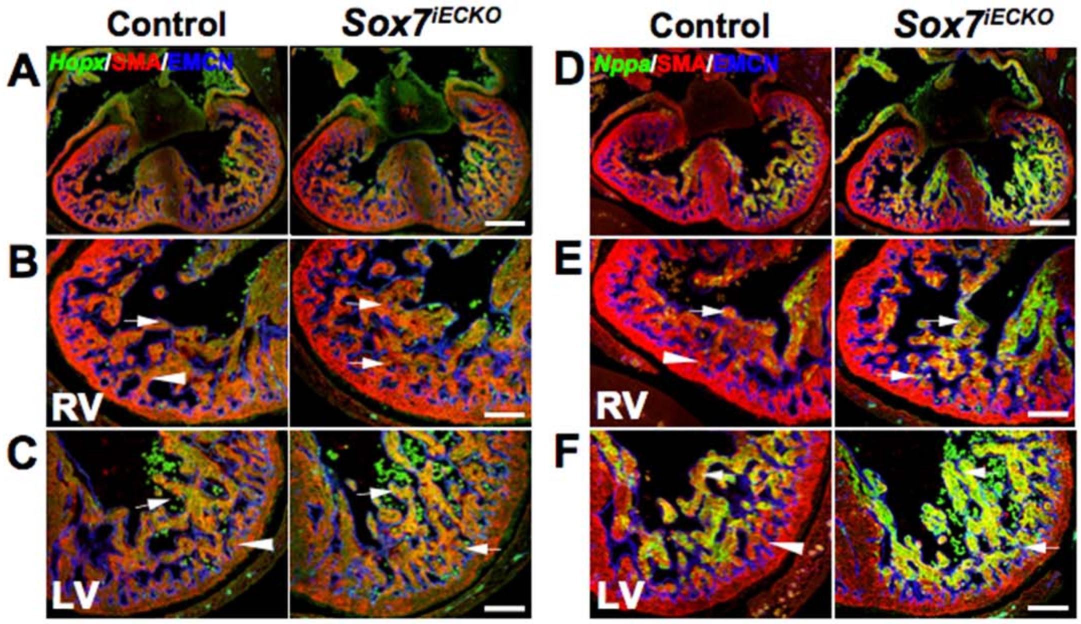

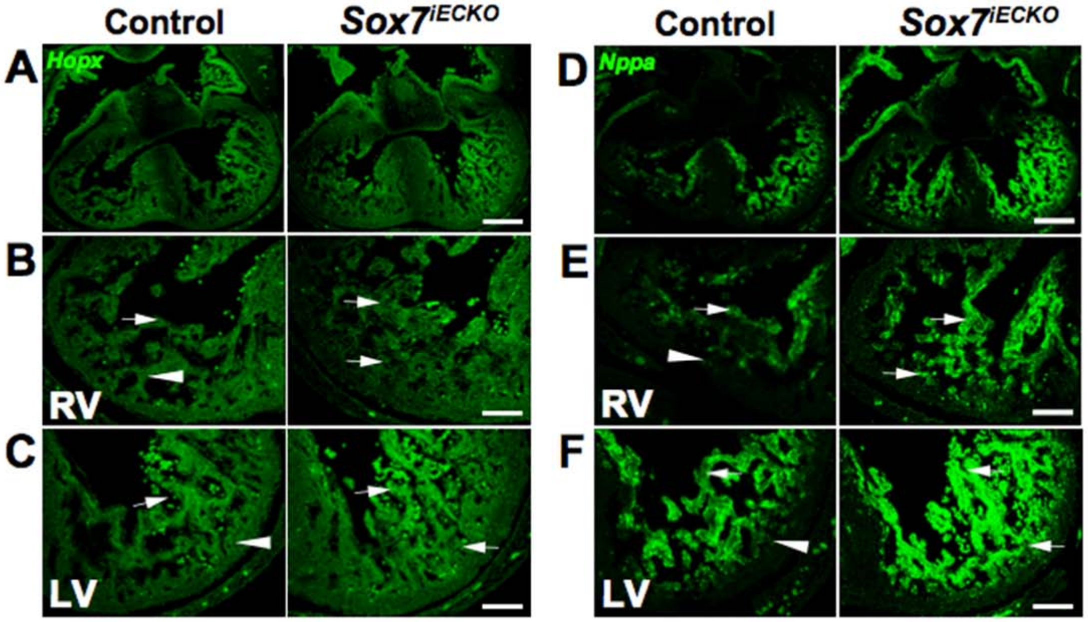

Supplementary Figure 9. **Expression pattern of C4 population markers.** Expression pattern analysis by smFISH of the C4 myocardial markers Hopx (A-C) and Nppa (D-F) in combination with immunofluorescence of endocardial (endomucin) and myocardial (SMA) markers on tissues sections of Control (A-C) and *Sox7*^{iECKO} mutant (D-F) hearts. Low magnification images showing the entire heart tissue (A-F). Marker (green), myocardium (red), endocardium (blue). Scale bars = 100 µm. The green panels show the signal for *Hopx*

and *Nppa* only. Arrows point at the expression of the marker whereas arrowheads point at the lack of expression of the marker in control embryos at the trabecular base.

METHODS for HCR:

Embedding and sectioning.

Embedding and sectioning was performed using standard protocols.

HCR and Immunofluorescence.

Slides were taken from -80^{0}C and incubated for 20 min in the oven at 65^{0}C. Dewaxing was done by placing the slides in Xylene for 3 x 10 min, dehydrating them in ethanol series (100% 2 x 10 min, 90% 2 x 5 min, 70% 5 min, 50% 5 min, 30% 5 min) and finally washing them in 1 x PBS for 2 x 5 minutes. Slides were incubated in 200 µL of probe hybridization buffer and prehybridized in the humidified chamber at 37^{0}C. 2.4pmol of probe solution was added using the following probes: *Gpc3* (NM_016697.3, Molecular Instrument), *Sox9* (NM_011448.4, Molecular Instrument), *Hopx* (NM_175606.3, Molecular Instrument) and *Nppa* (NM_008725.3, Molecular Instrument). After an overnight incubation at 37^{0}C, excess probes were removed by adding 100% probe wash buffer (75% PWB/25% SSCT, 50% PWB/50% SSCT, 25%PWB/75% SSCT and 100% SSCT) for 15 min each. 200 µL of amplification buffer was added and sections pre amplified for 30 min at RT. 12 pmol of hairpin (h1 and h2) were prepared separately by heating at 95^{0}C for 90 sec and then cooling at RT for 30 min. 200 µL of hairpin solution was added on the samples and incubated o/n at RT. Excess hairpins were removed by washing with 5X SSCT for 40 min. Immunofluorescence protocol was followed from this point. Sections were incubated in primary antibody: Endomucin (sc-65495, Santa Cruz Biotechnology) o/n and secondary antibody: Goat anti-rat-A680 (A21096, Thermo Fisher Scientific) for 2 h at RT. The slides were washed with 0.3% Triton-X100 in PBS for 2 x 10 min after each step. Sections were then counterstained with SMA-FITC (F3777-2ML, Sigma-Aldrich) and Hoechst (B2261, Sigma-Aldrich).

Imaging.

Imaging of slides was done using a Leica SP8 inverted microscope. 20x IMM 0.7 N.A. (Glycerol/Oil) objective was selected. The following format was common for all samples: Format: 2048 x 2048, Speed: 600 Hz, Bidirectional: ON and Zoom: 0.75X. To correct for different focus points on different planes of the tissue, a Z-stack was applied with Z-step size=3 using manual Z-axis controller. Due to the big size of the heart section, we used tile scanning. Stitching of the different tiles was automatically done by the LASX software.

Image analysis.

Images in .tif format were opened in Fiji. Channel tool option was selected to assign a colour to each channel. Brightness/Contrast was adjusted and a scale bar was added to the image.

4. I am also curious to know the cluster in which the compact layer cells are located. Based on the results one would predict that these cells are reduced in the mutants.

We have analysed the expression levels of three markers specific to the compact myocardium (Kcnd2, Hey2 and Mycn) in the C1 population. When comparing control and *Sox7*^{iECKO} we observed a decrease of the cells co-expressing these markers consistent with the phenotype that we observe *in vivo* (Fig. 2G, H).
This result has now been included as panel G in Sup. Fig. 9 (shown below) with the compact myocardium population (C1) showing a reduced proportion of triple positive cells for *Mycn*, *Hey2* and *Kcnd2* in the *Sox7*^{iECKO} embryos.

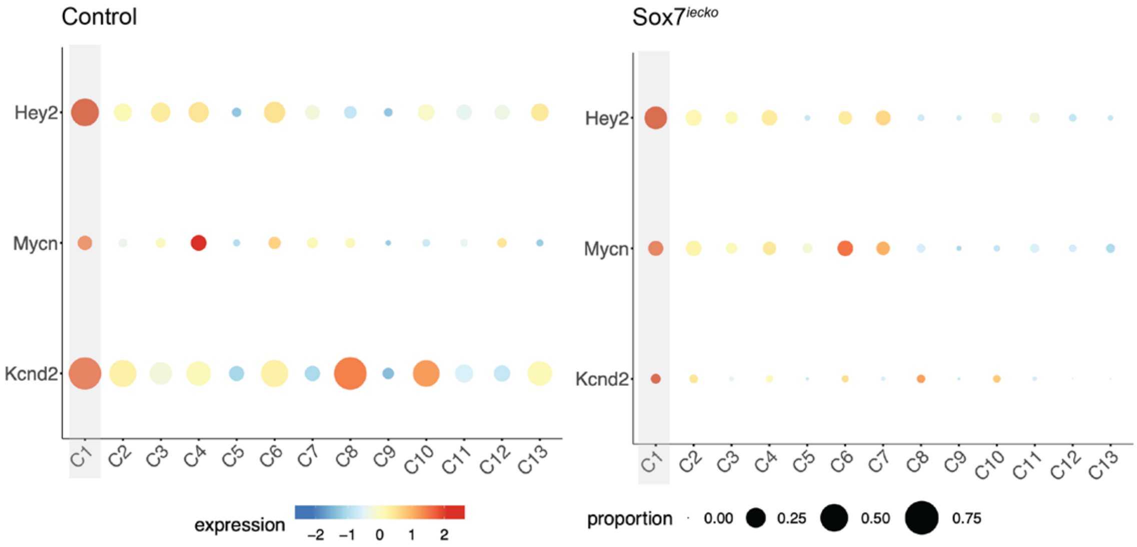

Supplementary Figure 9G. Sn-RNAseq analysis of the compact myocardium cell population (C1, grey highlight) showing the expression levels of known compact layer markers *Mycn*, *Kcnd2* and *Hey2* from snRNA-Seq analysis. Comparison between control and *Sox7*^{iECKO} shows a decrease in the number of cells expressing these 3 markers in mutant embryos.

5. The changes in the trabecular and compact layers are very hard to appreciate. They appear comparable to me. The authors should try to explain this analysis better, may be with a schematic, and demonstrate convincingly that there are indeed some changes.

We thank the reviewer for this suggestion to improve the clarity of the phenotypic characterisation. In order to make the changes in the trabecular and compact myocardium more obvious, we have now included a white contour depicting the compact layer equivalent to the compact myocardial mask used for tissue area quantifications. We have included these contours in the RV and LV images in control and *Sox7*^{iECKO} hearts at E14.5 in Fig. 2, and at E11.5, E12.5, E13.5 and E16.5 in Sup. Fig. 1.

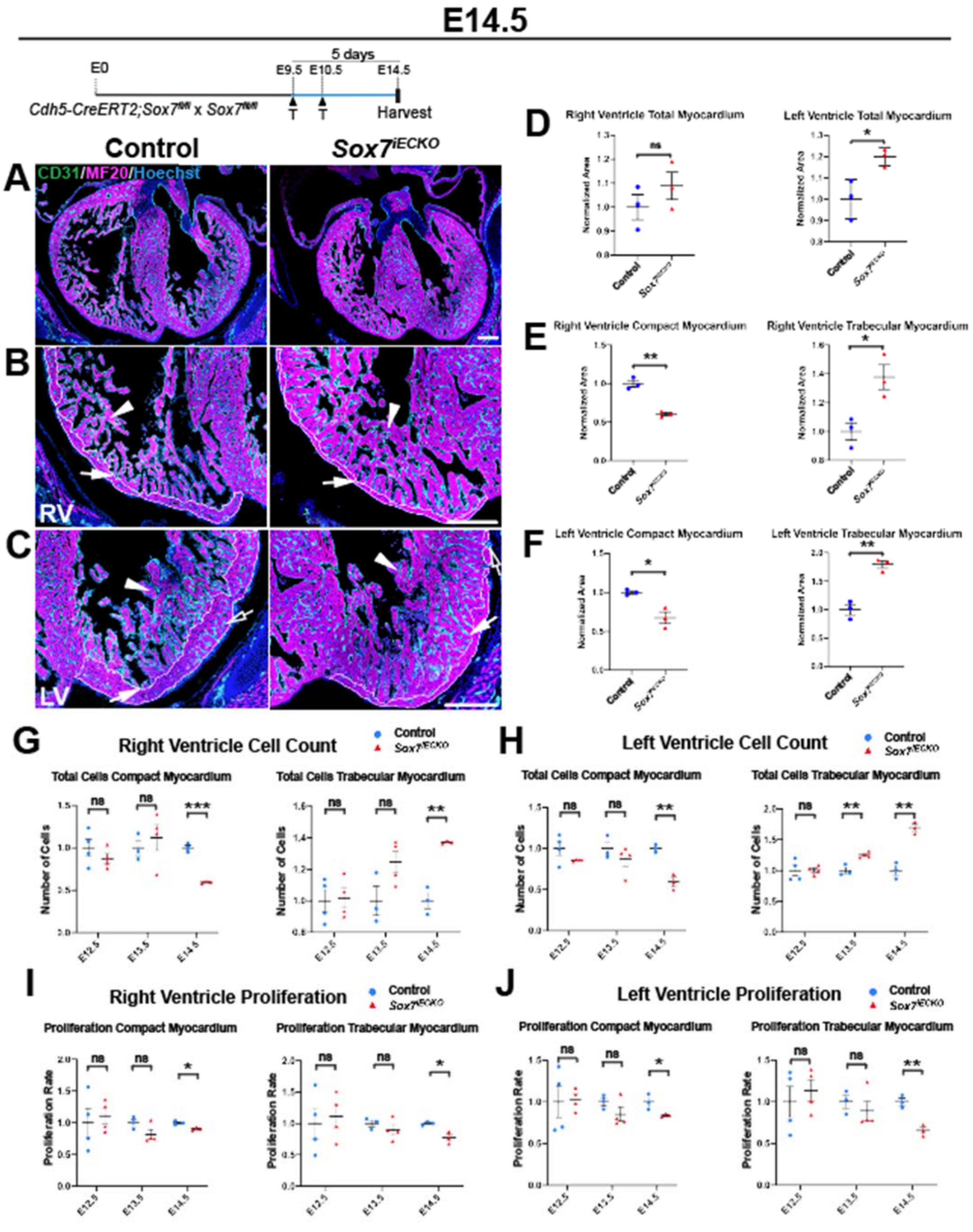
E14.5
5 days
E0
E9.5
E10.5
E14.5
Cdh5-CreERT2;Sox7fl/fl x Sox7fl/fl
Harvest
Control
Sox7iECKO
A
CD31/MF20/Hoechst
B
RV
C
LV
D
Right Ventricle Total Myocardium
Left Ventricle Total Myocardium
E
Right Ventricle Compact Myocardium
Right Ventricle Trabecular Myocardium
F
Left Ventricle Compact Myocardium
Left Ventricle Trabecular Myocardium
Normalized Area
G
Right Ventricle Cell Count
H
Left Ventricle Cell Count
Total Cells Compact Myocardium
Total Cells Trabecular Myocardium
Number of Cells
I
Right Ventricle Proliferation
J
Left Ventricle Proliferation
Proliferation Compact Myocardium
Proliferation Trabecular Myocardium
Proliferation Rate
Control
Sox7iECKO
E12.5
E13.5
E14.5

Fig. 2

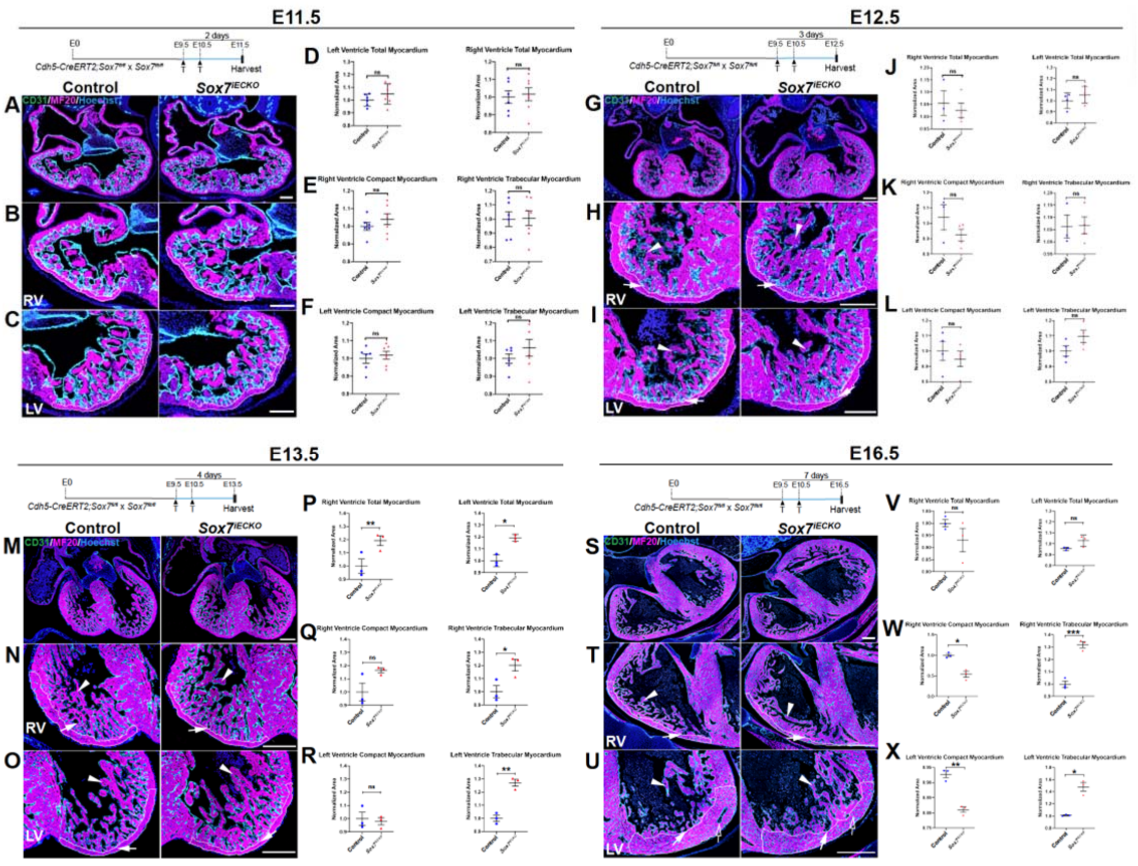

Sup. Fig 1

The cardiac phenotype described in these figures identified a thinning of the compact myocardium and an expansion of the trabecular myocardium in *Sox7*^{iECKO} compared to control hearts. This trabecular expansion is further validated by 1) snRNA-Seq analysis showing the expansion of the cardiomyocyte cluster C4 (trabecular myocardium, Fig. 5F) and 2) the validations by smFISH of C4 markers *Nppa* and *Hopx* (known trabecular markers) shown Sup. Fig. 9A-F (see also response in query #3).

6. The zebrafish Cx40 transgenics is elegant. However, it will be more helpful to show that the expression of this reporter is reduced or absent in zebrafish embryos treated with Sox7 MO. If not, please explain the result.

We thank the reviewer for the suggestion to further confirm the transcriptional relationship between Sox7 and Cx40. However, we believe that this molecular link has been firmly established in the current study, over multiple assays both *in vivo* using bulk RNA-Seq, single nucleus RNA-Seq, *in situ* hybridisation and immunofluorescence, and *in vitro* with endothelial cells using siRNA-based gene depletion. All these experiments confirmed that Cx40 expression is downregulated with the loss of SOX7 function. Therefore, based on the current evidences provided by an orthogonal validation approach, we have decided not to perform the analysis in the zebrafish model system.

7. Does lineage tracing support the argument that Sox7-/- endocardial cells transdifferentiate into hematopoietic cells? If not, please explain the result.

We have interrogated the snRNA-Seq data set to assess the expression pattern of hemogenic markers in the C2 population. We observed that key markers for the hemangiogenic endothelium are up-regulated in the *Sox7*^{iECKO} samples.

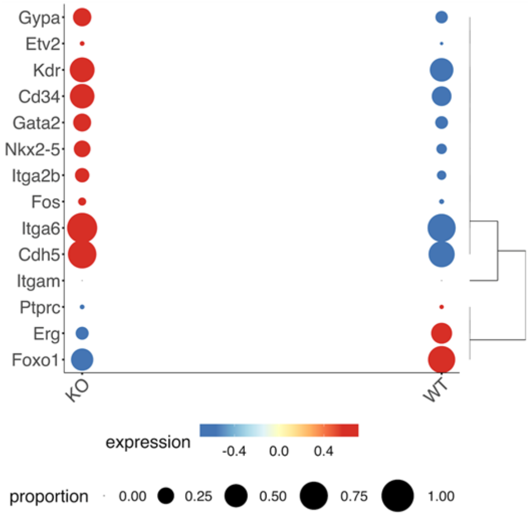

Figure 5 for reviewer only. Comparison of hemogenic markers in the C2 progenitor population between control and *Sox7*^{iECKO} E12.5 shows an overall increase in the mutant condition.

Next, we further interrogated the expression of erythro-myeloid markers *in vivo* in the *Sox7*^{iECKO}; *mT/mG* genetic background to assess whether Sox7-null endothelial have the potential to transdifferentiate in this cell lineage. Analysis of E13.5 embryonic heart from *Sox7*^{iECKO} *;* mT/mG reporter line showed that GFP positive endothelial cells (eg. Sox7-null) harbour the expression of hematopoietic stem cell markers CD34 and pan hematopoietic marker CD45.

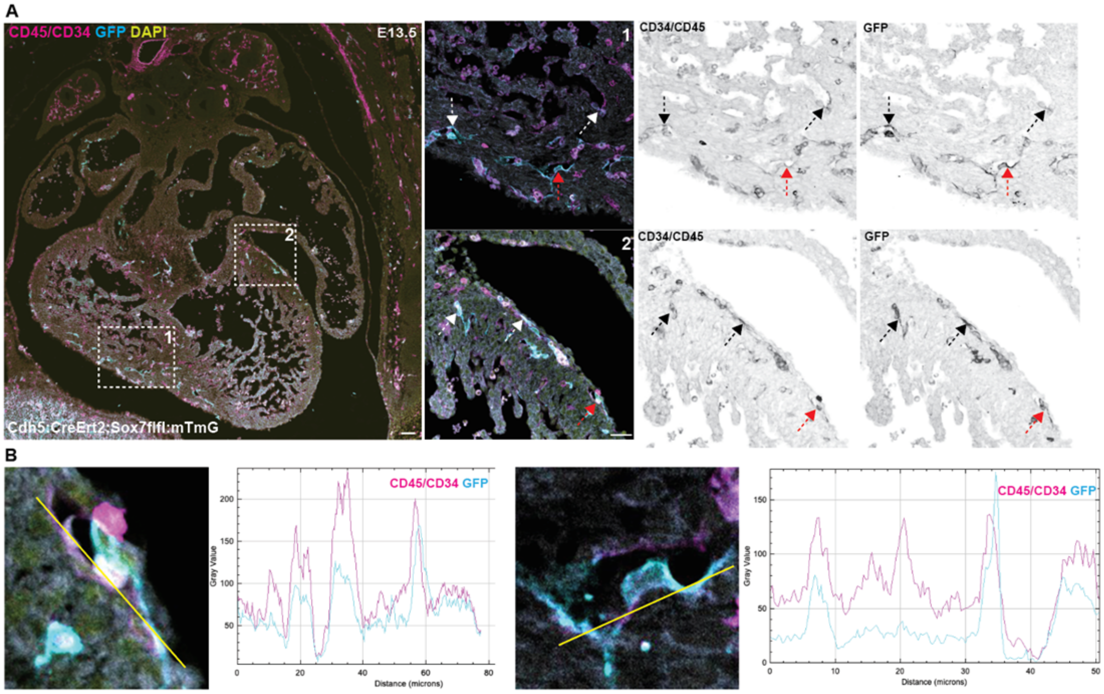

Supplementary Figure 13: Sox7-null endothelial cells transdifferentiate into hematopoietic cell lineages *in vivo*. (**A**) Transverse section of an E13.5 heart from a triple transgenic *Sox7*^{iECKO}*; mT/mG* embryo stained for GFP, CD45/CD34 (same fluorophore) and DAPI. This staining shows the presence of GFP positive endothelial cells in the coronary vasculature that express either CD34 or CD45 hematopoietic markers (red and black arrows). (**B**) Plot profile analysis of a cell from the inner lining of a vessel showing the colocalization of the fluorescent signal. These regions are blown up areas indicated by the red arrows. Scale bar = 20um (panels for inset 1 and 2).

Of note, our fluorophore option to label CD34 and CD45 positive cells was limited by the mT/mG background, which uses most of the visible spectrum (488 and 555nm). tdTomato is constitutively expressed and shifted to GFP upon Cre activation. Due to this technical constraint we had to combine both CD34 and CD45 under the same fluorophore (eg. 647nm) to ensure a complete lack of bleed through from the GFP channel. As a consequence, we cannot discriminate the exact cell distribution of these two hematopoietic markers. Interestingly, the C13 cluster that corresponds to the macrophage population enriched in the *Sox7*^{iECKO} (Fig. 5F), also co-expresses CD34 and CD45.

These results have been integrated in the new version of the manuscript in Sup. Fig. 13, and in the results section in page 18.

1. Page 23: "non-statistically significant" should probably be "statistically non-significant".

We have made the edit following the reviewer's suggestion**.**

2. Page 32: What are monocle algorithm and pseudo time analysis? Please explain these approaches in a manner that common readers can understand.

Monocle algorithm is designed to determine cell fate trajectories and several possible end states along this trajectory to infer transcription dynamics.

Pseudotime is a concept used in the Monocle algorithm that refers to a continuous measure of the developmental or differentiation state of individual cells, inferred from their gene expression profiles. Pseudotime ordering is based on the assumption that cells in a given biological process follow a trajectory, and that genes are expressed in a coordinated manner along this trajectory. By assigning each cell a pseudotime value, the Monocle algorithm can order the cells along a trajectory and identify genes whose expression changes over time.

References

Chiang, I.K.-N., Fritzsche, M., Pichol-Thievend, C., Neal, A., Holmes, K., Lagendijk, A., Overman, J., Angelo, D., Omini, A., Hermkens, D., Lesieur, E., Liu, K., Ratnayaka, I., Corada, M., Bou-Gharios, G., Carroll, J., Dejana, E., Schulte-Merker, S., Hogan, B., Beltrame, M., De Val, S., Francois, M., 2017. SoxF factors induce Notch1 expression via direct transcriptional regulation during early arterial development. Development 144, 2629-2639.

Chiang, I.K.N., Graus, M.S., Kirschnick, N., Davidson, T., Luu, W., Harwood, R., Jiang, K., Li, B., Wong, Y.Y., Moustaqil, M., Lesieur, E., Skoczylas, R., Kouskoff, V., Kazenwadel, J., Arriola-Martinez, L., Sierecki, E., Gambin, Y., Alitalo, K., Kiefer, F., Harvey, N.L., Francois, M., 2023. The blood vasculature instructs lymphatic patterning in a SOX7-dependent manner. The EMBO journal 42, e109032.

Del Monte-Nieto, G., Ramialison, M., Adam, A.A.S., Wu, B., Aharonov, A., D'Uva, G., Bourke, L.M., Pitulescu, M.E., Chen, H., de la Pompa, J.L., Shou, W., Adams, R.H., Harten, S.K., Tzahor, E., Zhou, B., Harvey, R.P., 2018. Control of cardiac jelly dynamics by NOTCH1 and NRG1 defines the building plan for trabeculation. Nature 557, 439-445.

Linhares, V.L.F., Almeida, N.A.S., Menezes, D.C., Elliott, D.A., Lai, D., Beyer, E.C., Campos de Carvalho, A.C., Costa, M.W., 2004. Transcriptional regulation of the murine Connexin40 promoter by cardiac factors Nkx2-5, GATA4 and Tbx5. Cardiovascular research 64, 402-411.

Sacilotto, N., Monteiro, R., Fritzsche, M., Becker, P.W., Sanchez-del-Campo, L., Liu, K., Pinheiro, P., Ratnayaka, I., Davies, B., Goding, C.R., Patient, R., Bou-Gharios, G., De Val, S., 2013. Analysis of Dll4 regulation reveals a combinatorial role for Sox and Notch in arterial development. Proceedings of the National Academy of Sciences 110, 11893-11898.

Schmidt, U., Weigert, M., Broaddus, C., Myers, G., 2018. Cell Detection with Star-Convex Polygons, in: Frangi, A.F., Schnabel, J.A., Davatzikos, C., Alberola-López, C., Fichtinger, G. (Eds.), Medical Image Computing and Computer Assisted Intervention – MICCAI 2018. Springer International Publishing, Cham, pp. 265-273.

Su, T., Stanley, G., Sinha, R., D'Amato, G., Das, S., Rhee, S., Chang, A.H., Poduri, A., Raftrey, B., Dinh, T.T., Roper, W.A., Li, G., Quinn, K.E., Caron, K.M., Wu, S., Miquerol, L., Butcher, E.C., Weissman, I., Quake, S., Red-Horse, K., 2018. Single-cell analysis of early progenitor cells that build coronary arteries. Nature 559, 356-362.

Travisano, S.I., Oliveira, V.L., Prados, B., Grego-Bessa, J., Piñeiro-Sabarís, R., Bou, V., Gómez, M.J., Sánchez-Cabo, F., MacGrogan, D., de la Pompa, J.L., 2019. Coronary arterial development is regulated by a Dll4-Jag1-EphrinB2 signaling cascade. eLife 8, e49977.

Dear Mat,

Thank you for the submission of your revised manuscript to EMBO reports. We have now received the full set of referee reports that is copied below.

As you will see, all referees are very positive about the study and recommend publication.
Before I can accept the manuscript, I need you to address some minor points below:

- Please reduce the number of keywords to 5.

- Please update the 'Conflict of interest' paragraph to our new 'Disclosure and competing interests statement'. For more information see
https://www.embopress.org/page/journal/14693178/authorguide#conflictsofinterest

- Please remove the Author Contributions from the manuscript and make sure that the author contributions in our online submission system are correct and up-to-date. The information you specified in the system will be automatically retrieved and typeset into the article. You can enter additional information in the free text box provided, if you wish.

- Please update the references to the alphabetical Harvard style. The abbreviation 'et al' should be used if more than 10 authors. You can download the respective EndNote file from our Guide to Authors https://endnote.com/style_download/embo-reports/

- You refer to 'data not shown' on pages 23 & 27. Please note our editorial policy that all data cited needs to be shown in the manuscript.

- Please complete the column "Information included in the manuscript?' in the Author Checklist by choosing either YES or Not applicable. Moreover, you indicated that you have checked cell lines for mycoplasma contamination, but I could not find this information in the Methods section.

- Please state the ethics approval reference number IK 084/19 also in the methods section.

- Please incorporate the Supplementary Methods to the main Materials and Methods section as we require that all information is in the main manuscript file.

- Data availability section: Please add a link that resolves to the dataset on GEO.

- Grant # APP1111169 needs correcting in the ms, it has one"P" missing.

- Please order the manuscript sections as outlined in our Guide to Authors.

- Please combine all 13 Supplementary figures into one PDF file that is called Appendix. The figure legends need to be part of the Appendix file and you need a table of content with page numbers. The nomenclature is 'Appendix Figure S#".

- End of May we had communicated by e-mail about the deposition of Source Data to the Bioimage repository. If this has succeeded, please add a link to the source data in the Data availability section.

- We also talked about the apparent redundancy of figure panels and you clarified that this is due to the image quantification method used. You proposed to add a simple schematic of the workflow to the method section to make this clear, which I think is a good idea.

- Please add callouts to the following figure panels in the text (currently they are not called out): Fig 6 F-I; Supp. Fig. 10A&B; Supp. Fig. 12A&B; Supp. Fig. 13A&B.
- Supp. Fig 6A is called out before Supp. Fig 5, maybe you could reorder those two?

- The panels showing quantifications in Figure S1 are still rather small. Maybe splitting this figure into two might help?

- I attach to this email a related manuscript file with comments by our data editors. Please address all comments and upload a revised file with tracked changes with your final manuscript submission. Note that the Data availability section you added is missing from this version.

- Finally, EMBO reports papers are accompanied online by A) a short (1-2 sentences) summary of the findings and their significance, B) 2-3 bullet points highlighting key results and C) a synopsis image that is 550x300-600 pixels large (width x height) in PNG for JPG format. You can either show a model or key data in the synopsis image. Please note that the size is

rather small and that text needs to be readable at the final size. Please send us this information along with the revised manuscript.

We look forward to seeing a final version of your manuscript as soon as possible.

With kind regards,

Martina

Martina Rembold, PhD
Senior Editor
EMBO reports

****

Referee #1:

The authors exhaustively replied to my criticisms

Referee #2:

This work is novel and employs several elegant tools. The revised manuscript is significantly improved. The authors have gone above and beyond in responding to my comments and suggestions. I have no further questions or concerns.

To Dr. Rembold
EMBO Reports

13th July 2023

Dear Dr. Rembold,

There is no need for a point by point response to the reviewers.
Both reviewers are extremely positive with the current version of the manuscript and recommend this work for publication.

Sincerely
mat

Prof. Mathias Francois
University of Sydney
Centenary Institute
Bldg D17, CPC
Camperdown
Sydney, NSW 2006
Australia

Dear Mat,

I am very pleased to accept your manuscript for publication in the next available issue of EMBO reports. Thank you for your contribution to our journal.

At the end of this email I include important information about how to proceed. Please ensure that you take the time to read the information and complete and return the necessary forms to allow us to publish your manuscript as quickly as possible.

As part of the EMBO publication's Transparent Editorial Process, EMBO reports publishes online a Review Process File to accompany accepted manuscripts. As you are aware, this File will be published in conjunction with your paper and will include the referee reports, your point-by-point response and all pertinent correspondence relating to the manuscript.

If you do NOT want this File to be published, please inform the editorial office within 2 days, if you have not done so already, otherwise the File will be published by default [contact: emboreports@embo.org]. If you do opt out, the Review Process File link will point to the following statement: "No Review Process File is available with this article, as the authors have chosen not to make the review process public in this case."

Thank you again for your contribution to EMBO reports and congratulations on a successful publication. Please consider us again in the future for your most exciting work.

Kind regards,

Martina

Martina Rembold, PhD
Senior Editor
EMBO reports

****

THINGS TO DO NOW:

Please note that you will be contacted by Wiley Author Services to complete licensing and payment information. The required 'Page Charges Authorization Form' is available here: https://www.embopress.org/pb-assets/embo-site/er_apc.pdf - please download and complete the form and return to embopressproduction@wiley.com

EMBO Press participates in many Publish and Read agreements that allow authors to publish Open Access with reduced/no publication charges. Check your eligibility: https://authorservices.wiley.com/author-resources/Journal-Authors/open-access/affiliation-policies-payments/index.html

You will receive proofs by e-mail approximately 2-3 weeks after all relevant files have been sent to our Production Office; you should return your corrections within 2 days of receiving the proofs.

Please inform us if there is likely to be any difficulty in reaching you at the above address at that time. Failure to meet our deadlines may result in a delay of publication, or publication without your corrections.

All further communications concerning your paper should quote reference number EMBOR-2022-55043V4 and be addressed to emboreports@wiley.com.

Should you be planning a Press Release on your article, please get in contact with emboreports@wiley.com as early as possible, in order to coordinate publication and release dates.

EMBO Press Author Checklist

Corresponding Author Name: Mathias Francois	
Journal Submitted to: EMBO Reports	
Manuscript Number: EMBOR-2022-55043V2	

USEFUL LINKS FOR COMPLETING THIS FORM
The EMBO Journal - Author Guidelines
EMBO Reports - Author Guidelines
Molecular Systems Biology - Author Guidelines
EMBO Molecular Medicine - Author Guidelines

Reporting Checklist for Life Science Articles (updated January 2022)
This checklist is adapted from Materials Design Analysis Reporting (MDAR) Checklist for Authors. MDAR establishes a minimum set of requirements in transparent reporting in the life sciences (see Statement of Task: 10.31222/osf.io/9sm4x). Please follow the journal's guidelines in preparing your manuscript.
Please note that a copy of this checklist will be published alongside your article.

Abridged guidelines for figures
1. Data
The data shown in figures should satisfy the following conditions:
- the data were obtained and processed according to the field's best practice and are presented to reflect the results of the experiments in an accurate and unbiased manner.
- ideally, figure panels should include only measurements that are directly comparable to each other and obtained with the same assay.
- plots include clearly labeled error bars for independent experiments and sample sizes. Unless justified, error bars should not be shown for technical replicates.
- if n<5, the individual data points from each experiment should be plotted. Any statistical test employed should be justified.
- Source Data should be included to report the data underlying figures according to the guidelines set out in the authorship guidelines on Data Presentation.

2. Captions
Each figure caption should contain the following information, for each panel where they are relevant:
- a specification of the experimental system investigated (eg cell line, species name).
- the assay(s) and method(s) used to carry out the reported observations and measurements.
- an explicit mention of the biological and chemical entity(ies) that are being measured.
- an explicit mention of the biological and chemical entity(ies) that are altered/varied/perturbed in a controlled manner.
- the exact sample size (n) for each experimental group/condition, given as a number, not a range;
- a description of the sample collection allowing the reader to understand whether the samples represent technical or biological replicates (including how many animals, litters, cultures, etc.).
- a statement of how many times the experiment shown was independently replicated in the laboratory.
- definitions of statistical methods and measures:
  - common tests, such as t-test (please specify whether paired vs. unpaired), simple χ2 tests, Wilcoxon and Mann-Whitney tests, can be unambiguously identified by name only, but more complex techniques should be described in the methods section;
  - are tests one-sided or two-sided?
  - are there adjustments for multiple comparisons?
  - exact statistical test results, e.g., P values = x but not P values < x;
  - definition of 'center values' as median or average;
  - definition of error bars as s.d. or s.e.m.

Please complete ALL of the questions below.
Select "Not Applicable" only when the requested information is not relevant for your study.

Materials

Newly Created Materials	Information included in the manuscript?	In which section is the information available? (Reagents and Tools Table, Materials and Methods, Figures, Data Availability Section)
New materials and reagents need to be available; do any restrictions apply?	Not Applicable	NO

Antibodies	Information included in the manuscript?	In which section is the information available? (Reagents and Tools Table, Materials and Methods, Figures, Data Availability Section)
For **antibodies** provide the following information:<br>- Commercial antibodies: RRID (if possible) or supplier name, catalogue number and or/clone number<br>- Non-commercial: RRID or citation	Yes	Material and methods section

DNA and RNA sequences	Information included in the manuscript?	In which section is the information available? (Reagents and Tools Table, Materials and Methods, Figures, Data Availability Section)
**Short novel DNA or RNA including primers, probes**: provide the sequences.	Yes	Material and methods section

Cell materials	Information included in the manuscript?	In which section is the information available? (Reagents and Tools Table, Materials and Methods, Figures, Data Availability Section)
**Cell lines:** Provide species information, strain. Provide accession number in repository **OR** supplier name, catalog number, clone number, and/**OR** RRID.	Yes	Material and methods section
**Primary cultures:** Provide species, strain, sex of origin, genetic modification status.	Yes	Material and methods section
Report if the cell lines were recently **authenticated** (e.g., by STR profiling) and tested for mycoplasma contamination.	Yes	Material and methods section

Experimental animals	Information included in the manuscript?	In which section is the information available? (Reagents and Tools Table, Materials and Methods, Figures, Data Availability Section)
**Laboratory animals or Model organisms:** Provide species, strain, sex, age, genetic modification status. Provide accession number in repository **OR** supplier name, catalog number, clone number, **OR** RRID.	Yes	Material and methods section
**Animal observed in or captured from the field:** Provide species, sex, and age where possible.	Not Applicable	NA
Please detail **housing and husbandry conditions**.	Yes	Material and methods section

Plants and microbes	Information included in the manuscript?	In which section is the information available? (Reagents and Tools Table, Materials and Methods, Figures, Data Availability Section)
**Plants:** provide species and strain, ecotype and cultivar where relevant, unique accession number if available, and source (including location for collected wild specimens).	Not Applicable	NA
**Microbes:** provide species and strain, unique accession number if available, and source.	Not Applicable	NA

Human research participants	Information included in the manuscript?	In which section is the information available? (Reagents and Tools Table, Materials and Methods, Figures, Data Availability Section)
If collected and within the bounds of privacy constraints report on age, sex and gender or ethnicity for all study participants.	Not Applicable	NA

Core facilities	Information included in the manuscript?	In which section is the information available? (Reagents and Tools Table, Materials and Methods, Figures, Data Availability Section)
If your work benefited from core facilities, was their service mentioned in the acknowledgments section?	Yes	yes

Design

Study protocol	Information included in the manuscript?	In which section is the information available? (Reagents and Tools Table, Materials and Methods, Figures, Data Availability Section)
If study protocol has been **pre-registered, provide DOI in the manuscript**. For clinical trials, provide the trial registration number **OR** cite DOI.	Not Applicable	
Report the **clinical trial registration number** (at ClinicalTrials.gov or equivalent), where applicable.	Not Applicable	

Laboratory protocol	Information included in the manuscript?	In which section is the information available? (Reagents and Tools Table, Materials and Methods, Figures, Data Availability Section)
Provide DOI OR other citation details if **external detailed step-by-step protocols** are available.	Yes	Material and methods section

Experimental study design and statistics	Information included in the manuscript?	In which section is the information available? (Reagents and Tools Table, Materials and Methods, Figures, Data Availability Section)
Include a statement about **sample size** estimate even if no statistical methods were used.	Yes	Material and methods section
Were any steps taken to minimize the effects of subjective bias when allocating animals/samples to treatment (e.g. **randomization procedure**)? If yes, have they been described?	Yes	Material and method section
Include a statement about **blinding** even if no blinding was done.	Not Applicable	
Describe **inclusion/exclusion criteria** if samples or animals were excluded from the analysis. Were the criteria pre-established?<br><br>If sample or data points were omitted from analysis, report if this was due to attrition or intentional exclusion and provide justification.	Not Applicable	
For every figure, are **statistical tests** justified as appropriate? Do the data meet the assumptions of the tests (e.g., normal distribution)? Describe any methods used to assess it. Is there an estimate of variation within each group of data? Is the variance similar between the groups that are being statistically compared?	Yes	Material and methods section and figure legends

Sample definition and in-laboratory replication	Information included in the manuscript?	In which section is the information available? (Reagents and Tools Table, Materials and Methods, Figures, Data Availability Section)
In the figure legends: state number of times the experiment was **replicated** in laboratory.	Yes	figure legends
In the figure legends: define whether data describe **technical or biological replicates**.	Yes	figure legends

Ethics

Ethics	Information included in the manuscript?	In which section is the information available? (Reagents and Tools Table, Materials and Methods, Figures, Data Availability Section)
Studies involving **human participants**: State details of **authority granting ethics approval** (IRB or equivalent committee(s), provide reference number for approval.	Not Applicable	
Studies involving **human participants**: Include a statement confirming that **informed consent** was obtained from all subjects and that the experiments conformed to the principles set out in the WMA Declaration of Helsinki and the Department of Health and Human Services Belmont Report.	Not Applicable	
Studies involving **human participants:** For publication of **patient photos**, include a statement confirming that consent to publish was obtained.	Not Applicable	
Studies involving experimental **animals**: State details of **authority granting ethics approval** (IRB or equivalent committee(s), provide reference number for approval. Include a statement of compliance with ethical regulations.	Yes	All animal work was approved by the relevant ethics committees of the University of Queensland and University of Sydney. ID: 084/19 Experimental Analysis of Lymphangeogenesis in development and regeneration
Studies involving **specimen and field samples:** State if relevant **permits** obtained, provide details of authority approving study; if none were required, explain why.	Not Applicable	

Dual Use Research of Concern (DURC)	Information included in the manuscript?	In which section is the information available? (Reagents and Tools Table, Materials and Methods, Figures, Data Availability Section)
Could your study fall under dual use research restrictions? Please check biosecurity documents and list of **select agents and toxins** (CDC): https://www.selectagents.gov/sat/list.htm	Not Applicable	
If you used a select agent, is the security level of the lab appropriate and reported in the manuscript?	Not Applicable	
If a study is subject to dual use research of concern regulations, is the name of the **authority granting approval and reference number** for the regulatory approval provided in the manuscript?	Not Applicable	

Reporting

The MDAR framework recommends adoption of discipline-specific guidelines, established and endorsed through community initiatives. Journals have their own policy about requiring specific guidelines and recommendations to complement MDAR.

Adherence to community standards	Information included in the manuscript?	In which section is the information available? (Reagents and Tools Table, Materials and Methods, Figures, Data Availability Section)
State if relevant guidelines or checklists (e.g., **ICMJE, MIBBI, ARRIVE, PRISMA**) have been followed or provided.	Not Applicable	
For **tumor marker prognostic studies,** we recommend that you follow the **REMARK** reporting guidelines (see link list at top right). See author guidelines, under 'Reporting Guidelines'. Please confirm you have followed these guidelines.	Not Applicable	
For **phase II and III randomized controlled trials**, please refer to the **CONSORT** flow diagram (see link list at top right) and submit the CONSORT checklist (see link list at top right) with your submission. See author guidelines, under 'Reporting Guidelines'. Please confirm you have submitted this list.	Not Applicable	

Data Availability

Data availability	Information included in the manuscript?	In which section is the information available? (Reagents and Tools Table, Materials and Methods, Figures, Data Availability Section)
Have **primary datasets** been deposited according to the journal's guidelines (see 'Data Deposition' section) and the respective accession numbers provided in the Data Availability Section?	Yes	Material and methods section
Were **human clinical and genomic datasets** deposited in a public access-controlled repository in accordance to ethical obligations to the patients and to the applicable consent agreement?	Not Applicable	
Are **computational models** that are central and integral to a study available without restrictions in a machine-readable form? Were the relevant accession numbers or links provided?	Yes	Material and methods section
If publicly available data were reused, provide the respective **data citations in the reference list**.	Not Applicable	
